# PD-1*-cis* IL-2R agonism yields better effectors from stem-like CD8^+^ T cells

**DOI:** 10.1038/s41586-022-05192-0

**Published:** 2022-09-28

**Authors:** Laura Codarri Deak, Valeria Nicolini, Masao Hashimoto, Maria Karagianni, Petra C. Schwalie, Laura Lauener, Eleni Maria Varypataki, Marine Richard, Esther Bommer, Johannes Sam, Stefanie Joller, Mario Perro, Floriana Cremasco, Leo Kunz, Emilio Yanguez, Tamara Hüsser, Ramona Schlenker, Marisa Mariani, Vinko Tosevski, Sylvia Herter, Marina Bacac, Inja Waldhauer, Sara Colombetti, Xavier Gueripel, Stephan Wullschleger, Melanie Tichet, Douglas Hanahan, Haydn T. Kissick, Stephane Leclair, Anne Freimoser-Grundschober, Stefan Seeber, Volker Teichgräber, Rafi Ahmed, Christian Klein, Pablo Umaña

**Affiliations:** 1 Roche Innovation Center Zurich, Schlieren, Switzerland; 2 Emory Vaccine Center and Department of Microbiology and Immunology, Emory University School of Medicine, Atlanta, GA USA; 3 Roche Innovation Center Basel, Basel, Switzerland; 4 Roche Innovation Center Munich, Penzberg, Germany; 5 Swiss Institute for Experimental Cancer Research (ISREC), School of Life Sciences, EPFL, Lausanne, Switzerland; 6 Swiss Cancer Center Leman (SCCL), Lausanne, Switzerland; 7 Ludwig Institute for Cancer Research, Lausanne Branch, Lausanne, Switzerland; 8 Agora Translational Cancer Research Center, Lausanne, Switzerland; 9 Department of Urology, Emory University School of Medicine, Atlanta, GA USA; 10 Winship Cancer Institute of Emory University, Atlanta, GA USA

**Keywords:** Cancer immunotherapy, Tumour immunology, Interleukins, Infection, Preclinical research

## Abstract

Expansion and differentiation of antigen-experienced PD-1^+^TCF-1^+^ stem-like CD8^+^ T cells into effector cells is critical for the success of immunotherapies based on PD-1 blockade^1–4^. Hashimoto et al. have shown that, in chronic infections, administration of the cytokine interleukin (IL)-2 triggers an alternative differentiation path of stem-like T cells towards a distinct population of ‘better effector’ CD8^+^ T cells similar to those generated in an acute infection^5^. IL-2 binding to the IL-2 receptor α-chain (CD25) was essential in triggering this alternative differentiation path and expanding better effectors with distinct transcriptional and epigenetic profiles. However, constitutive expression of CD25 on regulatory T cells and some endothelial cells also contributes to unwanted systemic effects from IL-2 therapy. Therefore, engineered IL-2 receptor β- and γ-chain (IL-2Rβγ)-biased agonists are currently being developed^6–10^. Here we show that IL-2Rβγ-biased agonists are unable to preferentially expand better effector T cells in cancer models and describe PD1-IL2v, a new immunocytokine that overcomes the need for CD25 binding by docking in *cis* to PD-1. *Cis* binding of PD1-IL2v to PD-1 and IL-2Rβγ on the same cell recovers the ability to differentiate stem-like CD8^+^ T cells into better effectors in the absence of CD25 binding in both chronic infection and cancer models and provides superior efficacy. By contrast, PD-1- or PD-L1-blocking antibodies alone, or their combination with clinically relevant doses of non-PD-1-targeted IL2v, cannot expand this unique subset of better effector T cells and instead lead to the accumulation of terminally differentiated, exhausted T cells. These findings provide the basis for the development of a new generation of PD-1 *cis*-targeted IL-2R agonists with enhanced therapeutic potential for the treatment of cancer and chronic infections.

## Abstract (web summary)

Binding of the PD1-IL2v immunocytokine to PD-1 and IL-2Rβγ on the same cell leads to an alternative differentiation of stem-like CD8^+^ T cells into better effectors rather than exhausted T cells in models of both chronic infection and cancer.

## Main

Our previous work has shown that interleukin (IL)-2 therapy synergizes with anti-PD-L1 therapy to enhance lymphocytic choriomeningitis virus (LCMV)-specific CD8^+^ T cells and improve viral control during chronic infection^11^. However, there are concerns regarding the use of IL-2 to enhance immune responses, including its activity on lung endothelial cells and CD4^+^CD25^+^ regulatory T (T_reg_) cells through binding to CD25, leading to vascular leak syndrome including pulmonary oedema and to preferential expansion of T_reg_ cells, respectively. To overcome these limitations, a new class of IL-2 receptor β- and γ-chain (IL-2Rβγ)-biased agonists is currently being developed, some of which are additionally targeted to cell-surface proteins overexpressed in tumours or surrounding stroma to enhance their local tumour retention, such as CEA-IL2v^8^ and FAP-IL2v^12,13^.

## No synergy of muFAP-IL2v with anti-PD-L1 therapy

**Extended Data Fig. 1:**
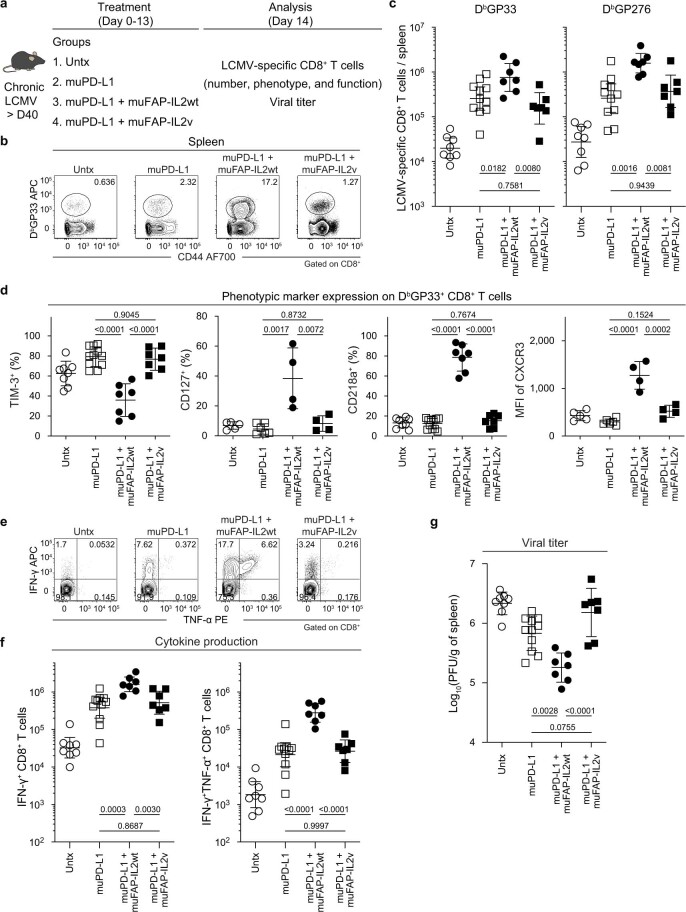
MuFAP-IL2v fails to synergize with muPD-L1 therapy during chronic LCMV infection. **a**, Chronically LCMV-infected mice (> day 40 post-infection) were left untreated or treated with muPD-L1, muPD-L1 + muFAP-IL2wt, and muPD-L1 + muFAP-IL2v therapy for 2 weeks and then CD8^+^ T-cell responses and viral titer were examined. **b**, Representative FACS plots for D^b^GP33^+^ CD8^+^ T cells in spleen. **c**, Numbers of D^b^GP33^+^ and D^b^GP276^+^ CD8^+^ T cells. **d**, Phenotypic marker expression on D^b^GP33^+^ CD8^+^ T cells. **e**, Representative FACS plots for IFN-γ^+^ and INF-γ^+^TNF-α^+^ LCMV-specific CD8^+^ T cells. **f**, Numbers of IFN-γ^+^ and INF-γ^+^TNF-α^+^ LCMV-specific CD8^+^ T cells. **g**, Viral titer in spleen. Results were pooled from 2-3 experiments with n = 2–5 mice per group in each experiment. Data are presented as geometric mean and 95% CI (**c**,**f**) or mean and SD (**d**,**g**) with p values. Statistical comparisons were performed using one-way ANOVA with Tukey’s multiple comparison test. Untx, untreated; AF, Alexa Fluor. Source Data

We therefore compared the therapeutic efficacy of mouse FAP-IL2wt (muFAP-IL2wt), with intact CD25 binding, and mouse FAP-IL2v (muFAP-IL2v) in combination with mouse anti-PD-L1 (muPD-L1) therapy during chronic LCMV infection (Extended Data Fig. 1a). We found that muFAP-IL2wt therapy synergized with muPD-L1 therapy to enhance LCMV-specific CD8^+^ T cell responses as indicated by the expansion of DbGP33^+^ and DbGP276^+^ CD8^+^ T cells (Extended Data Fig. 1b,c). Conversely, muPD-L1 in combination with muFAP-IL2v was not superior to muPD-L1 monotherapy in increasing the numbers of LCMV-specific CD8^+^ T cells (Extended Data Fig. 1b,c). In addition to its quantitative advantage over muPD-L1 monotherapy, muPD-L1 in combination with muFAP-IL2wt changed the expression of various phenotypic markers on LCMV-specific CD8^+^ T cells (Extended Data Fig. 1d). muPD-L1 and muFAP-IL2wt combination therapy elevated the expression levels of CD127, CD218a and CXCR3 on LCMV-specific CD8^+^ T cells, all of which are critical molecules for functional effector and memory CD8^+^ T cell differentiation during acute infection. By contrast, expression of the inhibitory receptor TIM-3 was lower on LCMV-specific CD8^+^ T cells after muPD-L1 and muFAP-IL2wt combination therapy. These phenotypic changes achieved by adding muFAP-IL2wt to muPD-L1 therapy were absent when combining muPD-L1 with muFAP-IL2v (Extended Data Fig. 1d). Expanded LCMV-specific CD8^+^ T cells obtained after muPD-L1 and muFAP-IL2wt therapy were also more functional in their effector profiles of cytokine production than those obtained from muPD-L1 monotherapy in response to antigenic stimulation, whereas muFAP-IL2v administration had no additive effects to muPD-L1 therapy (Extended Data Fig. 1e,f). Notably, the most effective viral control was observed when combining muPD-L1 with muFAP-IL2wt therapy. By contrast, muFAP-IL2v treatment did not show synergy with muPD-L1 therapy in terms of viral reduction (Extended Data Fig. 1g).

**Extended Data Fig. 2:**
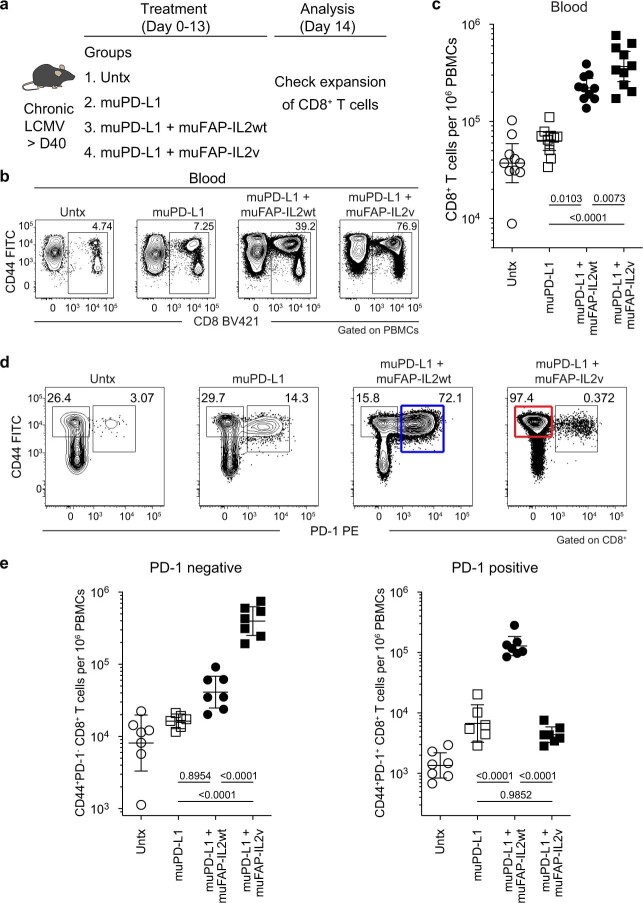
MuFAP-IL2v is biologically active *in vivo* but non-specifically expands CD8 T cells. **a**, Chronically LCMV-infected mice (> day 40 post-infection) were left untreated or treated with muPD-L1, muPD-L1 + muFAP-IL2wt, and muPD-L1 + muFAP-IL2v for 2 weeks and then analyzed for expansion of CD8 T cells. **b**, Representative FACS plots for CD8^+^ T cells in PBMCs. **c**, Numbers of CD8^+^ T cells per 10^6^ PBMCs. **d**, Representative FACS plots for CD44 and PD-1 expressions on CD8^+^ T cells in PBMCs. **e**, Numbers of CD44^+^PD-1^−^ and CD44^+^PD-1^+^ CD8^+^ T cells per 10^6^ PBMCs. Results were pooled from 3-4 experiments with n = 2–5 mice per group in each experiment. Data are presented as geometric mean and 95% CI with p values. Statistical comparisons were performed using one-way ANOVA with Tukey’s multiple comparison test. Untx, untreated. Source Data

It is important to note that muFAP-IL2v was biologically active in vivo, as muPD-L1 in combination with muFAP-IL2v significantly increased the number of total CD8^+^ T cells compared with muPD-L1 as monotherapy or in combination with muFAP-IL2wt during chronic LCMV infection (Extended Data Fig. 2a–c). However, when we characterized the increased number of CD8^+^ T cells, we found that combination of muPD-L1 with muFAP-IL2v mainly expanded non-LCMV-specific PD-1^–^CD8^+^ T cells during chronic infection (Extended Data Fig. 2d,e). This was in marked contrast to the muPD-L1 and muFAP-IL2wt combination, which preferentially expanded PD-1^+^CD8^+^ T cells that included LCMV-specific CD8^+^ T cells (Extended Data Fig. 2d,e). Expansion of non-LCMV-specific CD8^+^ T cells by muPD-L1 and muFAP-IL2v combination therapy implied a requirement for targeted delivery of IL-2v to PD-1-expressing LCMV-specific CD8^+^ T cells to achieve desirable biological outcomes.

## PD1-IL2v mediates *cis* delivery of IL-2v to PD-1^+^ T cells

**Fig. 1:**
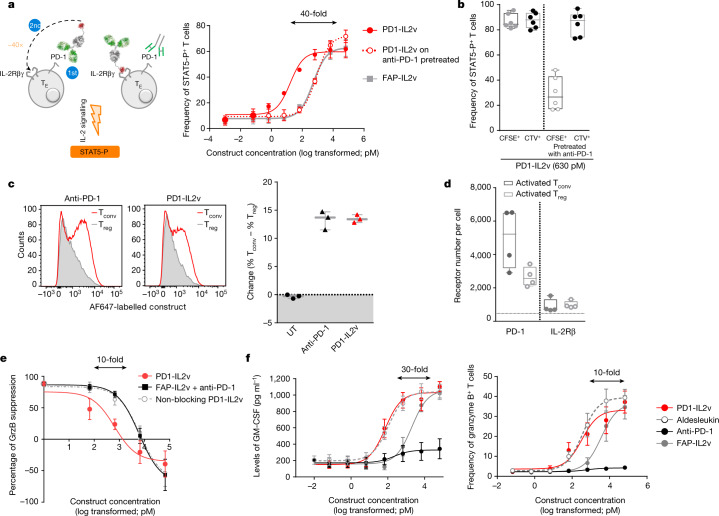
PD1-IL2v mediates *cis* delivery of IL-2v to PD-1^+^ T cells, providing preferential stimulation of PD-1^+^ T cells, overcoming T_reg_-mediated suppression and inducing T cell effector functions. **a**, Frequency of in vitro-activated polyclonal human STAT5-P^+^CD4^+^ T cells following exposure for 12 min to increasing concentrations of either PD1-IL2v or FAP-IL2v. As an additional control, a portion of the PD-1^+^ T cells were pretreated with anti-PD-1 antibody to prevent PD-1-mediated targeting of PD1-IL2v (dotted line) (*n* = 3 healthy donors, 3 independent experiments; mean ± s.e.m.). T_E_, effector T cell; αPD-1, anti-PD-1 antibody. Arrows indicate the difference in potency. **b**, Frequency of in vitro-activated polyclonal human STAT5-P^+^CD4^+^ T cells following exposure for 12 min to 630 pM PD1-IL2v of CFSE-labelled PD-1^+^ or PD-1-preblocked (PD-1^–^) T cells co-cultured with CTV-labelled PD-1^+^ T cells (*n* = 6 healthy donors, 3 independent experiments; box plots represent the median, minimum/maximum and individual points). **c**, Left, flow cytometry histogram plots of binding competition of directly conjugated anti-PD-1 antibody or PD1-IL2v to human CD4^+^ T_conv_ versus T_reg_ cells, cultured together, from one representative donor of three. Right, change in the frequency of human CD4^+^ T_conv_ and T_reg_ cells stained with labelled anti-PD-1 antibody or PD1-IL2v (*n* = 3 healthy donors, 3 independent experiments; mean ± s.e.m.). UT, untreated. **d**, Number of PD-1 receptors and IL-2Rβ per T cell on T_conv_ and T_reg_ cells (*n* = 4 healthy donors; box plots represent the median, minimum/maximum and individual points). **e**, T_reg_ suppression of T_conv_ secretion of granzyme B (GrzB) in the presence of increasing concentrations of PD1-IL2v, FAP-IL2v in combination with anti-PD-1 antibody, and non-blocking PD1-IL2v (*n* = 5 healthy donors, 5 independent experiments; mean ±s.e.m.). **f**, Dose-dependent GM-CSF and granzyme B secretion by in vitro-activated polyclonal human CD4^+^ T cells following stimulation for 5 d with increasing concentrations of PD1-IL2v, aldesleukin, FAP-IL2v or anti-PD-1 antibody (*n* = 4 healthy donors, 2 independent experiments; mean ± s.e.m.).

PD-1 is expressed on the surface of chronically activated antigen-specific T cells, including virus- and tumour-reactive T cells, and is a bona fide marker to identify antigen-specific T cells^14–16^. We designed PD1-IL2v to provide IL-2R agonism preferentially to PD-1^+^ tumour-reactive T cells by binding and blocking the PD-1 inhibitory pathway while agonizing IL-2R signalling on the same cell. To measure the potency of PD1-IL2v versus FAP-IL2v, used here as IL-2v not targeted to T cells, we briefly incubated in vitro-activated PD-1-expressing polyclonal human CD4^+^ T cells with increasing amounts of either PD1-IL2v or FAP-IL2v before measuring IL-2R signalling through the levels of phosphorylated STAT5 (STAT5-P). In this assay, PD1-IL2v was found to be approximately 40-fold more potent than FAP-IL2v in delivering IL-2R agonism to PD-1^+^ T cells (Fig. 1a). To verify that PD-1 targeting mediated the observed difference in potency between the two compounds, we included a group of activated T cells pre-incubated with an excess of the parental PD-1-blocking antibody competing with PD1-IL2v for binding to PD-1. When PD-1-mediated targeting was prevented, the potency of PD1-IL2v became comparable to that of FAP-IL2v (Fig. 1a).

To assess whether PD1-IL2v is delivering IL-2v to IL-2Rβγ on the same PD-1-expressing T cell, we developed a ‘*cis* versus *trans*’ assay in which in vitro-activated polyclonal human CD4^+^ T cells, with homogenous PD-1 expression, were divided into two groups and labelled with two different membrane dyes: carboxyfluorescein succinimidyl ester (CFSE) and CellTrace Violet (CTV). CFSE-labelled T cells were further subdivided into two groups, one of which was pre-incubated with parental anti-PD-1 antibody at a saturating concentration before being co-cultured with PD-1^+^ T cells labelled with CTV. Interestingly, following exposure to PD1-IL2v, the frequency of STAT5-P^+^ T cells was only roughly 25% in T cells with preblocked PD-1, even though they were in close proximity to non-blocked PD-1^+^ T cells, which were virtually all STAT5-P^+^ (Fig. 1b). Therefore, PD1-IL2v delivers IL-2R agonism in *cis* on the same T cell following binding to the PD-1 receptor and not in *trans* to adjacent cells. Taken together, these data show that preferential *cis* targeting of PD1-IL2v results in enhanced potency on PD-1^+^ T cells.

## PD1-IL2v rescues T cells from T_reg_ suppressive function

T_reg_ cells represent a key subset of T cells able to infiltrate the tumour microenvironment and create an immunosuppressive milieu limiting the anti-tumour immune response^17,18^. In addition, T_reg_ cells represent a critical liability for immunotherapies based on IL-2 because of their constitutively high expression of CD25, resulting in their detrimental proliferation and suppressive function^19,20^. For this reason, we performed a binding competition assay in which equal numbers of naturally occurring T_reg_ cells (CD4^+^CD25^+^FOXP3^+^) and conventional CD4^+^ T (T_conv_) cells, from human peripheral blood, were labelled with different membrane dyes and cultured together. Subsequently, the cells were activated in vitro for 3 d before being exposed to a non-saturating concentration of directly labelled PD1-IL2v or the parental anti-PD-1 antibody. PD1-IL2v, similarly to the anti-PD-1 antibody, preferentially bound to T_conv_ rather than T_reg_ cells (Fig. 1c). In agreement with this finding, we observed an approximately twofold-higher number of PD-1 receptors per T cell on T_conv_ cells than on T_reg_ cells (Fig. 1d).

In a T_reg_ suppression assay, in which suppression is measured as the ability of T_reg_ cells to dampen T_conv_ effector functions such as granzyme B secretion, the preferential binding of PD1-IL2v to T_conv_ cells allowed them to overcome T_reg_-mediated suppression in a dose-dependent manner. As controls, we used an alternative PD1-IL2v molecule, called ‘non-blocking PD1-IL2v’, comprising an anti-PD-1 moiety with non-PD-L1/PD-L2-blocking function, to simply deliver IL-2v to PD-1^+^ T cells, as well as the combination of the parental blocking anti-PD-1 antibody with FAP-IL2v, to block the PD-1 pathway in the absence of PD-1-mediated delivery of IL-2v to PD-1^+^ T cells. At a concentration of 630 pM, PD1-IL2v overcame T_reg_-mediated suppression, and higher concentrations even further elicited the effector functions of T_conv_ cells, regardless of the presence of T_reg_ cells (Fig. 1e). Both non-blocking PD1-IL2v and the combination of parental anti-PD-1 antibody with FAP-IL2v only achieved a similar effect at tenfold-higher concentrations than for PD1-IL2v, indicating that both blockade of PD-1 signalling and PD-1-mediated delivery of IL-2v to PD-1^+^ T cells are critical aspects of the mechanism of action of PD1-IL2v.

## PD1-IL2v is internalized with bound PD-1 receptors

**Extended Data Fig. 3:**
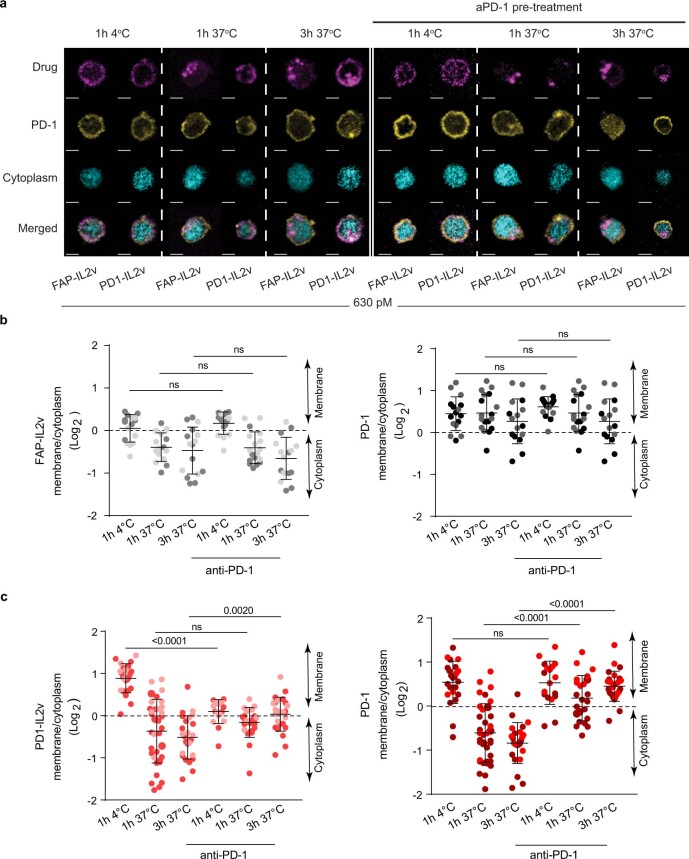
PD1-IL2v is internalized upon binding to PD-1 and IL-2Rβγ and drives the internalization of the bound PD-1 receptors. **a**. Representative confocal images of IL2v and PD-1 receptor internalization kinetics at 37 °C in in vitro activated polyclonal PD-1^+^ CD4 T cells upon incubation with 630 pM of PD1-IL2v or FAP-IL2v for 1 or 3 h, with or without anti-PD1 pre-treatment to prevent PD-1 binding by PD1-IL2v. PD1-IL2v and FAP-IL2v are in pink, PD-1 surface staining in yellow, and the cytoplasm is in cyan. **b**–**c**. Quantification of average drug intensity in membrane/cytoplasm (Log_2_). Log_2_ = 0: Equal amount of drug at the membrane and in the cytoplasm (dotted line). Log_2_>0: More drug is on the membrane. Log_2_<0: Drug localizes in the cytoplasm. Each dot represents quantification from a single CD4 T cell; clear and dark dots indicate T cells derived from two different donors, from 2 independent experiments. Mean and SD are shown. One-way ANOVA with a post hoc Tukey multiple comparison test.

Given that, following binding to IL-2, T cells internalize IL-2R within minutes^21^, we assessed the internalization of fluorescently labelled PD1-IL2v and of FAP-IL2v as a control using in vitro-activated PD-1-expressing polyclonal human CD4^+^ T cells. Additionally, we tracked the fate of simultaneously bound PD-1 receptors using a fluorescently labelled, non-competing anti-PD-1 antibody. We observed that, while FAP-IL2v was internalized within 1 h at 37 °C (Extended Data Fig. 3a,b), PD1-IL2v was internalized with slower kinetics (Extended Data Fig. 3a,c). Interestingly, slower PD1-IL2v internalization was accompanied by simultaneous internalization of bound PD-1 receptors (Extended Data Fig. 3c). Pretreatment of PD-1^+^ T cells with a competing anti-PD-1 antibody prevented binding of PD1-IL2v to PD-1, therefore inducing PD1-IL2v internalization through the IL-2R at rates similar to those induced by FAP-IL2v, while leaving the PD-1 receptors on the T cell surface (Extended Data Fig. 3c). Pretreatment with the anti-PD-1 antibody did not affect FAP-IL2v internalization or surface expression of PD-1 receptors (Extended Data Fig. 3b).

These data suggest an unexpected additional mechanism of action of PD1-IL2v at a cellular level, where longer interaction of IL-2v with IL-2R could result in continuous signalling followed by internalization and removal of bound PD-1 receptors from the T cell surface.

## PD1-IL2v potently drives T cell effector functions

**Extended Data Fig. 4:**
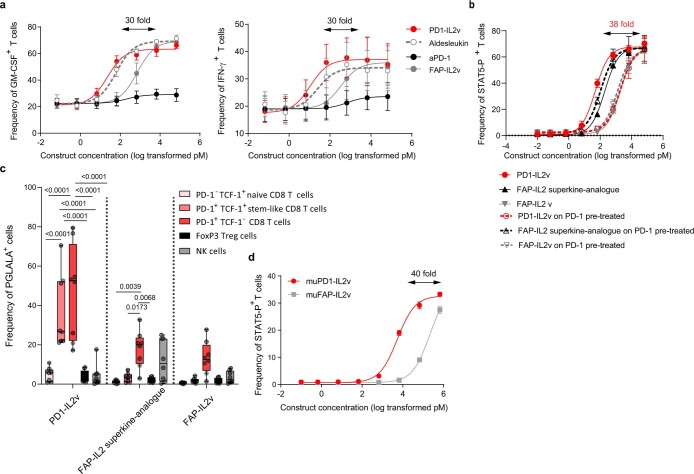
*In vitro* CD4 T cell activation and cytokine release by PD1-IL2v and *in vivo* expansion of antigen-specific polyfunctional CD8 T cells by muPD1-IL2v. **a**. Dose dependent increase in frequencies of GM-CSF^+^ and IFN-γ^+^ human polyclonal CD4 T cells upon 5 days of *in vitro* stimulation with increasing concentrations of either PD1-IL2v, Aldesleukin, FAP-IL2v or PD-1 antibody (n = 4 healthy donors, 2 independent experiments, mean ± SEM). **b**. Frequency of *in vitro* activated, polyclonal human STAT5-P^+^ CD4 T cells upon exposure for 12 min to increasing concentrations of either PD1-IL2v, FAP-IL2v or FAP-IL2 superkine-analogue. As additional control, part of the PD-1^+^ T cells were pre-treated with PD-1 antibody to prevent PD-1-mediated targeting of PD1-IL2v (dotted line) (n = 2 donors from 2 independent experiments, mean ± SEM). **c**. Targeting of several T cell subsets and NK cells from fresh PBMCs by PD1-IL2v, FAP-IL2v and FAP-IL2 superkine-analogue (n = 8 healthy donors from 4 independent experiments, box plots representing median, minimum/maximum and individual points). Statistical comparisons were performed using two-way ANOVA with Tukey’s multiple comparison test. **d**. Frequency of *in vitro* activated, STAT5-P^+^ murine CD4 T cells upon exposure for 12 min to increasing concentrations of either muPD1-IL2v or muFAP-IL2v *in vitro* (n = 2 mice from 2 independent experiments, mean ± SEM.

IL-2 has been shown to induce secretion of granulocyte-macrophage colony-stimulating factor (GM-CSF) by T cells^22^, which is important for dendritic cell activation and maturation, in addition to enhancing T cell cytotoxic effector functions^23^. For this reason, we tested whether PD1-IL2v could also elicit GM-CSF secretion, in addition to granzyme B, from PD-1-expressing polyclonal human CD4^+^ T cells activated in vitro for 5 d. As expected, PD1-IL2v induced GM-CSF and granzyme B secretion by activated T cells in a dose-dependent fashion and was roughly 30-fold more potent than untargeted FAP-IL2v, while PD-1 blockade alone did not induce any significant change in effector functions. Interestingly, PD1-IL2v was as potent as wild-type IL-2 with intact CD25 binding (aldesleukin) in eliciting T cell effector functions, in line with the hypothesis that PD-1-mediated *cis* delivery of IL-2v acts as a surrogate of CD25 for *cis* binding on the T cell surface (Fig. 1f and Extended Data Fig. 4a).

## Comparison of PD1-IL2v to IL-2Rb-biased IL-2 mutant

An alternative approach to engineering IL-2 for systemic therapy is to increase its affinity for IL-2Rβ, with the aim of making signalling less dependent on *cis* anchoring through CD25. One such engineered IL-2 has been termed ‘superkine’ (ref. ^24^). This is a different approach as compared with targeting cytokines to specific immune cells by fusion to immune receptor-targeting antibodies such as in PD1-IL2v or other recently reported fusion proteins^25,26^. We therefore produced a FAP-IL2 superkine analogue with increased binding affinity for IL-2Rβ and compared it with PD1-IL2v for potency and *cis* targeting in the *cis–trans* STAT5-P assay in activated T cells. As a control, we used FAP-IL2v, which binds to IL-2Rβγ with an affinity comparable to that of wild-type IL-2 in the absence of IL-2Rα. We observed that the FAP-IL2 superkine analogue was tenfold more potent than FAP-IL2v in delivering IL-2R agonism to T cells. However, both the FAP-IL2 superkine analogue and FAP-IL2v were as active on PD-1^+^ T cells as on PD-1^−^ T cells, regardless of PD-1 expression (Extended Data Fig. 4b). Conversely, PD1-IL2v was roughly 40-fold more potent on PD-1^+^ T cells than on PD-1^−^ T cells, and on PD-1^+^ T cells PD1-IL2v was fivefold more potent than the FAP-IL2 superkine analogue (Extended Data Fig. 4b).

We then extended our observations to more physiologically relevant conditions, by exposing peripheral blood mononuclear cells (PBMCs) from healthy donors to a non-saturating concentration (630 pM) of PD1-IL2v, FAP-IL2 superkine analogue or FAP-IL2v for 30 min before staining with a phycoerythrin (PE)-conjugated anti-PGLALA antibody to detect the bound molecule and with a panel of antibodies to phenotypically characterize the T cell subsets through flow cytometry. PD1-IL2v significantly bound to both PD-1^+^TCF-1^+^ stem-like CD8^+^ T cells and PD1^+^TCF-1^−^CD8^+^ T cells when compared with naive CD8^+^ T cells, T_reg_ cells and natural killer (NK) cells (Extended Data Fig. 4c). By contrast, the FAP-IL2 superkine analogue and FAP-IL2v bound modestly to PD-1^+^TCF-1^−^CD8^+^ T cells and did not bind to PD-1^+^TCF-1^+^ stem-like CD8^+^ T cells, potentially because of their lower IL-2Rβ expression levels. In addition, FAP-IL2 superkine analogue was the only fusion protein to strongly bind to NK cells (Extended Data Fig. 4c).

This finding suggests a fundamental functional difference between targeted delivery of a mutated IL-2 devoid of CD25 binding to antigen-experienced cells that are PD-1^+^ and just engineering IL-2 for increased affinity for its receptor on all cells. The former approach provides increased selectivity for the IL-2R agonism on specific T cell populations, whereas the latter increases the potency of signalling on many cells irrespective of their antigen experience and is only regulated by the expression profile of IL-2R by overall lymphocytes.

## Targeted delivery of IL-2v to PD-1^+^CD8^+^ T cells

**Extended Data Fig. 5:**
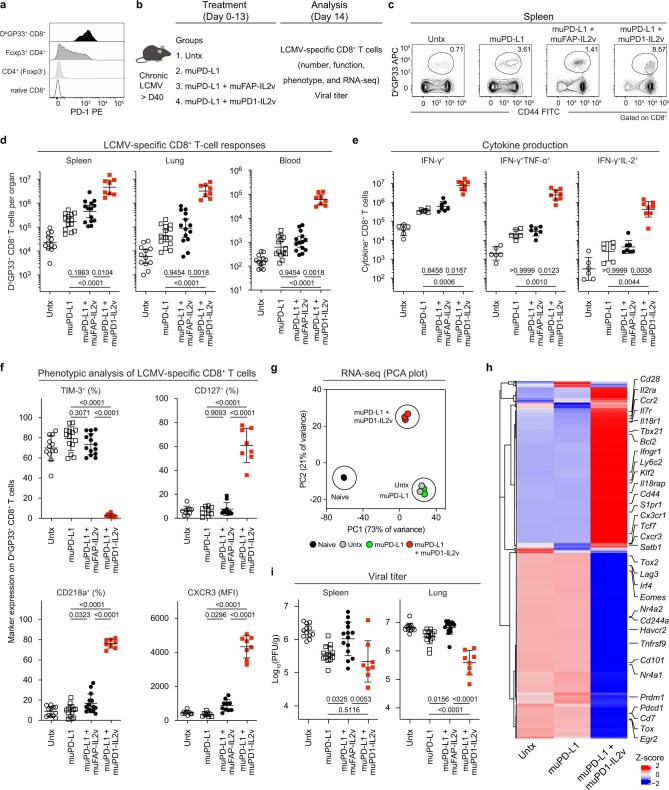
Comparative analysis of muPD1-IL2v versus muFAP-IL2v in combination therapy with muPD-L1 during chronic LCMV infection. **a**, Representative histogram for expression of PD-1 by D^b^GP33^+^ CD8^+^ T cells, Foxp3^+^ CD4^+^ T cells (Tregs), conventional (Foxp3^−^) CD4^+^ T cells, and naïve (CD44^lo^) CD8^+^ T cells. All T cells except naïve CD8^+^ T cells were isolated from spleens of chronically LCMV-infected mice (> day 40 post-infection). Naïve CD8^+^ T cells were isolated from uninfected C57BL/6J mice. The results are representative of two experiments (n = 6 for chronically LCMV-infected mice and n = 2 for uninfected mice). **b**, Chronically LCMV-infected mice (> day 40 post-infection) were treated with muPD-L1, muPD-L1 + muFAP-IL2v, and muPD-L1 + muPD1-IL2v for 2 weeks and then analyzed for CD8 T-cell responses and viral titer. **c**, Representative FACS plots for D^b^GP33^+^ CD8^+^ T cells in spleen. **d**, Numbers of D^b^GP33^+^ CD8^+^ T cells in the indicated tissues. **e**, Numbers of IFN-γ^+^, INF-γ^+^TNF-α^+^, and INF-γ^+^IL-2^+^ LCMV-specific CD8^+^ T cells in spleen. **f**, Phenotypic marker expression on D^b^GP33^+^ CD8^+^ T cells in spleen. **g**, PCA plot of RNA-seq for naïve CD8^+^ T cells from uninfected mice and D^b^GP33^+^ CD8^+^ T cells from chronically LCMV-infected mice after the indicated treatments. **h**, Heat map showing mean relative expressions of all differentially expressed genes (n = 1954) across treatment groups. **i**, Viral titer in the indicated tissues. Results were pooled from 3–5 experiments with n = 2–5 mice per group in each experiment (**c**–**f**, **i**). RNA-seq data for groups of naive, Untx, and muPD-L1 were obtained from GSE206722. RNA-seq data for muPD-L1 + muPD1-IL2v group were generated from biological triplicates with n = 2 mice per replicate (**g**,**h**). Data are presented as geometric mean and 95% CI (**d**,**e**) or mean and SD (**f**,**i**) with p values. Statistical comparisons were performed using Kruskal-Wallis test with Dunn’s multiple comparison test (**d**,**e**), one-way ANOVA with Tukey’s multiple comparison test (**f**,**i**). Untx, untreated. Source Data

Given the effective *cis* delivery of IL-2v to PD-1-expressing T cells by PD1-IL2v in vitro, we wondered whether muPD1-IL2v (Extended Data Fig. 4d) could efficiently deliver IL-2v to LCMV-specific CD8^+^ T cells in vivo during chronic infection. It is worth noting that PD-1 expression was highest on LCMV-specific CD8^+^ T cells compared with other T cell populations during chronic infection (Extended Data Fig. 5a). We compared the therapeutic efficacy of muPD-L1, muPD-L1 in combination with muFAP-IL2v, and muPD-L1 in combination with muPD1-IL2v during chronic infection and performed quantitative and qualitative analyses of LCMV-specific CD8^+^ T cells (Extended Data Fig. 5b). As previously shown, muFAP-IL2v therapy did not have additive effects in comparison to muPD-L1 therapy in terms of enhancing LCMV-specific CD8^+^ T cell responses (Extended Data Fig. 5c–e). Interestingly, combination of muPD-L1 with muPD1-IL2v was significantly superior to muPD-L1 monotherapy in increasing the numbers of LCMV-specific CD8^+^ T cells in all tissues analysed (Extended Data Fig. 5d). Moreover, muPD-L1 in combination with muPD1-IL2v induced qualitative changes in LCMV-specific CD8^+^ T cells, as exemplified by the polyfunctional signature (IFN-γ^+^TNF-α^+^ and IFN-γ^+^IL-2^+^) (Extended Data Fig. 5e), and altered the expression profiles of several phenotypic markers such as TIM-3, CD127, CD218a and CXCR3 (Extended Data Fig. 5f).

To gain further insights into the qualitative attributes of LCMV-specific CD8^+^ T cells after muPD-L1 and muPD1-IL2v combination therapy, we performed a transcriptional analysis by RNA sequencing (RNA-seq) of LCMV-specific CD8^+^ T cells after the treatments. Principal-component analysis (PCA) showed that the transcriptional signature of LCMV-specific CD8^+^ T cells after muPD-L1 monotherapy was very similar to that of the untreated group (Extended Data Fig. 5g). Notably, adding muPD1-IL2v to muPD-L1 therapy changed the transcriptional signature of LCMV-specific CD8^+^ T cells, indicating that combination of muPD-L1 with muPD1-IL2v generated LCMV-specific CD8^+^ T cells that were distinct from those in the untreated or muPD-L1 single-treatment group (Extended Data Fig. 5g). The heatmap of differentially expressed genes across the treatment groups highlights the therapeutic potential of muPD1-IL2v therapy resulting from modulation of the differentiation status of LCMV-specific CD8^+^ T cells during chronic infection (Extended Data Fig. 5h). For example, muPD-L1 in combination with muPD1-IL2v elevated the expression levels of *Cd28*, an essential co-stimulatory molecule for improved CD8^+^ T cell responses to anti-PD-1 therapy^27,28^. Upregulated cytokine receptors included *Il2ra*, *Il7r*, *Il18r1*, *Ifngr1* and *Il18rap*, suggesting that LCMV-specific CD8^+^ T cells generated by muPD-L1 and muPD1-IL2v combination therapy are more responsive to inflammatory cytokines (IL-2, IL-18 and interferon-γ (IFNγ)) and the homeostatic cytokine IL-7, the latter of which is an important cytokine for survival and maintenance of naive and memory CD8^+^ T cells^29,30^. muPD-L1 and muPD1-IL2v therapy also increased the abundance of molecules regulating T cell migration (*Ccr2*, *Cx3cr1* and *Cxcr3*)^31–34^, adhesion (*Ly6c2* and *Cd44*) and egress from lymphoid tissues (*S1pr1* and *Klf2*)^35,36^. All of these features are essential components for functional effector CD8^+^ T cells to respond to various co-stimulatory signals, cytokines and chemokines, followed by their migration to major sites of infection to exert effector functions. Indeed, *Tbx21*, a crucial transcription factor for effector CD8^+^ T cell differentiation^37,38^, was also upregulated by co-administration of muPD-L1 and muPD1-IL2v. Conversely, genes downregulated by muPD-L1 and muPD1-IL2v therapy included *Tox* and *Pdcd1*, which are two major regulators of T cell exhaustion^39,40^. Other inhibitory receptors (*Lag3*, *Cd244a* and *Havcr2*) and transcription factors (*Tox2*, *Nr4a2*, *Nr4a1*, *Prdm1* and *Egr2*) associated with exhausted CD8^+^ T cells^39–41^ were also downregulated by muPD-L1 and muPD1-IL2v combination therapy (Extended Data Fig. 5h). Overall, LCMV-specific CD8^+^ T cells generated by combining muPD-L1 and muPD1-IL2v possessed increased expression of molecules critical for functional effector cells and decreased expression of major transcription factors and inhibitory receptors related to exhausted CD8^+^ T cells, in line with induction of antigen-specific CD8^+^ T cell states with better effector potential and skewed away from T cell exhaustion. Most notably, these quantitative and qualitative changes in LCMV-specific CD8^+^ T cells accomplished by co-treatment with muPD-L1 and muPD1-IL2v were linked to improved biological outcome, and muPD-L1 and muPD1-IL2v therapy resulted in the best viral control across the treatment groups (Extended Data Fig. 5i).

**Fig. 2:**
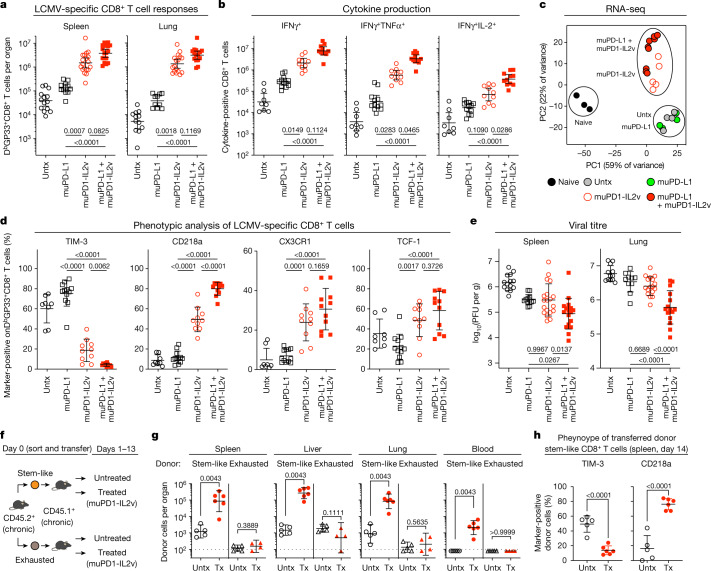
Targeted delivery of IL-2v to PD-1^+^ T cells using the muPD1-IL2v construct increases LCMV-specific CD8^+^ T cell responses and improves viral control during chronic infection by enhancing the proliferation and differentiation of PD-1^+^TCF-1^+^ stem-like resource CD8^+^ T cells. Chronically LCMV-infected mice (more than 40 d after infection) were left untreated (Untx) or treated with muPD-L1, muPD1-IL2v or muPD-L1 + muPD1-IL2v for 2 weeks and then analysed for CD8^+^ T cell responses and viral titre. **a**, Numbers of D^b^GP33^+^CD8^+^ T cells in the indicated tissues. **b**, Number of IFNγ^+^, IFNγ^+^TNFα^+^ and IFNγ^+^IL-2^+^ LCMV-specific CD8^+^ T cells in the spleen. **c**, PCA plot of RNA-seq data for naive CD8^+^ T cells from uninfected mice and D^b^GP33^+^CD8^+^ T cells from chronically LCMV-infected mice after the indicated treatments. **d**, Phenotypic marker expression on D^b^GP33^+^CD8^+^ T cells in the spleen. **e**, Viral titre in the indicated tissues. PFU, plaque-forming units. **f**, Experimental design for T cell transfer experiments. Sorted stem-like (PD-1^+^CXCR5^+^TIM-3^−^) and exhausted (PD-1^+^CXCR5^−^TIM-3^+^) CD8^+^ T cells isolated from CD45.2^+^ chronically LCMV-infected mice (more than 40 d after infection) were adoptively transferred into infection-matched CD45.1^+^ recipient mice, followed by muPD1-IL2v therapy for 2 weeks. **g**, Numbers of donor CD45.2^+^CD8^+^ T cells in various tissues. The dotted line on the *y* axis indicates the limit of detection for the number of donor cells. Tx, treated. **h**, TIM-3 and CD218a expression on transferred donor stem-like CD45.2^+^CD8^+^ T cells in the spleen of recipients after 2 weeks of treatment. Results were pooled from 4–7 experiments with *n* = 2–4 mice per group in each experiment (**a**,**b**,**d**,**e**) or from two experiments with *n* = 4–6 mice per group (**g**,**h**). RNA-seq data are from Extended Data Fig. 5 and additional samples from six experiments to obtain various CD8^+^ T cell populations with *n* = 1–15 mice per group in each experiment (**c**). Data are presented as the geometric mean and 95% confidence interval (CI) (**a**,**b**,**g**) or the mean and s.d. (**d**,**e**,**h**) with *P* values. Statistical comparisons were performed using the Kruskal–Wallis test with Dunn’s multiple-comparisons test (**a**,**b**), one-way ANOVA with Tukey’s multiple-comparisons test (**d**,**e**), the Mann–Whitney test (two tailed) (**g**) or an unpaired two-tailed *t* test (**h**). Source Data

**Extended Data Fig. 6:**
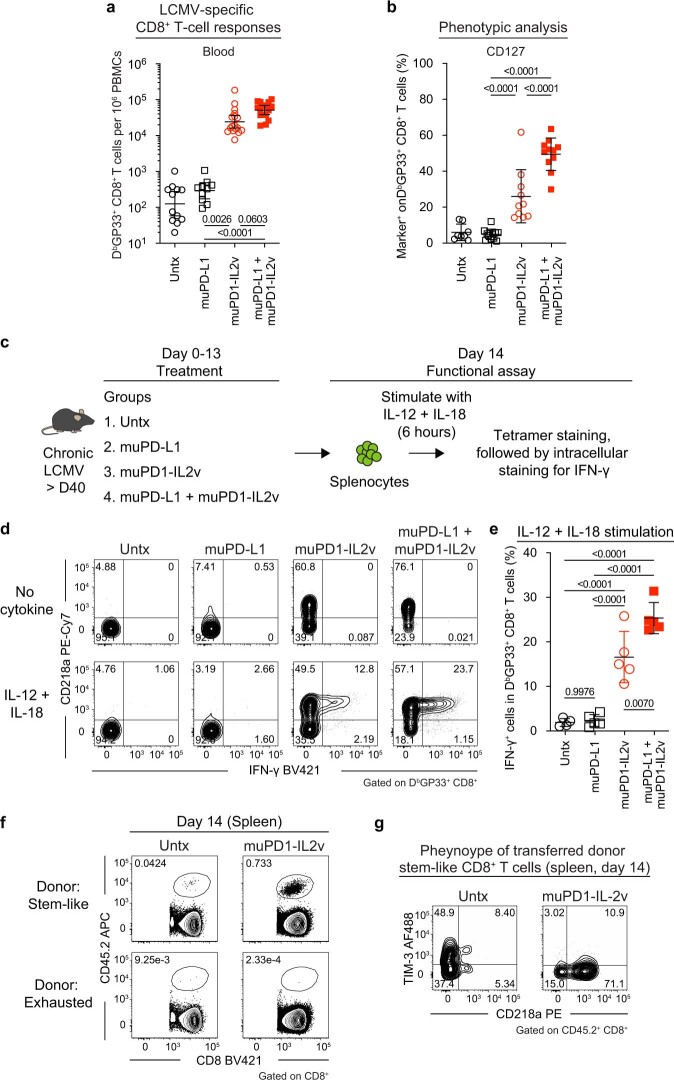
Immunotherapy of chronically LCMV-infected mice with muPD-L1 and muPD1-IL2v. Chronically LCMV-infected mice (> day 40 post-infection) were left untreated or treated with muPD-L1, muPD1-IL2v, and muPD-L1 + muPD1-IL2v for 2 weeks and then analyzed for CD8 T-cell responses. **a**, Numbers of D^b^GP33^+^ CD8^+^ T cells per 1 x10^6^ PBMCs. **b**, CD127 expression on D^b^GP33^+^ CD8^+^ T cells in spleen. **c**, Spleen cells were isolated from chronically LCMV-infected mice after the indicated treatments for 2 weeks. One million cells were cultured with recombinant mouse IL-12 and IL-18 (20 ng/ml each) for 5 h, then GolgiPlug was added, followed by culturing for 1 h. Cells were stained with surface markers including D^b^GP33-specific tetramer, fixed, and followed by intracellular staining for IFN-γ. **d**, Representative FACS plots for co-staining of CD218a and IFN-γ gated on D^b^GP33^+^ CD8^+^ T cells. **e**, Frequency of IFN-γ^+^ cells among D^d^GP33^+^ CD8^+^ T cells in response to stimulation with IL-12 + IL-18. **f**, **g**, Sorted stem-like (PD-1^+^CXCR5^+^TIM-3^−^) and exhausted (PD-1^+^CXCR5^−^TIM-3^+^) CD8^+^ T cells isolated from CD45.2^+^ chronically LCMV-infected mice (> 40 days post-infection) were adoptively transferred into infection-matched CD45.1^+^ recipient mice, followed by muPD1-IL2v therapy for 2 weeks. Representative FACS plots for the frequency of donor CD45.2^+^ cells (**f**) and TIM-3 and CD218a expression on transferred donor stem-like CD45.2^+^ CD8^+^ T cells in spleen of recipients after 2 weeks of the treatments (**g**). Results were pooled from 2–6 experiments with n = 2–4 mice per group in each experiment. Data are presented as geometric mean and 95% CI (**a**) or mean and SD (**b**,**e**) with p values. Statistical comparisons were performed using Kruskal-Wallis test with Dunn’s multiple comparison test (**a**) or one-way ANOVA with Tukey’s multiple comparison test (**b**,**e**). Untx, untreated; AF, Alexa Fluor. Source Data

Interestingly, muPD1-IL2v monotherapy was sufficient to elicit the expansion of LCMV-specific CD8^+^ T cells in different organs (Fig. 2a and Extended Data Fig. 6a). However, combination with muPD-L1 was even more effective at increasing the numbers of polyfunctional LCMV-specific CD8^+^ T cells and imprinting marked phenotypic and transcriptional changes that were induced by muPD1-IL2v therapy (Fig. 2b–d and Extended Data Fig. 6b), resulting in significantly improved viral control in comparison to muPD1-IL2v monotherapy (Fig. 2e).

Finally, we assessed the responsiveness to IL-12 and IL-18 of the LCMV-specific CD8^+^ T cells generated following muPD-L1 and muPD1-IL2v co-treatment and the respective monotherapies. Splenocytes from in vivo-treated mice were briefly stimulated ex vivo with both cytokines before measuring the secretion of IFNγ by D^b^GP33^+^CD8^+^ T cells (Extended Data Fig. 6c). Interestingly, among the LCMV-specific CD8^+^ T cells obtained from mice treated with muPD1-IL2v monotherapy or muPD1-IL2 in combination with muPD-L1, a subset of T cells expressing the receptor for IL-18 rapidly secreted IFNγ after being exposed to IL-12 and IL-18 (Extended Data Fig. 6d,e).

These results together illustrate that targeted delivery of IL-2v to PD-1^+^CD8^+^ T cells by muPD1-IL2v therapy was highly effective in enhancing LCMV-specific CD8^+^ T cell responses with a transcriptional signature of better effectors. In addition, combination of muPD1-IL2v with muPD-L1 further improved some effector attributes such as polyfunctionality as compared with muPD1-IL2v monotherapy and was particularly effective at viral control during chronic infection in this model.

## muPD1-IL2v acts on PD-1^+^TCF-1^+^ stem-like CD8^+^ T cells

During chronic infection, PD-1^+^ LCMV-specific CD8^+^ T cells are a heterogeneous cell population with distinct biological features, and the stem-like (TIM-3^−^TCF-1^+^) and terminally differentiated (exhausted; TIM-3^+^TCF-1^−^) subsets are two major components^16,40,42–45^. Stem-like CD8^+^ T cells act as resource cells to maintain the pools of LCMV-specific CD8^+^ T cells by self-renewal as well as by providing terminally differentiated (exhausted) CD8^+^ T cells to peripheral tissues of major sites of infection. It is also the stem-like subset that provides the proliferative burst of PD-1^+^ LCMV-specific CD8^+^ T cells during anti-PD-L1 therapy in chronic infection^16,42,43^.

To elucidate which CD8^+^ T cell subset is targeted by muPD1-IL2v, we performed adoptive transfer experiments. Two PD-1^+^CD8^+^ T cell subsets, stem-like (PD-1^+^CXCR5^+^TIM-3^−^) and terminally differentiated (exhausted; PD-1^+^CXCR5^−^TIM-3^+^), were sorted from the pooled splenocytes of chronically LCMV-infected mice (CD45.2^+^), and each CD8^+^ T cell subset was transferred into infection-matched mice (CD45.1^+^), followed by muPD1-IL2v therapy. After 2 weeks of treatment, congenically marked CD45.2^+^ donor cells were checked in recipient CD45.1^+^ mice (Fig. 2f). Notably, we found that the proliferative burst came exclusively from the stem-like CD8^+^ T cell subset after muPD1-IL2v therapy, whereas the exhausted CD8^+^ T cell subset did not expand in multiple tissues (Fig. 2g and Extended Data Fig. 6f).

Two weeks after transfer in untreated recipient mice, stem-like donor CD45.2^+^ cells maintained a TIM-3^−^ population, but they also converted to TIM-3^+^ cells, indicating their self-renewal and differentiation potential (Fig. 2h and Extended Data Fig. 6g). Both of these TIM-3^−^ and TIM-3^+^ compartments expressed minimal levels of CD218a, suggesting that during chronic infection the transferred stem-like T cells went through a conventional differentiation pathway from stem-like to terminally differentiated (exhausted) CD8^+^ T cells (Fig. 2h and Extended Data Fig. 6g). By contrast, muPD1-IL2v therapy altered this differentiation process and transferred stem-like T cells underwent optimal effector differentiation, represented by marked upregulation of CD218a with low to intermediate expression of TIM-3 (Fig. 2h and Extended Data Fig. 6g). These results together demonstrate that muPD1-IL2v therapy acts on stem-like CD8^+^ T cells, enhancing their proliferation and effector differentiation.

## muPD1-IL2v eradicates mouse pancreatic tumours

**Fig. 3:**
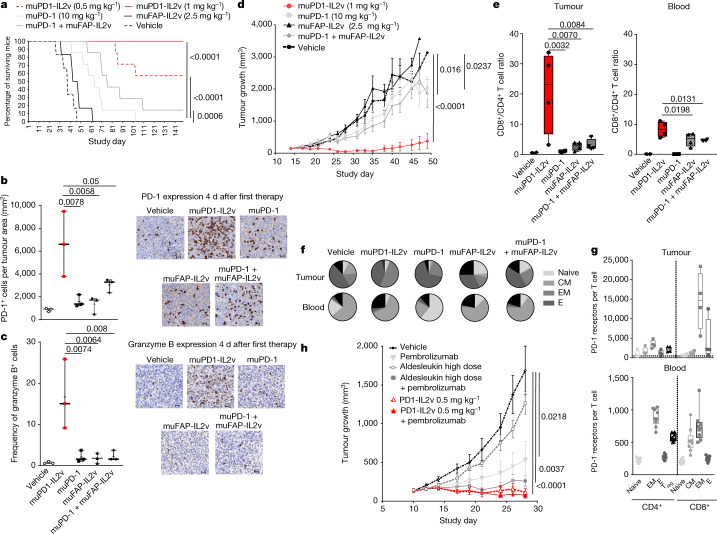
muPD1-IL2v favours CD8^+^ versus CD4^+^ T cells in the tumour microenvironment and expands less differentiated TILs, which provide tumour eradication and survival benefit to treated mice. In vivo efficacy study in syngeneic or human PD-1-transgenic mice bearing orthotopic or subcutaneous Panc02-H7-Fluc tumours treated for 4 or 2 weeks, respectively, with the indicated treatment options. **a**, Survival curve, in days, of control syngeneic mice and mice receiving the indicated therapies bearing an orthotopic tumour (*n* = 7 mice per treatment group). **b**,**c**, Number of PD-1^+^ cells (**b**) and frequency of granzyme B^+^ cells (**c**) within the tumour by immunohistochemistry; scale bars, 20 μm  (*n* = 3; box plots represent median, minimum/maximum and individual points). **d**, Tumour growth curves of subcutaneous tumours in syngeneic control mice and mice treated with the indicated therapies (*n* = 6 mice per treatment group; mean ± s.e.m.). **e**,**f**, CD8^+^ to CD4^+^ T cell ratio (**e**) and T cell differentiation state (**f**) in the tumour and blood of mice across different treatment groups (*n* = 4; box plots represent the median, minimum/maximum and individual points). CM, central memory; E, effector; EM, effector memory; other cells are in black. **g**, Quantification of PD-1 receptors per cell on the surface of T cells isolated from the tumours and blood of untreated human PD-1-transgenic mice (*n* = 4 and *n* = 9 mice, respectively, from more than two independent experiments; box plots represent the median, minimum/maximum and individual points). **h**, Tumour growth curves of subcutaneous tumours in human PD-1-transgenic mice receiving the respective therapies (*n* = 7–12 mice per treatment group; mean ± s.e.m.). In **a**–**f** and **h**, *n* ≥ 3 independent experiments. To test for significant differences in tumour growth inhibition between group means for multiple comparisons, standard ANOVA (one-way ANOVA) was used with Dunnett’s post hoc test in the Panc02 mouse tumour model. Wilcoxon’s test was used for survival analysis of the orthotopic Panc02 mouse tumour model. Statistical comparisons among multiple immuno-pharmacodynamic groups were performed using one-way ANOVA with Tukey’s multiple-comparisons test. Source Data

We then assessed muPD1-IL2v in an in vivo efficacy study in C57BL/6 mice implanted orthotopically with the pancreatic adenocarcinoma syngeneic cell line Panc02-H7-Fluc. Mice were treated once a week for 4 weeks with muPD1-IL2v (0.5 and 1 mg kg^–1^), muPD-1 antibody (10 mg kg^–1^), muFAP-IL2v (2.5 mg kg^–1^) or combinations thereof. muPD1-IL2v eradicated tumours in treated animals and provided long-term survival benefit in four of seven and seven of seven treated mice at doses of 0.5 and 1 mg kg^–1^, respectively (Fig. 3a). Only one mouse from the group treated with parental muPD-1 antibody in combination with muFAP-IL2v survived until the end of the experiment. All mice from the vehicle-treated control group and those receiving muPD-1 antibody or muFAP-IL2v as monotherapy died within 100 d (Fig. 3a).

Immunohistochemical analysis for the expression of PD-1 and granzyme B by tumours obtained from mice across the different treatments showed that muPD1-IL2v induced a significantly higher number of PD-1^+^ (Fig. 3b) and granzyme B^+^ (Fig. 3c) tumour-infiltrating lymphocytes (TILs) than the other treatments.

## muPD1-IL2v favours CD8^+^ over CD4^+^ TILs

To better characterize the phenotype and function of TILs generated by the muPD1-IL2v treatment, Panc02-H7-Fluc tumour cells were implanted subcutaneously in syngeneic mice. Once tumours reached a size of 200 mm^3^, the mice were treated with muPD1-IL2v, muFAP-IL2v and muPD-1, using the above doses, once a week for 2 weeks and monitored for tumour growth. Treatment with muPD1-IL2v resulted in control of tumour growth and led to tumour eradication in three of six mice, while the other treatments failed to do so, both as monotherapies and in combination (Fig. 3d). Phenotypic characterization of TILs across the different treatment groups showed a significant and preferential ~20-fold expansion of CD8^+^ over CD4^+^ T cells in tumours from mice treated with muPD1-IL2v, compared with the control and other treatment groups (Fig. 3e). Notably, by contrast, the ratio of CD8^+^ to CD4^+^ T cells in blood was ~seven- to eightfold increased and comparable in mice receiving either muPD1-IL2v or muFAP-IL2v, the latter either as monotherapy or in combination with muPD-1 antibody (Fig. 3e). This observation is consistent with the notion of higher PD-1 expression on TILs than peripheral blood T cells.

**Extended Data Fig. 7:**
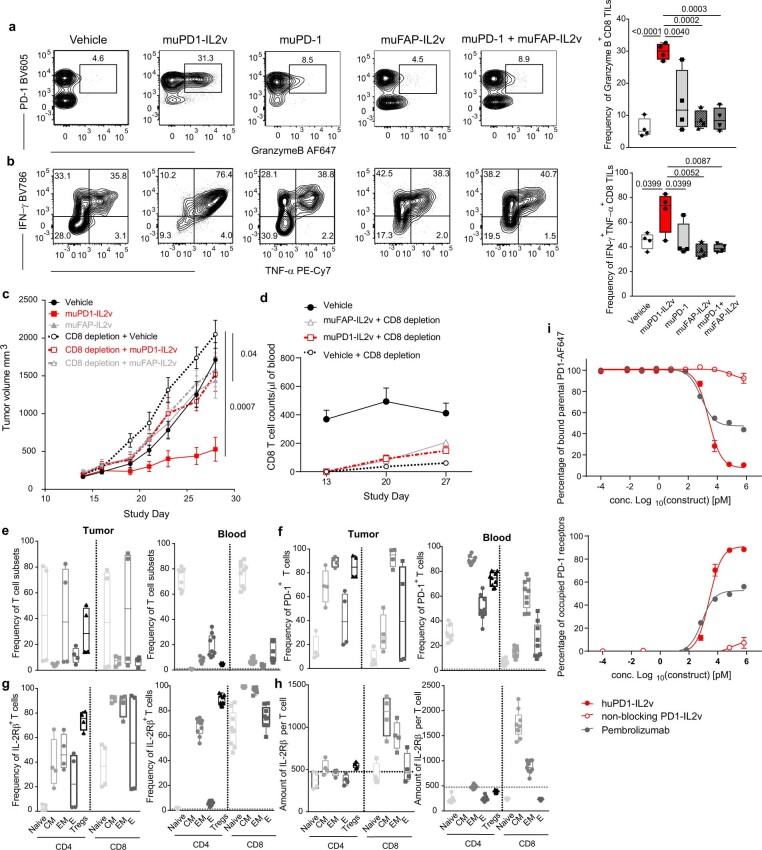
CD8 T cells acquire a polyfunctional effector profile upon muPD1-IL2v and are critical for its efficacy. Frequency and amount of PD-1 and IL-2Rβ per T cell in the tumor and blood of huPD1-transgenic mice. **a**-**b**. Left, representative FACS contour plot of PD-1^+^ CD8 TILs secreting granzyme B, IFN-γ and TNF-α across different treatment groups; right, frequency of PD-1^+^ granzyme B^+^ and IFN-γ^+^ TNF-α^+^ CD8 TILs (n = 4 mice per group per experiment from 3 independent experiments, box plots representing median, minimum/maximum and individual points). Statistical comparisons were performed using one-way ANOVA with Tukey’s multiple comparison test. **c**. Tumor growth inhibition and **d**. CD8 T cell count in blood of syngeneic mice bearing subcutaneous Panc02-H7-Fluc tumors with or without CD8 depletion before the start of the indicated treatments (n = 11 mice per treatment group, mean ± SEM). **e**–**h**. Frequencies of receptor positive T cells and quantification of PD-1 receptors and IL-2Rβ on T cells isolated from tumors and blood of untreated human PD-1 transgenic mice bearing Panc02-H7-Fluc (n = 4 and n = 9 mice respectively, box plots representing median, minimum/maximum and individual points). **i**. (Top) Percentage of directly conjugated Alexa Fluor-647 parental anti-PD-1 bound to 3 days activated CD4 T cells previously exposed to increasing concentrations of either PD1-IL2v, pembrolizumab or non-blocking PD1-IL2v; (bottom) percentage of PD-1 receptors occupied by either PD1-IL2v, pembrolizumab or non-blocking PD1-IL2v and therefore unavailable for binding of the directly conjugated Alexa Fluor-647 parental anti-PD1 (n = 2 healthy donors from 2 independent experiments, mean ± SEM). Source Data

Further characterization of CD8^+^ TILs across the various treatment groups highlighted dissimilarities in their differentiation stage. While the anti-PD-1 therapy enriched terminally differentiated TILs, muPD1-IL2v generated and expanded effector memory TILs (Fig. 3f). Conversely, muFAP-IL2v expanded naive TILs, and its combination with muPD-1 retained the features of both molecules by enriching both naive and terminally differentiated TILs. CD8^+^ TILs induced by muPD1-IL2v were polyfunctional and co-expressed significantly higher levels of granzyme B, IFNγ and tumour necrosis factor-α (TNFα) than CD8^+^ TILs isolated from mice from the other treatment groups (Extended Data Fig. 7a,b). In blood, the effect of muPD1-IL2v treatment was comparable to that of muFAP-IL2v (Fig. 3f), highlighting the importance of higher PD-1 expression, such as in TILs versus peripheral blood T cells, for the differentiated effects of muPD1-IL2v treatment over muFAP-IL2v.

To verify that CD8^+^ T cells are critical for the efficacy associated with muPD1-IL2v therapy, we depleted CD8^+^ cells 1 week before administering either muPD1-IL2v or muFAP-IL2v and monitored the number of CD8^+^ T cells in the blood over time. The effect of CD8^+^ T cell depletion in the muFAP-IL2v-treated group was not appreciable owing to the lack of efficacy of muFAP-IL2v in this tumour model (Extended Data Fig. 7c). However, depletion of CD8^+^ T cells prevented muPD1-IL2v from achieving tumour growth inhibition when compared with muPD1-IL2v-treated mice that were not depleted of CD8^+^ T cells (Extended Data Fig. 7c,d), demonstrating that CD8^+^ T cells are indeed required for the efficacy observed under muPD1-IL2v therapy.

## CD8^+^ TILs are preferentially targeted by PD1-IL2v

To better understand the tumour tropism of PD1-IL2v, we isolated leukocytes from the blood and tumours of human PD-1-transgenic mice bearing subcutaneous Panc02-H7-Fluc tumours. We then measured, ex vivo, the frequencies of T cells expressing PD-1 and IL-2Rβ and quantified on these cells the numbers of both receptors per T cell. While the frequencies of T cells expressing PD-1 on their surface were relatively comparable  in peripheral blood and tumours (Extended Data Fig. 7e,f), we found in tumours an effector memory population of CD8^+^ T cells expressing much higher levels of PD-1, approximately 15,000 PD-1 receptors per T cell (Fig. 3g). Interestingly, in blood, the corresponding T cell subset expressed ~700 PD-1 receptors per T cell, similarly to other T cell subsets including effector memory CD4^+^ T cells and T_reg_ cells (Fig. 3g). By contrast, both IL-2Rβ^+^ frequencies and receptor numbers per T cell were similar in the tumour and peripheral blood, with a higher number of receptors on the surface of central and effector memory CD8^+^ T cells in both compartments (Extended Data Fig. 7g,h).

We then treated human PD-1-transgenic mice, implanted subcutaneously with Panc02-H7-Fluc tumours, with either 0.5 mg kg^–1^ PD1-IL2v, comprising the anti-human PD-1 antibody binder fused to IL-2v, or high-dose IL-2 (aldesleukin) as single agents or in combination with pembrolizumab. By the end of the experiment, 7 of 12 mice (58%) receiving PD1-IL2v had a tumour smaller than 100 mm^3^, while 11 of 12 had a tumour smaller than 500 mm^3^. By contrast, only one of seven mice (14%) treated with high-dose aldesleukin in combination with 10 mg kg^–1^ pembrolizumab had a tumour smaller than 100 mm^3^ (Fig. 3h). Although the PD-1-binding domain in PD1-IL2v competes for PD-1 binding with pembrolizumab, combination of PD1-IL2v with pembrolizumab did not impair PD1-IL2v efficacy. This can be explained by the superior functional affinity of PD1-IL2v, resulting from an approximately fourfold-higher monovalent PD-1 affinity and from simultaneous binding in *cis* to IL-2R, allowing PD1-IL2v to displace pembrolizumab even at saturating concentrations of the latter (Extended Data Fig. 7i). In line with this, combination of PD1-IL2v with pembrolizumab did not provide any additional benefit in comparison to PD1-IL2v monotherapy, as 8 of 12 treated animals (66%) had a tumour smaller than 100 mm^3^ at experiment termination (Fig. 3h). These data confirm that PD1-IL2v as monotherapy is more efficacious than the combination of pembrolizumab with high-dose aldesleukin, that PD1-IL2v does not require additional PD-1 blockade to increase its efficacy in this tumour model at the tested doses and that CD8^+^ TILs express roughly 20-fold more PD-1 receptors per T cell than CD8^+^ T cells in the blood, supporting the rationale for a tumour-preferential effect of PD1-IL2v.

## muPD1-IL2v yields better effector CD8^+^ TILs

**Fig. 4:**
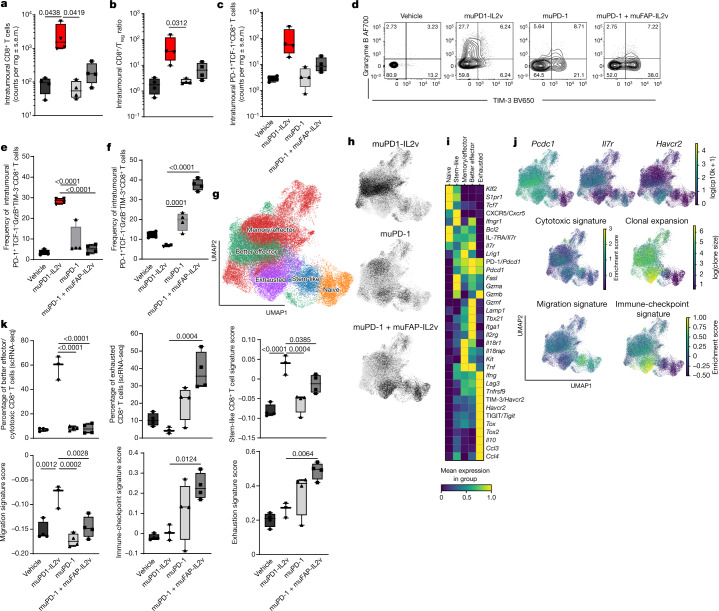
muPD1-IL2v expands and differentiates PD-1^+^TCF-1^+^ stem-like resource CD8^+^ TILs into a new population of better effector CD8^+^ TILs. Immuno-pharmacodynamic study on the effect of the different therapies, given twice, on the abundance, phenotype, effector function and molecular signature of intratumoural CD8^+^ T cells obtained from syngeneic mice bearing subcutaneous Panc02-H7-Fluc tumours. **a**, Number of intratumoural CD8^+^ T cells. **b**, CD8^+^ T cell to T_reg_ ratio within the tumour. **c**, Number of stem-like (PD-1^+^TCF-1^+^) CD8^+^ T cells. In **a**–**c**, *n* = 4 (box plots represent the median, minimum/maximum and individual points; treatment groups appear in the same order in each panel). **d**, Representative contour plots depicting granzyme B and TIM-3 expression on PD-1^+^TCF-1^−^CD8^+^ TILs from tumour single-cell suspensions acquired by flow cytometry 3 d after administration of the second dose of the treatment as indicated. **e**,**f**, Frequencies of granzyme B^+^TIM-3^−^ (**e**) and granzyme B^−^TIM-3^+^ (**f**) intratumoural PD-1^+^TCF-1^−^CD8^+^ T cells (*n* = 4; box plots represent the median, minimum/maximum and individual points). **g**,**h**, Two-dimensional (2D) UMAP visualization of CD8^+^ TILs coloured according to subset (**g**) and specific treatment effect (**h**). **i**, Average relative expression of selected genes (RNA and/or protein level) across the distinct T cell subsets within the CD8^+^ TILs depicted in **g** and **h**. **j**, Expression of selected markers, signature scores and TCR clonal expansion among CD8^+^ TILs using a 2D UMAP visualization as in **g** and **h**. log(cp10k), natural logarithm of counts per 10,000; log(clone size), natural logarithm of clone size. **k**, Percentage of better effectors and exhausted CD8^+^ T cells relative to all CD8^+^ TILs across the different treatments and average signature enrichment scores among effector CD8^+^ T cells per treatment group and individual animal (3–4 mice per group; box plots represent the median, minimum/maximum and individual points). In **a** and **k**, *n* = 3–4 mice per group per experiment, >3 independent experiments; statistical comparisons were performed using one-way or two-way ANOVA with Dunnett’s multiple-comparisons test. Source Data

Immuno-pharmacodynamic analysis of subcutaneous Panc02-H7-Fluc tumours from syngeneic mice showed a progressive and large expansion of CD8^+^ T cells within the tumours of animals treated with muPD1-IL2v and a beneficial CD8^+^ T cell/T_reg_ ratio after two single doses of muPD1-IL2v (Fig. 4a,b). This observation is consistent with the in vitro findings of preferential targeting and activity of PD1-IL2v on effector T cells rather than T_reg_ cells.

On the basis of PD-1 and TCF-1 expression, as reported by Hashimoto et al.^45^ and by previous publications^1,2,46^, we identified antigen-experienced stem-like T cells as PD-1^+^TCF-1^+^ whereas their progeny were identified as PD-1^+^TCF-1^low^^/^^−^ (Fig. 4c–f). We then further discriminated the functionality and degree of exhaustion of more mature PD-1^+^CD8^+^ T cells on the basis of their expression levels of granzyme B and TIM-3 (Fig. 4d). Interestingly, muPD1-IL2v drove the expansion of stem-like CD8^+^ TILs (Fig. 4c) and significantly increased the frequency of a granzyme B^+^TIM-3^−^ population within PD-1^+^TCF-1^low/−^CD8^+^ TILs (Fig. 4d,e), here named ‘better effectors’, to underscore their highly functional effector profile and lower degree of exhaustion. Conversely, muPD-1 monotherapy and combination of muPD-1 with muFAP-IL2v significantly increased the frequency of granzyme B^−^TIM-3^+^ cells within PD-1^+^TCF-1^low/−^CD8^+^ TILs (Fig. 4d,f), showing low functionality and a higher degree of exhaustion.

**Extended Data Fig. 8:**
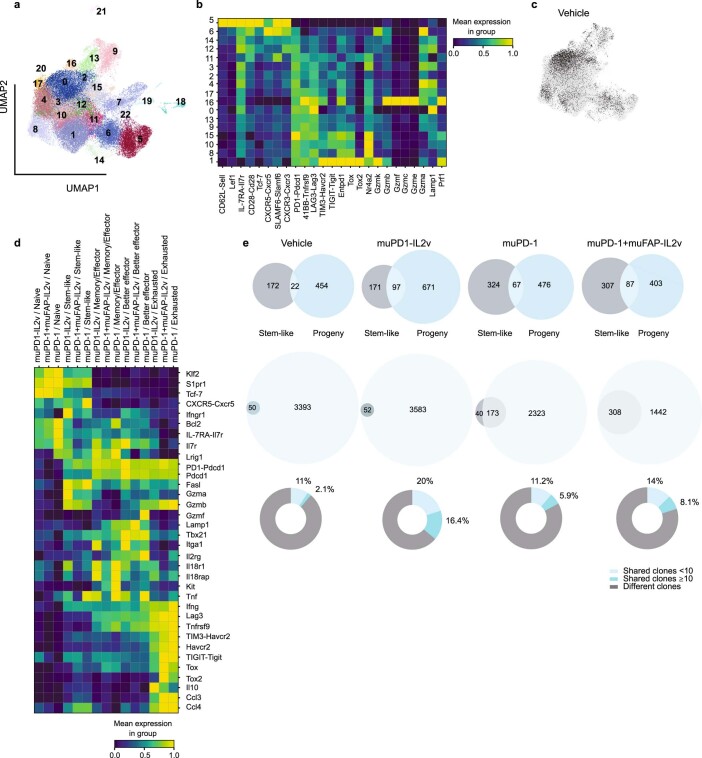
MuPD1-IL2v expands PD-1^+^ TCF-1^+^ stem-like resource CD8^+^ TILs and their progeny and enhances their cytotoxicity. **a**. Joint 2D UMAP visualization of all cells across all treatments and individual mice colored according to Leiden clusters. **b**. Selected relative average marker expression at RNA and protein (in capitals) level identifies the majority of cells as CD8 T cells as expected (21, 18, 19 exception, 7 myeloid/T cell doublet, 22 Th17, 20 regulatory T cell specific expression within CD8^+^) (left). Among CD8 T cell clusters, 5 shows naïve specific, 6 stem-like, 11 and 1 exhausted-like, 3, 4, 12, 14, 16, 17 better effector and the rest more broadly memory/effector-like (right). **c**. 2D UMAP visualization of the CD8 T cell distribution in the vehicle (control) group. **d**. Relative marker expression stratified per cell type and treatment for 3 main treatment groups: muPD1-IL2v, muPD-1 monotherapy and combination with FAP-IL2v. **e**. (Top) Number of distinct TCR clones present within stem-like T cells, within their progeny (all CD8 effector/exhausted subsets) and the ones shared between the two per treatment group; (middle) number of cells (normalized to 10.000) with a high clonotype expansion (≥10), per treatment group; (bottom) percentages of TCR clones in the stem-like T cells that are shared with their progeny with low clonotype (<10) or high clonotype expansion (≥10). (a-e). n = 3-4 mice per treatment group.

Single-cell RNA-seq (scRNA-seq) and feature barcoding of CD8^+^ TILs obtained from the same in vivo experiment showed a unique gene expression signature following administration of muPD1-IL2v versus muPD-1 as monotherapy and in combination with muFAP-IL2v (Fig. 4g,h and Extended Data Fig. 8a–d). muPD1-IL2v drove the enrichment of a new CD8^+^ T cell population of better effectors (clusters 3, 4, 12, 14, 16 and 17), which was missing or under-represented in the other treatment groups (Fig. 4g,h and Extended Data Fig. 8a–d). muPD-1 therapy, and muPD-1 in combination with muFAP-IL2v even more so, drove the expansion of terminally differentiated/exhausted TILs (clusters 1 and 11) (Fig. 4g,h and Extended Data Fig. 8a–d). Similarly to the findings previously reported for chronic LCMV settings, we observed that muPD1-IL2v induced in the population of better effectors gene expression of receptors for pro-inflammatory cytokines, such as *Il2rg*, *Il18r1*, *Il18rap* and *Ifngr*, as well as those for homeostatic proliferation and memory formation, including *Il7r* (Fig. 4i,j and Extended Data Fig. 8d). In addition, this population of CD8^+^ TILs also expressed high levels of transcripts for *Pdcd1* (PD-1) and intermediate levels of *Lag3* while expressing low levels of *Havcr2* (TIM-3), *Tigit* and *Tox*, in line with a non-exhausted profile (Fig. 4i,j and Extended Data Fig. 8d). The presence of expression of *Ifitm1* and *Tbx21* together with the polyfunctional effector signature of *Tnf* and *Ifng*, as well as the cytotoxic effector signature of the *Gzm* gene family and *Lamp1*, supports the finding that muPD1-IL2v is able to promote durable, productive and protective immune memory. Conversely, terminally differentiated CD8^+^ TILs (clusters 1 and 11) generated following treatment with muPD-1 as monotherapy or in combination with muFAP-IL2v, expressed high levels of *Havcr2*, *Lag3*, *Tigit*, *Tox* and *Il10*, typical of exhausted T cells^47^ (Fig. 4i,j and Extended Data Fig. 8d).

These differences were both qualitative and quantitative, as illustrated by the significantly higher frequency of better effectors in response to muPD1-IL2v treatment, in contrast to the significantly higher frequency of exhausted CD8^+^ TILs elicited by muPD-1 alone and in combination with muFAP-IL2v (Fig. 4k). The CD8^+^ TILs generated by muPD1-IL2v possessed significantly higher stem-like and migration signature scores, indicating that they retain some of the transcriptional characteristics of stem-like PD-1^+^TCF-1^+^CD8^+^ TILs (Fig. 4k). By contrast, the immune-checkpoint and exhaustion signature scores were significantly higher in CD8^+^ TILs generated by the combination treatment of muPD-1 and muFAP-IL2v (Fig. 4k).

Single-cell TCR-seq showed that both better effectors, generated following muPD1-IL2v treatment, and the exhausted T cells that arise following treatment with muPD-1 as monotherapy or in combination with muFAP-IL2v consisted of clonally expanded CD8^+^ TILs (Fig. 4j), a bona fide indicator of tumour specificity and productive immune response^48,49^. We then assessed the total number of clones present in the stem-like CD8^+^ TILs and their progeny of effector cells across the different treatment groups, regardless of their functional phenotype. In the muPD1-IL2v-treated group, we found a high number of clones among the effector cells (768 clones), 97 of which were shared with stem-like CD8^+^ TILs (Extended Data Fig. 8e, top). We also observed the highest number of cells with highly (>10) expanded clones in the effector cells generated by muPD1-IL2v treatment, corresponding to a more than twofold difference compared with muPD-1 as monotherapy or in combination with muFAP-IL2v (Extended Data Fig. 8e, middle). In addition, the muPD1-IL2v-treated group had the highest fraction of clones shared between the effector progeny and stem-like CD8^+^ TILs (46.4%) when compared with muPD-1 as monotherapy (17.1%) or in combination with muFAP-IL2v (22.1%). Of the 46.4% of shared clones, 16.4% were highly expanded clonotypes, as opposed to only 5.9% and 8.1% of the shared clones with muPD-1 as monotherapy and in combination with muFAP-IL2v, respectively (Extended Data Fig. 8e, bottom).

These results when taken together demonstrate that muPD1-IL2v therapy acts on stem-like CD8^+^ TILs and leads to the expansion of a unique CD8^+^ T cell population of better effectors with a transcriptional signature containing the hallmark of productive and protective immune memory. In addition, better effector CD8^+^ TILs have a high overlap in clonotypes with stem-like CD8^+^ TILs, indicating their developmental path, and the highest number of expanded clones, suggestive of their tumour specificity.

## Better effector CD8^+^ TILs provide survival benefit

**Fig. 5:**
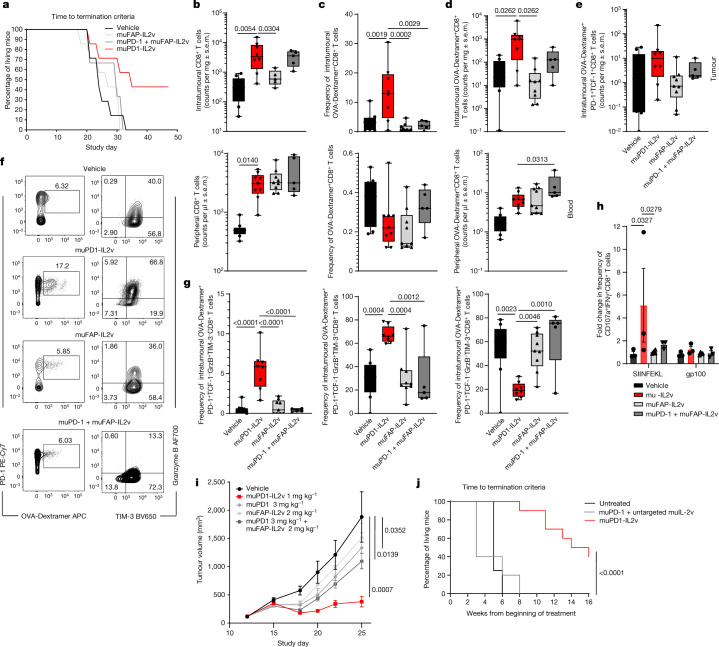
muPD1-IL2v provides survival benefit and control of tumour growth in mice with subcutaneous B16-F10-OVA tumours by expanding cytotoxic OVA-specific better effector CD8^+^ TILs. In vivo efficacy study and immuno-pharmacodynamic study on the effect of the different therapies, given twice, on the number, phenotype and effector function of intratumoural and peripheral CD8^+^ T cells in syngeneic mice bearing subcutaneous B16-F10-OVA tumours. **a**–**d**, Survival (**a**), counts of total CD8^+^ T cells (**b**), and frequency (**c**) and count (**d**) of OVA-specific CD8^+^ T cells in the tumour and blood of syngeneic mice bearing subcutaneous B16-F10-OVA tumours receiving the indicated treatment (*n* = 5–8; box plots represent the median, minimum/maximum and individual points). **e**, Treatment effect on counts of intratumoural OVA-specific PD-1^+^TCF-1^+^ stem-like CD8^+^ T cells (*n* = 5–8; box plots represent the median, minimum/maximum and individual points). **f**, Representative contour plots depicting PD-1^+^OVA-Dextramer^+^ double-positive CD8^+^ TILs and their granzyme B and TIM-3 expression 3 d after administration of the second dose of treatment as indicated. **g**, Treatment effect on frequencies of granzyme B^+^TIM-3^−^ (left), granzyme B^+^TIM-3^+^ (middle) and granzyme B^−^TIM-3^+^ (right) intratumoural OVA-specific CD8^+^ T cells (*n* = 5–8; box plots represent the median, minimum/maximum and individual points). **h**, Fold increase in the frequency of CD107a^+^IFNγ^+^CD8^+^ TILs from the different treatment groups following re-stimulation for 5 h with either SIINFEKL or gp100 peptide (*n* = 3; box plots represent the median, minimum/maximum and individual points). **i**, Tumour growth inhibition in the MCA-205 sarcoma model in syngeneic mice (*n* = 9 mice per treatment group; mean ± s.e.m.). **j**, Survival graph of tumour-bearing RIP-Tag5 mice either left untreated or subjected to treatment as indicated. Tumour progression was monitored by ultrasound imaging for 16 weeks. Two mice in the muPD1-IL2v group developed hyperglycaemia due to complete islet tumour regression and had to be killed before the predefined study end of 16 weeks. These mice were still counted as complete responders. Numbers of mice were as follows: untreated, *n* = 4; muPD1 + untargeted muIL-2v, *n* = 5; muPD1-IL2v, *n* = 10. Statistical analysis was performed by log-rank Mantel–Cox test: muPD1-IL2v versus muPD1 + untargeted muIL-2v, *P* < 0.0001. In **a**–**h**, *n* = 5–8 mice per treatment group, 2 independent experiments; statistical comparisons were performed using one-way or two-way ANOVA with Dunnett’s multiple-comparisons test. Source Data

**Extended Data Fig. 9:**
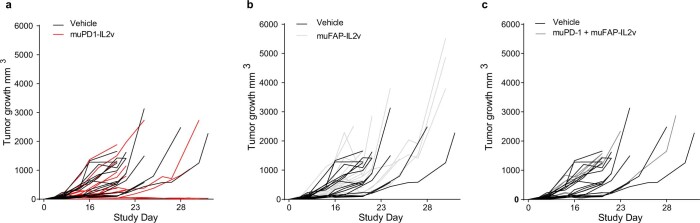
Tumor growth inhibition and survival curves in B16-F10-OVA tumor mouse model. **a**. Tumor growth kinetics upon muPD1-IL2v, **b**. muFAP-IL2v and **c**. muPD-1 in combination with muFAP-IL2v. (a-c) n = 5-8 mice per treatment group, 2 independent experiments. Source Data

We then assessed muPD1-IL2v in an in vivo efficacy study in C57BL/6 mice implanted subcutaneously with the B16-F10-OVA syngeneic cell line. Mice were treated once a week for 2 weeks with muPD1-IL2v (0.5 mg kg^–1^) or muFAP-IL2v (1.5 or 3 mg kg^–1^) as monotherapy or in combination with muPD-1 (10 mg kg^–1^). muPD1-IL2v provided longer survival benefit to 50% of the treated animals, and the tumours were eradicated in 20% of the total mice (Fig. 5a and Extended Data Fig. 9a). None of the mice receiving muFAP-IL2v as monotherapy or in combination with muPD-1 survived until the end of the experiment or showed controlled tumour growth (Fig. 5a and Extended Data Fig. 9b,c). muPD1-IL2v significantly increased the total number of intratumoural CD8^+^ T cells (Fig. 5b) but, more notably, significantly expanded the frequency and total count of ovalbumin (OVA)-specific CD8^+^ TILs when compared with muFAP-IL2v as monotherapy or in combination with muPD-1 (Fig. 5c,d, top). Interestingly, the combination of muPD-1 with muFAP-IL2v significantly expanded the frequency and total count of OVA-specific CD8^+^ T cells in the blood but not in the tumour (Fig. 5c,d, bottom).

In addition, muPD1-IL2v significantly expanded intratumoural OVA-specific PD-1^+^TCF-1^+^ stem-like T cells in comparison to the other treatments (Fig. 5e).

Phenotypic and functional characterization of the PD-1^+^TCF-1^low/−^ OVA-specific CD8^+^ TILs showed that muPD1-IL2v induced a significant expansion of the frequencies of granzyme B^+^TIM-3^−^ and granzyme B^+^TIM-3^+^ populations, whereas muFAP-IL2v and its combination with muPD-1 increased the frequency of the granzyme B^−^TIM-3^+^ population (Fig. 5f,g). The observed differences in the TIM-3 expression profile following treatment with muPD1-IL2v in the B16-F10-OVA mouse model and the subcutaneous Panc02-H7-Fluc mouse model might reflect the different immunogenicity of the two types of tumours and the relative difference in avidity of the T cell receptors (TCRs) for tumour antigens^50^.

CD8^+^ TILs isolated from mice treated with muPD1-IL2v showed the ability to mount a fast antigen-specific effector response when re-stimulated for 5 h with an OVA peptide (Fig. 5h).

We further explored two additional mouse models for responsiveness towards muPD1-IL2v: MCA-205 sarcoma, which is partially sensitive to PD-1 blockade, and RIP-Tag5, a spontaneous pancreatic neuroendocrine tumour model that is unresponsive to anti-PD-1 therapy. In the MCA-205 tumour model, muPD1-IL2v provided superior tumour growth inhibition to the treated mice in comparison to muPD-1 and muFAP-IL2v as monotherapy and in combination (Fig. 5i). Similarly, treatment of RIP-Tag5 mice with muPD1-IL2v resulted in increased survival benefit compared with the combination therapy of muPD-1 plus untargeted muIL-2v (Fig. 5j). Of interest, two complete responders from the RIP-Tag5 study had to be killed during the study because of hyperglycaemia as a consequence of the potent anti-tumour immune response elicited by muPD1-IL2v that evidently resulted in organ-specific autoimmunity.

## Discussion

Stem-like, antigen-experienced PD-1^+^TCF-1^+^CD8^+^ T cells, or ‘stem-like T cells’, have emerged as important determinants of the immune response in chronic infections and cancer, with the size of their tumour-associated pool critical to the success of cancer immunotherapies blocking PD-1 or PD-L1 (refs. ^1–3^). In this study and in the accompanying article^45^, we show that PD-1 inhibition alone acts on stem-like T cells to expand a population of transitory effector cells but eventually leads to the accumulation of exhausted T cells. By contrast, adding IL-2 triggers an alternative differentiation path from stem-like cells to a distinct subset of highly proliferative and cytotoxic CD8^+^ T cells, or ‘better effectors’. We found that IL-2 binding to the non-signalling component of its receptor, CD25, is required for this process. However, CD25 binding can also contribute to unwanted effects of systemic IL-2 therapy, as occurs with high-dose aldesleukin therapy, and this has led to the development of various IL-2Rβγ-biased agonists with reduced or abolished CD25 binding, currently in clinical trials^8–10,51^.

To address the challenge of systemic IL-2 therapy without losing the beneficial properties of IL-2 on stem-like T cells, we substituted binding to CD25 by targeting PD-1 with a blocking, high-affinity anti-PD-1 antibody fused to an IL-2 variant devoid of CD25 binding. This allowed specific delivery of enhanced *cis* IL-2R agonism to PD-1^+^ antigen-experienced T cells, such as virus-specific and tumour-reactive T cells. We found that binding in *cis* of PD1-IL2v to PD-1 and IL-2Rβγ on the cell surface of the same T cell allows IL-2v to differentiate stem-like CD8^+^ T cells into better effectors in the absence of CD25 binding in both chronic infection and cancer models.

In the chronic LCMV infection model, we showed that these better effectors generated by PD1-IL2v from stem-like T cells have a transcriptional profile closely resembling that of the effector CD8^+^ T cells described in the accompanying article^45^, generated following treatment with a combination of PD-1 inhibition and IL-2, with normal CD25 binding. They share characteristics of effectors generated during an acute infection, having lower levels of inhibitory receptors (for example, TIM-3) and transcription factors associated with T cell exhaustion (for example, Tox) and higher levels of IFNγ and IL-2 production. Better effectors also have higher levels of effector molecules (for example, granzyme family members) and inflammatory cytokine receptors (for example, IL-18R). In addition, expression of genes encoding factors associated with memory (for example, IL-7R) and migration (for example, CXCR3) was enhanced in this effector subset. Expansion of these highly proliferative and cytotoxic CD8^+^ T cells with a distinct transcriptional profile was associated with superior anti-viral and anti-tumour responses. By contrast, antibodies blocking PD-1 and PD-L1 alone, or in combination with clinically relevant doses of IL-2 molecules devoid of CD25 binding and not targeted to PD-1, could not expand better effectors and instead induced exhausted T cells, leading to inferior treatment efficacy.

In the last decade, immune-checkpoint inhibitors targeting the PD-1–PD-L1 pathway have revolutionized the standard of care for several types of tumours by acting on stem-like T cells and expanding tumour-specific transitory effector cells. The findings described here provide a basis for the development of a new generation of PD-1-*cis*-targeted IL-2Rβγ agonists, preferentially targeting antigen-specific stem-like T cells but expanding an alternative population of better effector cells with enhanced therapeutic potential for the treatment of cancer and chronic infections.

## Methods

### Human PBMC isolation

Blood samples from healthy volunteers were obtained through a blood donation centre (Zurich, Switzerland) with approval of the Cantonal Ethics Committee (Zurich). PBMCs were isolated from the blood of different healthy donors using density gradient centrifugation with Histopaque-1077 (Sigma). All cells were cultured in RPMI-1640 (Gibco) supplemented with 10% heat-inactivated FBS (Gibco), GlutaMAX (Gibco) and 1% penicillin-streptomycin (100×; Gibco).

### Human and mouse CD4^+^ T cell isolation and in vitro activation

Human CD4^+^ T cells were sorted by using a CD4^+^ selection Miltenyi bead system following the manufacturer’s instructions. Thereafter, the cells were labelled with CFSE (5 μM, 5 min at room temperature; eBioscience) or CTV (5 μM, 5 min at room temperature; Thermo Scientific) to measure cell proliferation.

CD4^+^ T cells were seeded into a plate precoated with anti-CD3 antibody (1 μg ml^–1^; clone OKT3, BioLegend; overnight, 4 °C) with addition of soluble anti-CD28 antibody (1 μg ml^–1^; clone CD28.2, BioLegend). Cells were cultured for 3 d to induce activation and upregulation of the PD-1 receptor on the surface of CD4^+^ T cells.

Spleens of C57BL/6 mice were homogenized to a single-cell suspension by mashing the spleen through a 100-µm cell strainer, and erythrocytes were lysed with ACK (ammonium chloride–potassium) lysis buffer for 5 min at 4 °C. CD4^+^ T cells were sorted with a CD4 negative-selection Miltenyi bead system following the manufacturer’s instructions. CD4^+^ T cells were seeded into a plate precoated with anti-CD3/anti-CD28 antibodies (5 μg ml^–1^ for clone 145-2C11 (BioLegend) and 5 μg ml^–1^ for clone 37.51 (BioLegend)) and activated for 3 d.

### IL-2R signalling (STAT5-P) in PD-1^+^ and PD-1-blocked CD4^+^ T cells

After 3 d of in vitro activation, cells were collected and washed multiple times to remove endogenous IL-2. A portion of the CFSE-labelled T cells were exposed to 10 µg ml^–1^ of parental anti-PD-1 antibody to block the PD-1 epitope for 30 min at room temperature and, thereafter, unbound antibody was washed away.

To assess IL-2R signalling (STAT5-P) on human T cells following treatment, both anti-PD-1-pretreated and untreated cells were exposed to increasing concentrations of PD1-IL2v, FAP-IL2v or FAP-IL2 superkine analogue^24^ for 12 min at 37 °C. To investigate the *cis*/*trans* binding of PD1-IL2v, anti-PD-1-pretreated or untreated CFSE-labelled cells were co-cultured 1:1 with untreated CTV-labelled cells. Cells were then exposed for 12 min at 37 °C to 0.1 μg ml^–1^ (630 pM) of the treatment fusion proteins.

For the mouse ex vivo experiment, the cells were treated with increasing doses of muPD1-IL2v or muFAP-IL2v for 30 min at 37 °C.

Directly after treatment, cells were fixed with Phosphoflow Fix Buffer I (BD) and incubated for 30 min at 37 °C. Cells were then permeabilized overnight at −80 °C with Phosphoflow PermBuffer III (BD) before being stained for 30 min at 4 °C with anti-STAT5-P–AF647 antibody (clone 47/pY694, BD Biosciences; 1:20).

### Binding competition on T_reg_ and T_conv_ cells and T_reg_ suppression assays

CD4^+^CD25^+^CD127^low^ T_reg_ cells were isolated from human peripheral blood with the two-step Regulatory T Cell Isolation kit (Miltenyi). In parallel, CD4^+^CD25^−^ T_conv_ cells were isolated by collecting the negative fraction from selection of CD25^+^ cells (Miltenyi) followed by enrichment of CD4^+^ cells (Miltenyi). T_conv_ cells were labelled with CFSE and T_reg_ cells were labelled with CTV to track the proliferation of both populations.

For PD-1 and IL-2Rβ receptor quantification and PD1-IL2v binding competition, T_reg_ and T_conv_ cells were co-cultured at a 1:1 ratio in a plate precoated with anti-CD3 antibody (1 μg ml^–1^; clone OKT3, BioLegend) with soluble anti-CD28 antibody (1 μg ml^–1^; clone CD28.2, BioLegend). After 3 d of stimulation, a competitive binding assay was conducted with 1 μg ml^–1^ (6.3 nM) of either parental anti-PD-1 antibody or PD1-IL2v, which were both directly labelled with AF647. Cells were incubated with the directly coupled antibodies for 30 min at 4 °C and fixed with CellFIX (BD).

In the T_reg_ suppression assay, the rescue of T_conv_ granzyme B production following treatment with PD1-IL2v was measured after co-culturing T_conv_ cells together with T_reg_ cells at a 2:1 ratio for 5 d, in the presence or absence of treatment. Irradiated (40 Gy) feeders from an unrelated donor were used to elicit allospecific stimulation. Suppression by T_reg_ cells was calculated with the following formula:

$$ \% \,{\rm{Cytokine}}\,{\rm{suppression}}=100-(( \% \,{{\rm{cytokine}}}_{{\rm{Tconv}}+{\rm{Treg}}\pm {\rm{therapy}}})/( \% \,{{\rm{cytokine}}}_{{\rm{Tconv}}}))\times 100$$

where % cytokine_Tconv + Treg ± therapy_ is the level of cytokine secreted by T_conv_ cells in the presence of T_reg_ cells ± treatment and % cytokine_(Tconv)_ is the level of cytokine secreted by T_conv_ cells in the absence of T_reg_ cells and without treatment.

### GM-CSF, granzyme B and IFNγ secretion by CD4^+^ T cells

Sorted and CTV-labelled human polyclonal CD4^+^ T cells were activated with soluble anti-CD3 antibody (1 μg ml^–1^) in the presence of irradiated (40 Gy) feeder cells from the same donor at a 1:1 ratio and increasing concentrations of treatment antibodies or aldesleukin (Proleukin, Novartis). After 5 d, GM-CSF secretion was measured by ELISA (BioLegend) following the manufacturer’s instructions. For intracellular flow cytometry staining, accumulation of cytokines in the Golgi complex was induced by re-stimulating cells with ionomycin (500 ng ml^–1^) and phorbol 12-myristate 13-acetate (PMA; 50 ng ml^–1^) together with protein transport inhibitors (1 μl GolgiPlug and GolgiStop, BD) for 5 h before staining.

### Binding competition

CD4^+^ T cells activated for 3 d were exposed to increasing equimolar concentrations of PD1-IL2v, pembrolizumab or non-blocking PD1-IL2v for 30 min at 4 °C. After a washing step, cells were incubated for an additional 30 min at 4 °C with saturating concentrations (10 μg ml^–1^) of a parental anti-PD-1 antibody conjugated directly to AF647 used to generate PD1-IL2v. Cells were fixed with CellFIX (BD) after an additional wash.

### Flow cytometry staining for cytokine detection and receptor quantification

Cells were stained in PBS with antibodies to cell-surface markers for 30 min at 4 °C and for live/dead status (with either Aqua Dead Cell Stain (Invitrogen) during the last 10 min of incubation or Fixable Viability Dye eFluor 780 (eBioscience) for 30 min at 4 °C). For intracellular staining, cells were permeabilized with FACS permeabilization buffer (Fixation/Permeabilization, BD Biosciences or Foxp3 Transcription Factor Fixation kit, eBioscience) and then incubated with antibodies specific for cytokines for 60 min at 4 °C. The following antibody mixes were used: (1) antibodies to human proteins: anti-PD-1 (2.5 μg ml^–1^; clone EH12.2H7, BioLegend), anti-IL-2Rβ (2.5 μg ml^–1^; clone TU27, BioLegend), isotype control (2.5 μg ml^–1^; clone MOPC-21, BioLegend), anti-CD4 (1:50; clone RPA-T4, eBioscience), anti-GM-CSF (1:100; clone BVD2-21C11, BioLegend), anti-granzyme B (1:100; GB11, BD Biosciences), anti-IFNγ (1:100; clone 4S.B3, eBioscience); (2) antibodies to mouse proteins: anti-PD-1 (2.5 μg ml^–1^; clone 29F.1A12 (for syngeneic mice) or clone EH12.2H7 (for human PD-1-transgenic mice), BioLegend), anti-IL-2Rβ (2.5 μg ml^–1^; clone 5H4, BioLegend), isotype control (2.5 μg ml^–1^; clone RTK2758, BioLegend), anti-TCRβ (1:200; clone H57–597, BioLegend), anti-CD8 (1:200; clone 53-6.7, BioLegend), anti-CD4 (1:100; clone GK1.5, BioLegend), anti-CD45 (1:300; clone 30-F11, BioLegend), anti-CD62L (1:200; clone MEL-14, BioLegend), anti-CD44 (1:200; clone IM7, BD), anti-FoxP3 (1:100; clone 150D, BioLegend).

The number of PD-1 and IL-2Rβ receptors was quantified on the cell surface of PBMCs and TILs of human PD-1-transgenic mice bearing Panc02-H7-Fluc tumours and on activated human T_reg_ and T_conv_ cells with the PE Fluorescence Quantitation kit (BD) following the manufacturer’s instructions. PE-labelled monoclonal antibodies (2.5 µg ml^–1^) were used to quantify the receptor of interest on gated populations of interest. Cells and PE Quantibrite beads were fixed following the same protocol, and fluorescence data were acquired while using the same settings. The number of receptors was quantified following the kit’s instructions.

Ex vivo binding of PD1-IL2v, FAP-IL2v and FAP-superkine analogue was performed by incubating 630 pM of the constructs for 30 min on PBMCs from healthy donors. After a washing step, cells were incubated for an additional 30 min at 4 °C with a PE-labelled antibody recognizing the PGLALA mutation in the Fc portion of the primary antibodies together with a panel of antibodies to characterize the phenotype of the immune-cytokine targeted cells: anti-CD3 (1:100; clone OKT3, BioLegend), anti-CD4 (1:100; clone OKT4, BD Biosciences), anti-CD8 (1:100; clone RPA-T8, BD Biosciences), anti-TIM-3 (1:20; clone F38-2E2, BioLegend), anti-CD218a (1:100; clone H44, BioLegend), anti-CD56 (1:20; clone NCAM16.2, BD Biosciences), anti-TCF-1 (1:100; C63D9, Cell Signaling Technology), anti-FOXP3 (1:50; clone 206D, BioLegend) and anti-PD-1 (1:100; clone D4W2J, Cell Signaling Technology) followed by polyclonal goat anti-rabbit antibody (1.50; BioLegend).

Sample acquisition was performed on a BD Biosciences LSRII Fortessa or Symphony A5 instrument with FACSDiva (v9.1; BD Biosciences), and data were analysed using FlowJo software (v10.8.1; BD Biosciences).

### Mice, virus and infection model

Six- to 8-week-old female C57BL/6J and CD45.1 congenic mice were purchased from the Jackson Laboratory. The following housing conditions for the mice were used: 12-h-light cycle (7:00 am on, 7:00 pm off), temperature between 68–74 °F, humidity between 30–70 g m^–^^3^. Chronically LCMV-infected mice were generated as follows; mice were transiently depleted of CD4^+^ T cells by injecting them with 300 μg of GK1.5 antibody intraperitoneally 2 d before infection and again on the day of infection, followed by infection of mice with 2 × 10^6^ pfu of LCMV clone 13 intravenously in the tail vein. Titres of virus were determined by plaque assay on Vero E6 cells. All animal experiments were performed in accordance with National Institutes of Health and Emory University Institutional Animal Care and Use Committee guidelines.

### Mouse tumour models

#### Panc02-H7-Fluc

Orthotopic and subcutaneous syngeneic models were used to assess the in vivo efficacy of muPD1-IL2v compared with the single agents muPD1 and muFAP-IL2v or their combination in C57BL/6J mice. A subcutaneous syngeneic model was used to assess the in vivo efficacy of PD1-IL2v compared with pembrolizumab and aldesleukin or their combination in human PD-1-transgenic C57BL/6J mice (University of Oxford). Survival and tumour growth inhibition were the readouts for the orthotopic and subcutaneous models, respectively. In brief, 6- to 8-week-old female C57BL/6J mice (Charles River) were inoculated with 1 × 10^5^ Panc02-H7-Fluc cells injected into the pancreas (pancreatic orthotopic model) or 5 × 10^5^ Panc02-H7-Fluc cells injected subcutaneously (subcutaneous model). Mice were maintained under specific-pathogen-free conditions with daily cycles of 12 h light/12 h darkness according to guidelines (temperature of 22 °C, dark/light cycle of 12 h and humidity of 50%; GV-SOLAS, FELASA), and food and water were provided ad libitum. Continuous health monitoring was carried out, and the experimental study protocol was reviewed and approved by the Veterinary Department of Canton Zurich.

Mice were randomized into different treatment groups, and therapy started when evidence of tumour growth was visible in the target organ by bioluminescence (7 d after tumour cell inoculation) in the orthotopic model or when tumours reached an average volume of 200 mm^3^ as measured by calliper in the subcutaneous model. All treatments were administered intravenously, and the following doses were investigated: muPD1-IL2v at 0.5 and 1 mg kg^–1^, muFAP-IL2v at 2.5 mg kg^–1^, muPD1 at 10 mg kg^–1^, PD1-IL2v at 0.5 mg kg^–1^, pembrolizumab at 10 mg kg^–1^, aldesleukin at 900,000 IU kg^–1^ twice daily. The termination criterion for the orthotopic model to kill animals was sickness with locomotion impairment, and median overall survival was defined as the experimental day by which 50% of animals had been killed. Kaplan–Meier survival curves and the pairwise log-rank test were used to compare survival between animal treatment groups. In the subcutaneous model, tumour growth inhibition was used as the readout; to test for significant differences in group means for multiple comparisons, standard ANOVA (one-way ANOVA) was used with Dunnett’s method. The JMP statistical software program was used for analyses.

In the orthotopic model, pancreatic carcinoma cell line Panc02-H7-Fluc was used in an orthotopic pancreatic model. In brief, a median laparotomy was performed under deep general anaesthesia and the pancreas was exposed. Aliquots of 1 × 10^5^ Panc02-H7-Fluc cells were injected into the pancreas. The pancreas was replaced and the abdominal wall was closed. Therapy was started 7 d after tumour cell inoculation when solid tumours in the pancreas were observed by means of bioluminescence imaging.

In the subcutaneous model, pancreatic carcinoma cell line Panc02-H7-Fluc (5 × 10^5^ cells) was injected into the subcutis of the left flank. Tumour volume was measured using a calliper. Tumour volume was calculated with the formula:

$${\rm{Tumour\; volume}}={\rm{length}}\times {\rm{width}}\times {\rm{depth}}\times 4/3\pi $$

Therapy was started when tumour volume reached 150–200 mm^3^.

For the CD8^+^ depletion study, all mice bearing Panc02-H7-Fluc subcutaneous tumours were administered intravenously with anti-mouse CD8 antibody (InVivoPlus Anti-Mouse CD8α, clone 2.43, BioXCell), 5 mg kg^–1^ three times a week, 1 week before the first administration of therapy. Depletion of mouse CD8^+^ cells was evaluated in blood by flow cytometry before the start of therapy.

#### B16F10-OVA

A subcutaneous melanoma syngeneic model was used to assess the in vivo efficacy of muPD1-IL2v compared with the single agents muPD1 and muFAP-IL2v or their combination in C57BL/6J mice. Tumour growth inhibition and survival rate were the readouts for this subcutaneous model. In brief, 6- to 8-week-old female C57BL/6J mice (Charles River) were inoculated subcutaneously with 2 × 10^5^ B16F10-OVA cells from a B16 cell line overexpressing the OVA protein. As described previously for the Panc02-H7-Fluc model, mice were maintained under specific-pathogen-free conditions with daily cycles of 12 h light/12 h darkness according to guidelines (GV-SOLAS, FELASA) and food and water were provided ad libitum. Continuous health monitoring was carried out, and the experimental study protocol was reviewed and approved by the Veterinary Department of Canton Zurich. Tumour volume was measured using a calliper. Tumour volume was calculated with the formula:

$${\rm{Tumour\; volume}}={\rm{length}}\times {\rm{width}}\times {\rm{depth}}\times 4/3\pi $$

Mice were randomized, 10 d after tumour inoculation, into different treatment groups on the basis of tumour size. Therapy started 11 d after tumour inoculation. All treatments were administered subcutaneously once a week, with the following doses investigated: muPD1-IL2v at 0.5 mg kg^–1^, muFAP-IL2v at 1.5 and 3 mg kg^–1^, combination of muPD1 at 10 mg kg^–1^ and FAP-IL2v at 1.5 mg kg^–1^. For survival rate curves, the termination criterion to kill animals was either tumour size or tumour ulceration. Tumour growth inhibition was an additional readout; to test for significant differences in group means for multiple comparisons, standard ANOVA (one-way ANOVA) was used with Dunnett’s method. GraphPad Prism software (v8) was used for graphical representation and analyses.

#### MCA-205 sarcoma

A subcutaneous fibrosarcoma syngeneic model was also used to assess the in vivo efficacy of muPD1-IL2v compared with the single agents muPD1 and muFAP-IL2v or their combination in C57BL/6J mice at the CRO Antineo (Lyon, France). Tumour growth inhibition was the readout for this subcutaneous model. In brief, 6- to 8-week-old female C57BL/6J mice (Charles River) were inoculated with 5 × 10^5^ MCA-205 cells injected subcutaneously. Mice were maintained under specific-pathogen-free conditions with continuous health monitoring according to guidelines (Animalerie Commune Scar Rockefeller, Lyon, France).

Mice were randomized into different treatment groups, and therapy started when tumours reached an average volume of 100 mm^3^ as measured by calliper in the subcutaneous model. All treatments were administered intravenously, with the following doses investigated: muPD1-IL2v at 1 and 2 mg kg^–1^, muFAP-IL2v at 2 mg kg^–1^ and muPD1 at 3 mg kg^–1^. Tumour volume was measured using a calliper and calculated with the formula:

$${\rm{Tumour\; volume}}={\rm{length}}\times {\rm{width}}\times {\rm{depth}}\times 4/3\pi $$

Tumour growth inhibition was used as the readout; to test for significant differences in group means for multiple comparisons, standard ANOVA (one-way ANOVA) was used with Dunnett’s method. The JMP statistical software program was used for analyses.

#### RIP-Tag5 transgenic mouse model of PanNET

The generation of RIP-Tag5 mice has previously been described^52^. The RIP-Tag5 mice in this study were on a C57BL6/N background (Charles River) and were males aged 21 to 31 weeks. Animal experiments were conducted according to protocols approved by the Veterinary Authorities of the Canton of Vaud and Swiss law.

To enrol RIP-Tag5 mice into the trial, mice from 22 weeks of age displaying blood glucose levels below 7 mM were screened for the presence of PanNET islet tumours by ultrasound imaging using a Vevo2100 system with an MS550D 40-MHz transducer (Visual Sonic). RIP-Tag5 mice were randomly assigned to different treatment groups on the basis of cumulative tumour burden. The average starting tumour burden was 27 mm^2^, the average starting age was 26 weeks and the average starting glucose level was 5.5 mM. Tumours were monitored by ultrasound imaging every 2 weeks, or every 4 weeks for complete responders, for a maximum of 16 weeks following the start of treatment. Blood glucose levels were monitored weekly using an Accu-Chek glucometer (Roche). The criteria for the endpoint were defined by tumour burden (>50 mm^2^ or 2- to 4-fold increase on progression for relapsing tumours), hypoglycaemia (blood glucose levels at or below 3 mM) and health status.

Therapies were administered by intraperitoneal injection with the following amounts per mouse: muPD1, 250 μg once a week; DP47-muIL2v (untargeted muIL-2v), 25 μg once a week; PD1-IL2v, 25 μg once a week for a duration of 8 weeks.

### Histology

For histological analysis, tissue samples were collected, fixed in 10% formalin (Sigma) and later processed for FFPET (Leica, 1020). Four-micrometre paraffin sections were subsequently cut in a microtome (Leica, RM2235). Haematoxylin and eosin staining was performed in an automated Leica system following the manufacturer’s instructions. Mouse PD-1 immunohistochemistry was performed with anti-mouse PD-1 (1:250; clone AF1021, R&D Systems) while mouse granzyme B staining was performed with anti-mouse GZMB (1:250; clone ab4059, Abcam) on CD3^+^ (1:100; clone SP7, Diagnostic Biosystems) and CD8^+^ (1:300; clone 4SM15, eBiosciences) T cells. Staining was performed in the Leica autostainer (Leica, ST5010) following the manufacture’s protocols. Sections were counterstained with haematoxylin (Sigma-Aldrich), and slides were scanned using the Olympus VS120-L100 Virtual Slide Microscope scanner. Quantification of positive cells from scan images was performed with Definiens software. For this, whole scans were uploaded in the tissue developer module and necrotic areas were excluded with segmentation analysis. Second, a threshold was set to recognize the brown staining of targeted cells, and the algorithm for cell quantification or percentage positive area was subsequently automatically run. The output data were then transferred to GraphPad Prism (v8) for analysis of significance by standard ANOVA (one-way ANOVA) with Dunnett’s correction.

### Cell lines

Vero E6 cells were obtained from the American Type Culture Collection, mouse pancreatic cancer cell line Panc02-H7-Fluc was generated at Roche Glycart and the B16-OVA cell line was purchased from ProQinase. The MCA-205 mouse fibrosarcoma cell line was purchased from Sigma-Aldrich and was derived from 3-methylcholanthrene-induced fibrosarcoma in C57BL/6 mice. Tumours were maintained in vivo by serial subcutaneous transplantation in syngeneic mice, and single-cell suspensions were prepared from solid tumours by enzymatic digestion.From these cells, the MCA-205 cell line was established and maintained in vitro. Vero E6 cells were not authenticated, while MCA-205, B16-OVA and Panc02-H7-Fluc cells were authenticated through morphology and PCR assays with species-specific primers. MCA-205 cells tested negative for infectious diseases using a Mouse Essential CLEAR panel by Charles River Animal Diagnostic Services and were negative for mycoplasma contamination. Batches of the Panc02-H7-Fluc and B16-OVA cell lines are routinely tested in house for mycoplasma and are negative.

### Lymphocyte isolation

#### Chronic infection experiments

Lymphocytes were isolated from the blood, spleen and lung as described previously^53^. In brief, spleens were dissociated by passing them through a 70-μm cell strainer (Corning). Lungs were treated with 1.3 mM EDTA in HBSS for 30  min at 37 °C, with shaking at 200 r.p.m., followed by treatment with 150 U ml^–1^ collagenase (Thermo Fisher Scientific) in RPMI-1640 containing 5% FBS, 1 mM MgCl_2_ and 1 mM CaCl_2_ for 60 min at 37 °C with shaking at 200 r.p.m. Collagenase-treated lung tissues were homogenized and filtered through a 70-μm cell strainer. Lymphocytes from lungs were purified using a 44–67% Percoll gradient (800 g at 20 °C for 20 min).

#### Cancer model experiments

Mice were killed according to animal welfare guidelines; tumour tissue and blood were isolated in the animal facility. Tumour tissue was transferred to PBS and was disrupted using manual scissors and the Miltenyi Gentle MACS machine. Subsequently, it was digested in an enzyme mix consisting of RPMI with 10 mg ml^–1^ DNase (Sigma-Aldrich) and 0.25 mg ml^–1^ Liberase (Sigma-Aldrich).After 30 min of digestion at 37 °C, the tissue mix was filtered through a 70-µm filter and resuspended as a single-cell suspension with an appropriate volume for subsequent staining with fluorescently labelled antibodies. Blood was transferred to heparin tubes, and red blood cells were lysed with erythrocyte lysis buffer. After red blood cell lysis, cells were resuspended as a single-cell suspension with an appropriate volume for subsequent staining with fluorescently labelled antibodies. Lymphocytes were mechanically isolated from draining lymph nodes with a pestle, filtered through a 70-µm filter and resuspended as a single-cell suspension with an appropriate volume for subsequent staining with fluorescently labelled antibodies.

### Reagents, flow cytometry and in vitro stimulation

#### Chronic infection experiments

All antibodies for flow cytometry were purchased from BD Biosciences, BioLegend, Thermo Fisher Scientific, Cell Signaling Technology and R&D Systems. D^b^GP33–41 and D^b^GP276–286 tetramers were prepared in house and were used to detect LCMV-specific CD8^+^ T cells. Streptavidin-PE or streptavidin-APC was purchased from Thermo Fisher Scientific. Dead cells were excluded by using the Live/Dead Fixable Near-IR or Yellow Dead Cell Stain kit (Thermo Fisher Scientific). For cell-surface staining, antibodies were added to cells at dilutions of 1:20 to 1:500 in PBS supplemented with 2% FBS and 0.1% sodium azide for 30 min on ice. Cells were washed three times and fixed with 2% paraformaldehyde. To detect cytokine production, 1 × 10^6^ splenocytes were stimulated with a pool of nine LCMV-specific peptides (200 ng ml^–1^ each of GP33–41, GP70–77, GP92–101, GP118–125, GP276–286, NP166–175, NP205–212, NP235–249 and NP396–404) in a 96-well round-bottom plate for 5 h at 37 °C in a CO_2_ incubator in the presence of GolgiPlug (BD Biosciences). Samples were acquired on a Canto II, LSR II or FACSymphony A3 instrument (BD Biosciences) with FACSDiva (v9.1; BD Biosciences), and data were analysed by using FlowJo (v9.9.6 or v10.8.1; BD Biosciences).

#### Cancer model experiments

Single-cell suspensions from tumours and blood were stained with the following antibodies: Fixable Viability Dye eFluor 455UV (1:500), AF700 anti-CD45 (1:300; clone 30-F11, BioLegend), PercP-Cy5.5 anti-TCRβ (1:200; clone H57-597, BioLegend), APC-Cy7 anti-CD8 (1:200; clone 53-6.7, BioLegend), PE-Cy7 anti-CD4 (1:200; clone GK1.5, BioLegend), FITC anti-CD62L (1:200; clone MEL-14, BioLegend), PE anti-CD127 (1:100; clone A7R34, BioLegend), BV421 anti-CD4 (1:200; clone GK1.5, BioLegend), AF647 anti-granzyme B (1:100; clone GB11, BioLegend), BV786 anti-IFNγ (1:100; clone XMG1.2, BioLegend), PE-Cy7 anti-TNFα (1:100; clone MP6-XT22, BioLegend), BV421 anti-FoxP3 (1:100; clone MF-14, BioLegend), AF647 anti-CD39 (1:200; clone Duha59, BioLegend), AF700 anti-granzyme B (1:100; clone QA16A02, BioLegend), PE-Cy7 anti-Ki67 (1:300; clone 16A8, BioLegend), PE-Cy7 anti-PD-1 (1:200; clone RMP1-30, BioLegend), BV711 anti-CD25 (1:200; clone RMT3-23, BioLegend), PE-Dazzle594 anti-TIGIT (1:100; clone 1G9, BioLegend), BV605 anti-IFNγ (1:100; clone XMG1.2, BioLegend), BV421 anti-TNFα (1:100; clone MP6-XT22, BioLegend), AF488 anti-CD107a (1:100; clone 1D4B, BioLegend), BV510 anti-CD44 (1:200; clone IM7, BD Biosciences), BUV805 anti-CD45 (1:100; clone 30-F11, BD Biosciences), BV786 anti-TCRβ (1:100; clone H57-597, BD Biosciences), BUV496 anti-CD4 (1:100; clone RM4-5, BD Biosciences), BUV395 anti-CD8 (1:100; clone 53-6.7, BD Biosciences), BUV737 anti-PD-1 (1:100; clone RMP1-30, BD Biosciences), PE-CF594 anti-CD25 (1:100; clone PC61, BD Biosciences), BV650 anti-TIM-3 (1:100; clone 5D12, BD Biosciences), PE anti-TCF-1 (1:100; clone S33-966, BD Biosciences), BV650 anti-LAG3 (1:100; clone C9B7W, BD Biosciences), BV510 anti-SLAMF6 (1:50; clone 13G3, BD Biosciences) and FITC anti-CD218a (1:50; clone REA947, Miltenyi).

Detection of OVA-specific CD8^+^ T cells was performed by using APC-labelled Dextramer H-2Kb (SIINFEKL) from Immudex (1:100). Staining with Dextramer was performed by using 0.1% BSA in PBS. For intracellular staining, cells were fixed and permeabilized using the FOXP3 Transcription Factor Staining Buffer Set from eBioscience or the Transcription Buffer Set from BD.

For detection of cytokines, tumour cell suspensions were re-stimulated with 6.25 ng ml^–1^ PMA (Sigma-Aldrich) and 1.87 μg ml^–1^ ionomycin (Sigma-Aldrich) for 5 h at 37 °C. After 1 h of re-stimulation, GolgiPlug (BD) and GolgiStop (BD) were added to the cell suspension. For antigen re-stimulation, tumour cell suspensions were re-stimulated with 0.1 µg ml^–1^ gp100 or SIINFEKL peptide, for 5 h at 37 °C. Anti-CD107a antibody was added together with the peptides for 5 h at 37 °C. As before, after 1 h of re-stimulation, GolgiPlug (BD) and GolgiStop (BD) were added to the cell suspension.

Discrimination of living cells from dead cells was performed using DAPI (Sigma-Aldrich), Fixable Viability Dye eFluor 780 (eBioscience) or Live/Dead APC-Cy7 (eBioscience). Samples were acquired with a BD LSRII Fortessa and a BD FACSymphony A5 instrument by using FACSDiva (v9.1; BD Biosciences). Data obtained were analysed by using FlowJo (v10.8.1; BD Biosciences).

### Cell sorting

#### Chronic infection experiments

Cell sorting was performed on a FACSAria II (BD Biosciences). For adoptive transfer experiments, two PD-1-expressing CD8^+^ T cell subsets (PD-1^+^CXCR5^+^TIM-3^−^ and PD-1^+^CXCR5^−^TIM-3^+^) were sorted from the pooled spleens (*n* = 40–60) of chronically LCMV-infected mice. For RNA-seq analysis of LCMV-specific CD8^+^ T cells after muPD-L1, muPD1-IL2v and muPD-L1 + muPD1-IL2v therapy, chronically LCMV-infected mice (more than 40 d after infection; *n* = 1–18) were left untreated or treated for 2 weeks, and D^b^GP33^+^CD8^+^ T cells were sorted from pooled spleens to obtain at least 2 × 10^4^ cells. Naive (CD44^low^) CD8^+^ T cells were sorted from the pooled spleens of uninfected mice (*n* = 2–3). All samples had purities of greater than 95%.

#### Cancer model experiments

Single-cell tumour suspensions were kept on ice during the staining and sorting procedure. Cell suspensions from 3–5 tumours of the same treatment group were stained with the following antibodies: AF700 anti-CD45 (1:100; clone 30-F11, BioLegend), BV711 anti-CD8 (1:100; clone 53-6.7, BioLegend) and bin channel BV605 anti-CD4 (1:100; clone GK1.5, BioLegend), and BV605 anti-CD11c (1:100; clone N418, BioLegend). Discrimination of living cells from dead cells was performed using Live/Dead APC-Cy7 (eBioscience, 65-0865-14; 1:500 for non-fixed samples and cells with incubation for 20 min). Cells were washed twice, filtered through a 40-µm cell strainer, sorted on a FACSAria III instrument and acquired with FACSDiva (to enrich viable single CD45^+^CD8^+^CD11c^−^CD4^−^ cells.

### Mouse PD-L1 blockade and treatment with muFAP-IL2wt, muFAP-IL2v and muPD1-IL2v in vivo

For PD-L1 blockade, 200 μg of mouse muPD-L1 antibody with the DAPG mutation (Roche) was administered intraperitoneally into chronically LCMV-infected mice every 3 d for 2 weeks. Appropriate isotype control (mouse IgG1 isotype control (MOPC-21, BioXCell)) was administered in untreated mice. For muFAP-IL2 therapy, 1 mg kg^–1^ muFAP-IL2wt or muFAP-IL2v was administered intraperitoneally into chronically LCMV-infected mice twice weekly for 2 weeks. For muPD1-IL2v therapy, 1 mg kg^–1^ muPD1-IL2v was administered intraperiotenally into chronically LCMV-infected mice twice weekly for 2 weeks.

### Adoptive transfer of two CD8^+^ T cell subsets

Cells from two CD8^+^ T cell subsets (4–8 × 10^4^ cells; PD-1^+^CXCR5^+^TIM-3^−^ and PD-1^+^CXCR5^−^TIM-3^+^) isolated from chronically LCMV-infected mice (CD45.2^+^) were transferred into infection-matched recipient mice (CD45.1^+^), followed by muPD1-IL2v treatment for 2 weeks.

### RNA isolation and RNA-seq

#### Chronic infection experiments

Total RNA was extracted by using the RNeasy Micro kit (Qiagen) or Direct-zol RNA Microprep kit (Zymo Research) according to the manufacture’s protocols at the Emory Integrated Genomics Core or in house. Preparation of a standard RNA-seq library was performed at Hudson Alpha or the Emory Yerkes Nonhuman Primate Research Center (NPRC) Genomics Core. In brief, RNA amplification was performed using the Nugen Ovation RNAseq v2 kit or Clontech SMART-Seq v4 Ultra Low Input RNA kit (Takara Bio). Amplified cDNA was fragmented, and samples were prepared using the KAPAHyper Prep kit or Nextera XT DNA Library Preparation kit (Illumina). Pooled libraries were sequenced on an Illumina NovaSeq 6000 with 100-bp paired-end reads.

#### Cancer model experiments

Tumours and lymph nodes were digested as previously described, and 1–10 Mio cells were stored in liquid nitrogen in Ibidi freezing medium. The samples were randomized and processed in four different batches with ten samples each (tumours and lymph nodes were processed separately). After thawing a batch of samples, the cells were stained with a mix of flow cytometry and oligonucleotide-labelled antibodies (Table 1) and sorted for CD8^+^ T cells before performing scRNA-seq. In brief, cells were washed once with PBS before evaluation of both cell number and viability using a Nexcelom Cellometer Auto 2000. Approximately one Mio cell per sample was resuspended in 50 µl PBS and incubated with 5 µl of Mouse TruStain FcX Fc Blocking reagent (BioLegend). The mix of flow cytometry and oligonucleotide-labelled antibodies was added to the cells in a volume of 50 µl (final volume, 100 µl). After incubation for 30 min at 4 °C, the cells were washed three times with PBS and resuspended in 500 µl PBS to obtain a concentration of approximately 1 × 10^6^ cells per ml. Cells were filtered through a 40-µm cell strainer and sorted on the BD FACSAria III system. The cell number and viability of the sorted cells were determined using a Nexcelom Cellometer Auto 2000, and a total of 10,000 viable cells per sample were loaded into the 10x Genomics Chromium controller. cDNA and library preparation were performed according to the manufacturer’s indications (scRNA-seq 5′ v2 kit with TCR and feature barcoding), and the resulting libraries were sequenced on an Illumina NovaSeq 6000 sequencer according to 10x Genomics recommendations (R1 = 26, i7 = 10, i5 = 10, R2 = 90) to a depth of approximately 20,000 reads per cell for the GEX library and 5,000 reads per cell for both the TCR and feature barcoding libraries.

**Table 1:** Antibodies labelled with a unique oligonucleotide tag identifier for CITE-seq.

Application	Marker	Clone	Oligonucleotide tag	Source	Concentration
Feature barcoding	CD28	37.51	ATTAAGAGCGTGTTG	TotalSeq-C, BioLegend	1 µg ml^–1^
Feature barcoding	CD44	IM7	TGGCTTCAGGTCCTA	TotalSeq-C, BioLegend	1 µg ml^–1^
Feature barcoding	CD62L (L-selectin)	MEL-14	TGGGCCTAAGTCATC	TotalSeq-C, BioLegend	1 mg ml^–1^
Feature barcoding	CD39	Duha59	GCGTATTTAACCCGT	TotalSeq-C, BioLegend	1 µg ml^–1^
Feature barcoding	CD279 (PD-1)	RMP1-30	GAAAGTCAAAGCACT	TotalSeq-C, BioLegend	1 µg ml^–1^
Feature barcoding	CD366 (TIM-3)	RMT3-23	ATTGGCACTCAGATG	TotalSeq-C, BioLegend	1 µg ml^–1^
Feature barcoding	CD223 (LAG-3)	C9B7W	ATTCCGTCCCTAAGG	TotalSeq-C, BioLegend	1 µg ml^–1^
Feature barcoding	CD183 (CXCR3)	CXCR3-173	GTTCACGCCGTGTTA	TotalSeq-C, BioLegend	1 µg ml^–1^
Feature barcoding	CD185 (CXCR5)	L138D7	ACGTAGTCACCTAGT	TotalSeq-C, BioLegend	1 µg ml^–1^
Feature barcoding	CD127 (IL-7Rα)	A7R34	GTGTGAGGCACTCTT	TotalSeq-C, BioLegend	1 µg ml^–1^
Feature barcoding	TIGIT (Vstm3)	1G9	GAAAGTCGCCAACAG	TotalSeq-C, BioLegend	1 µg ml^–1^
Feature barcoding	CD25	PC61	ACCATGAGACACAGT	TotalSeq-C, BioLegend	1 µg ml^–1^
Feature barcoding	Ly108 (SLAM-F6)	330-AJ	CGATTCTTTGCGAGT	TotalSeq-C, BioLegend	1 µg ml^–1^
Feature barcoding	CD137 (4-1BB)	17B5	TCCCTGTATAGATGA	TotalSeq-C, BioLegend	1 µg ml^–1^
Feature barcoding	IL-21R	4A9	GATTCCGACAGTAGA	TotalSeq-C, BioLegend	1 µg ml^–1^

### Analysis of RNA-seq data for virus-specific CD8^+^ T cells during chronic infection

Reads were mapped to the GRCm38/mm10 genome^54^ with HISAT2 (v2.1.0)^55^. Gene expression was quantified with featureCounts^56^ (v1.5.2). DESeq2 (ref.^57^; v1.24.0) was used to normalize for library size and calculate differential expression across groups. A gene was considered differentially expressed between two groups with an adjusted *P* value of <0.05 and an average expression of >20 normalized counts across all samples. PCA was performed on all detected genes using the regularized log transformation from DESeq2. RNA-seq data were visualized by using Prism software (v9.3.1; GraphPad) and the ComplexHeatmap R package (v2.2.0)^58^.

### Single-cell RNA, protein and TCR sequencing analyses of TILs

Fastq files were aligned to the mouse transcriptome (mm10-2020-A) using CellRanger (count and vdj) v6.0.0 with the parameter ‘--expect-cells = 6000’. All cells showing >200 counts were further merged across all samples and processed with scanpy^59^ and the besca^60^ standard workflow. Filtering was performed with the parameters min_genes = 500, min_cells = 20, min_counts = 1000, n_genes = 6000, percent_mito = 0.08, max_counts = 40000. In addition, cells with no antibody counts were removed. Two samples were excluded because of low overall quality and very low cell number; all other samples were included in the analysis. In brief, RNA counts were normalized per 10,000, the top most highly variable genes were selected, total gene and mitochondrial reads were regressed out, PCA was performed and the first 50 principal components were used for nearest-neighbour calculations and Leiden clustering, as well as for UMAP-based visualization. Protein counts were central log ratio (CLR) transformed. Annotation was performed using besca’s sig-annot module, and more detailed CD8^+^ subpopulations were attributed on the basis of RNA and protein marker expression and signature enrichment (scanpy.tl.score_genes). Only clusters containing CD8^+^ T cell populations were retained in the analysis; clusters 21 (non-immune), 18 (non-T cell), 7 and 19 (myeloid T cell doublet), 22 (T helper 17 cell) and 20 (T_reg_ cell) were excluded as likely contaminants. Gene signatures used in Fig. 4 are provided in Table 2. TCR analysis was performed in Python with the toolkit scirpy^61^, and clonotypes were determined on the basis of CDR3 sequence identity, with the parameters receptor_arms = "all", dual_ir = "primary_only". Jupyter notebooks are available for data preprocessing, clustering and visualization, and cell annotation, as well as for TCR analysis, at https://github.com/bedapub/PD1-IL2v_in-vivo_TILs_Panc02_publication.

**Table 2:** Gene signatures used for Fig. 4 and Extended Data Fig. 8.

Migration	Immune checkpoint	Stem-like CD8^+^ T cells (cluster 6, vehicle)	Exhausted CD8^+^ T cells	Cytotoxicity
*Ccr2*	*Cd160*	*Pag1*	*Pdcd1*	*Gzma*
*Cxcr3*	*Lag3*	*Slco3a1*	*Havcr2*	*Gzmb*
*Cxcr4*	*Cd244a*	*Ifit1bl1*	*Lag3*	*Gzmc*
*Cx3cr1*	*Btla*	*Itgae*	*Entpd1*	*Gzmf*
*S1pr1*	*Pdcd1*	*Baiap3*	*Cd38*	
*Itga1*	*Havcr2*	*Ripor2*	*Tox*	
*Itga4*	*Tigit*	*Rasa3*		
*Itgae*	*Cd101*	*Ly6a*		
*Itgb1*		*Oas3*		
*Itgb7*		*Samhd1*		
*Cd44*		*Gm45552*		
*Ly6c2*		*Cxcr3*		
*Cxcr5*		*Acss2*		
		*Gpr55*		
		*Ifi208*		
		*Arl4c*		
		*Ifi213*		
		*Ccr2*		
		*5830432E09Rik*		
		*Ly9*		
		*Rtp4*		
		*Nod1*		
		*Dtx1*		
		*Slfn1*		

### Internalization assays

#### Imaging

Human CD4^+^ T cells freshly purified from PBMCs were activated on anti-CD3/CD28-coated plates. Three days after activation, CD4^+^ T cells were collected and stained with 10 μM CellTracker Blue CMAC Dye (Invitrogen, C2110) for 15 min at 37 °C; 150,000 cells were seeded on RetroNectin-coated imaging slides and allowed to adhere for 30 min at 37 °C. For RetroNectin coating, imaging slides were treated with 1 μg μl^–1^ RetroNectin (Takara, T100B) for 40 min at room temperature. Subsequently, T cells were treated for 1 h or 3 h at 4 °C or 37 °C with 630 pM (0.1 μg ml^–1^) of in-house-produced FAP-IL2v AF647 or 630 pM (0.1 μg ml^–1^) of in-house-produced PD1-IL2v AF647. Where indicated, cells were pretreated with 10 μg ml^–1^ anti-PD-1 to saturate all PD-1 binding sites. Afterwards, samples were fixed and permeabilized (BD Cytofix/Cytoperm, 554714) for 20 min at 4 °C and then stained with a non-competing unconjugated anti-PD-1 antibody (1:100; D4W2J, Cell Signaling Technology) for 45 min at 4 °C, followed by staining with goat anti-rabbit IgG (H+L), F(ab′)_2_ Fragment AF488 (Cell Signaling Technology, 4412, lot 18; 2 mg ml^–1^; 1:1,000). All samples were then imaged with an inverted confocal microscope (Leica Sp8), adopting a ×40 objective. For each image, one optical section was acquired with a resolution of 1,024 × 1,024 at a pixel size of 0.379 μm.

#### Data analysis

Images were analysed using Imaris 9.5.1 (Bitplane), MATLAB 2020a (Mathworks) and GraphPad Prism (v8; GraphPad Software). In Imaris, images were opened and the cytoplasm was segmented on the basis of the CellTracker Blue CMAC Dye channel (surface grain size, 0.758). Subsequently, the images were transformed to 32 bits; then, using the Imaris Xtension ‘Distance Transformation’, the distance to the ‘cytoplasm isosurface’ was calculated and saved as a separate channel. The next segmentation, to approximate the membrane position, was based on this ‘distance to cytoplasm’ channel (surface grain size, 0.758; thresholds, 0–0.759 µm for distance from cytoplasm). Any touching segmentation objects were split, such that for every segmented cytoplasm a segmented membrane was present. Membrane and cytoplasm statistics were exported for further analysis.

A custom MATLAB script was used to match the values for ‘drug average intensity’ and ‘PD-1 average intensity’ of membrane and cytoplasm objects, on the basis of the closest distance for segmented object centroids. Subsequently, the ratio was calculated and exported as a .csv file. From the exported .csv files, values were copied into GraphPad Prism for plotting and statistical analysis.

### Statistical analysis

Prism software (v8 and v9.3.1; GraphPad) was used for statistical analysis. Differences among the experimental groups were assessed by using an unpaired test or a Mann–Whitney test for comparing two groups. One-way or two-way ANOVA with Dunnett’s or Tukey’s post hoc test or a Kruskal–Wallis test was used for comparison of more than two groups. To test for significant differences in tumour growth inhibition between group means for multiple comparisons, standard ANOVA (one-way ANOVA) was used with Dunnett’s post hoc test in the Panc02 mouse tumour model. Log-rank Mantel–Cox tests were used to compare muPD1-IL2v versus muPD1 + untargeted muIL-2v survival in the RIP-Tag5 mouse tumour model; Wilcoxon’s test was used for comparison of survival in the orthotopic Panc02 mouse tumour model.

### Reporting summary

Further information on research design is available in the Nature Research Reporting Summary linked to this article.

## Online content

Any methods, additional references, Nature Research reporting summaries, source data, extended data, supplementary information, acknowledgements, peer review information; details of author contributions and competing interests; and statements of data and code availability are available at 10.1038/s41586-022-05192-0.

## Supplementary information

Supplementary Information This file contains Supplementary Figs. 1–25, Supplementary Table 1 and Supplementary Notes.

Reporting Summary

## Data Availability

The scRNA-seq, TCR-seq and CITE-seq data discussed in this publication have been deposited in ArrayExpress with accession number E-MTAB-11773. RNA-seq data from chronic infection experiments are available in the NCBI Gene Expression Omnibus database under accession number GSE208556. Source data are provided with this paper.

## Source data

Source Data Fig. 2
Source Data Fig. 3
Source Data Fig. 4
Source Data Fig. 5
Source Data Extended Data Fig. 1
Source Data Extended Data Fig. 2
Source Data Extended Data Fig. 5
Source Data Extended Data Fig. 6
Source Data Extended Data Fig. 7
Source Data Extended Data Fig. 9

## Code availability

For scRNA-seq, TCR-seq and CITE-seq data analysis, we used CellRanger (v6.0.0), scanpy (v1.6.0), besca (v2.4) and scirpy (v0.7.1); all additional custom workbooks are available at https://github.com/bedapub/PD1-IL2v_in-vivo_TILs_Panc02_publication. Custom code for RNA-seq analysis in the chronic infection model is available from the corresponding authors on reasonable request.
