# Supplementary material for: PD-1-cis IL-2R agonism yields better effectors from stem-like CD8+ T cells
Source: Nature. 2022 Sep 28;610(7930):161–72. doi: 10.1038/s41586-022-05192-0 (PMC9534752; doi:10.1038/s41586-022-05192-0)
Supplement: Supplementary file 1 — This file contains Supplementary Figs. 1–25, Supplementary Table 1 and Supplementary Notes. [file 41586_2022_5192_MOESM1_ESM.pdf]

nature portfolio

https://doi.org/10.1038/s41586-022-05192-0

Supplementary information

PD-1-*cis* IL-2R agonism yields better effectors from stem-like CD8 T cells

In the format provided by the authors and unedited

Cell Sorting

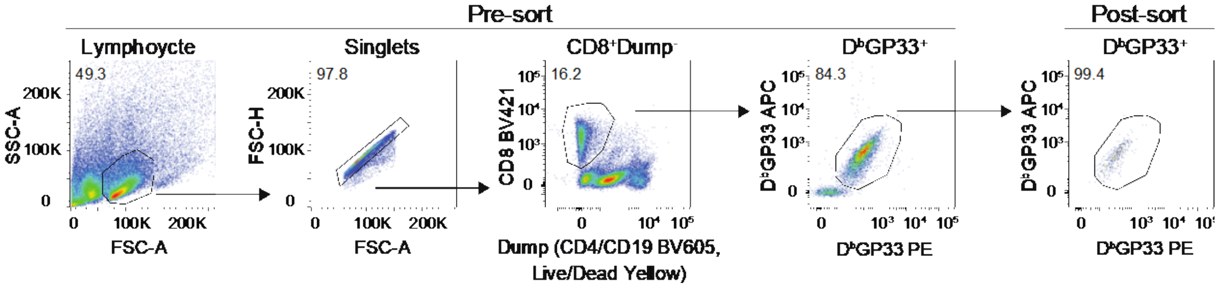

Supplementary Figure 1. Gating strategy for sorting D^{b}GP33^{+} CD8^{+} T cells in Fig. 2c and Extended Data Fig. 5g, h. Splenocytes isolated from chronically LCMV-infected mice were gated on lymphocytes, singlets, live CD8^{+} T cells, and D^{b}GP33^{+} CD8^{+} T cells were sorted.

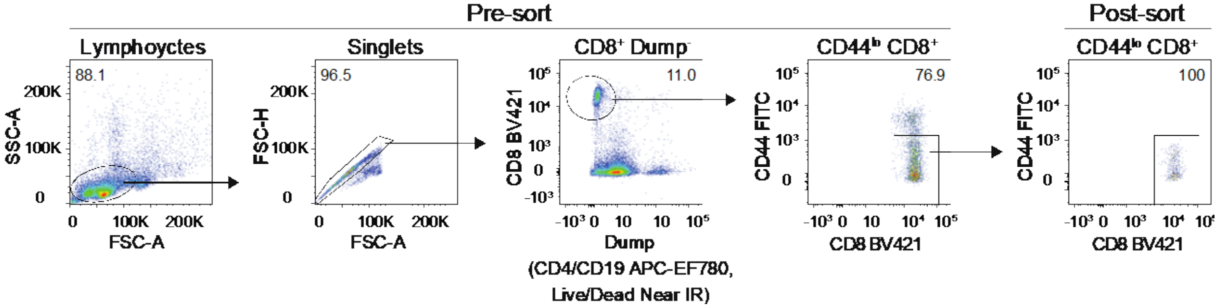

Supplementary Figure 2. Gating strategy for sorting naive CD8^{+} T cells in Fig. 2c. Splenocytes isolated from uninfected C57BL6/J mice were gated on lymphocytes, singlets, live CD8^{+} T cells, and CD44^{lo} CD8^{+} T cells were sorted. EF, eFluor.

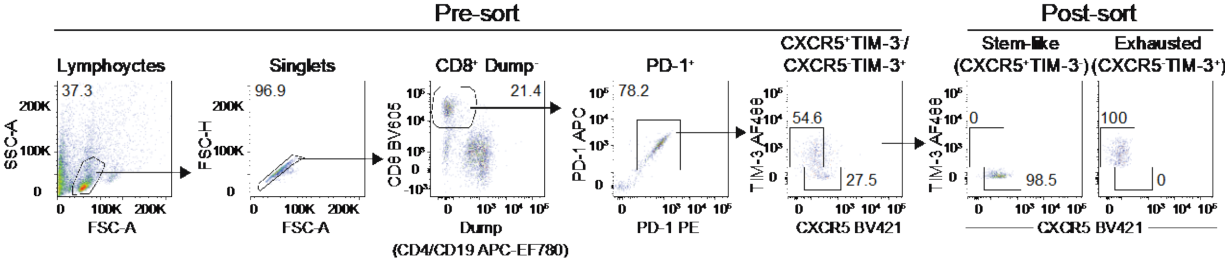

Supplementary Figure 3. Gating strategy for sorting two PD-1^{+} CD8^{+} T-cell subsets in Fig. 2f-h and Extended Data Fig. 6f, g. Splenocytes isolated from chronically LCMV-infected mice were gated on lymphocytes, singlets, CD8^{+} T cells, PD-1^{+} CD8^{+} T cells, and PD-1^{+}CXCR5^{+}TIM-3^{-} and PD-1^{+}CXCR5^{-}TIM-3^{+} CD8^{+} T cells were sorted. EF, eFluor; AF, Alexa Fluor.

T cell analysis on CD8$^+$ T cells

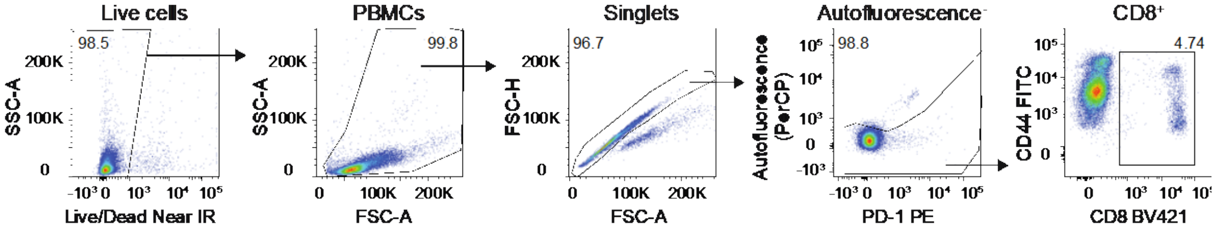

Supplementary Figure 4. Gating strategy for CD8$^+$ T cells in Extended Data Fig. 2b-e. PBMCs isolated from chronically LCMV-infected mice were gated on live PBMCs, singlets, autofluorescence$^-$ cells, and CD8$^+$ T cells**.**

T cell analysis on LCMV-specific CD8$^+$ T cells

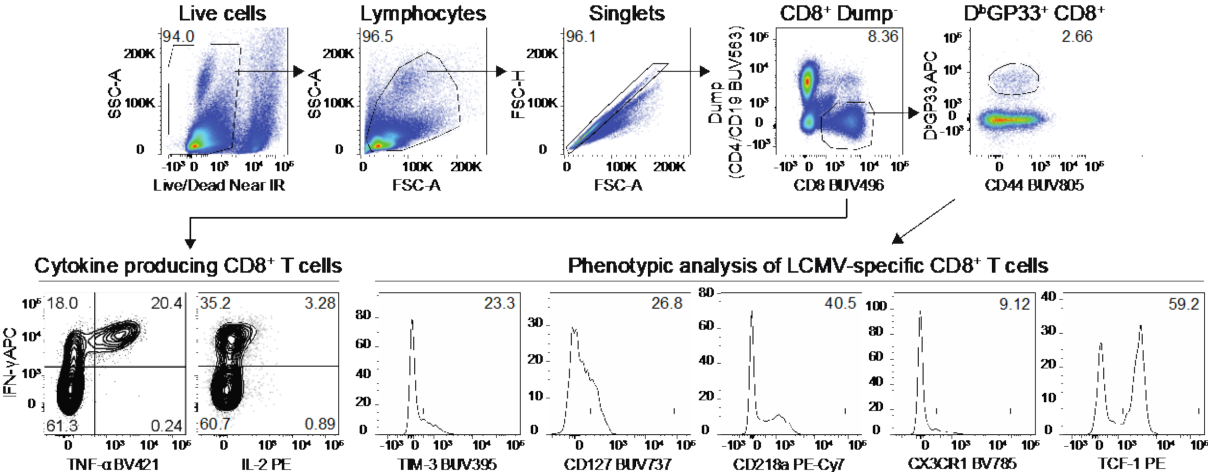

Supplementary Figure 5. Gating strategy for LCMV-specific CD8$^+$ T cells in Fig. 2a, b, d, and Extended Data Fig. 6a, b, d, e. Cells isolated from various tissues in chronically LCMV-infected mice were gated on live cells, lymphocytes, singlets, CD8$^+$ T cells, and LCMV-specific D^{b}GP33$^+$ CD8$^+$ T cells were identified**.** Cytokine producing LCMV-specific CD8$^+$ T cells and LCMV-specific tetramer$^+$ CD8$^+$ T cells were analyzed.

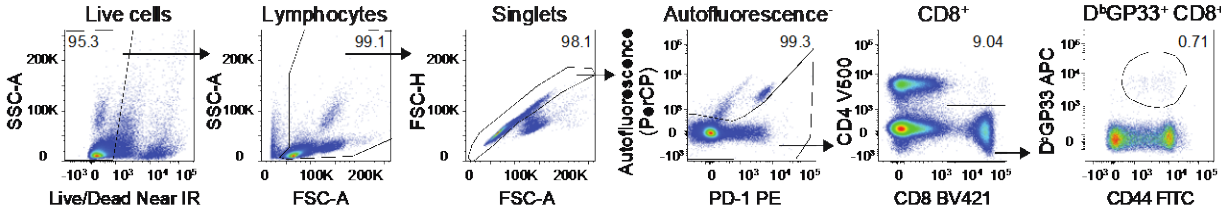

Supplementary Figure 6. Gating strategy for LCMV-specific CD8$^+$ T cells in Extended Data Extended Data Fig. 5c, d. Cells isolated from various tissues in chronically LCMV-infected mice were gated on live cells, lymphocytes, singlets, autofluorescence$^-$ cells, CD8$^+$ T cells, and LCMV-specific D^{b}GP33$^+$ CD8$^+$ T cells were analyzed**.**

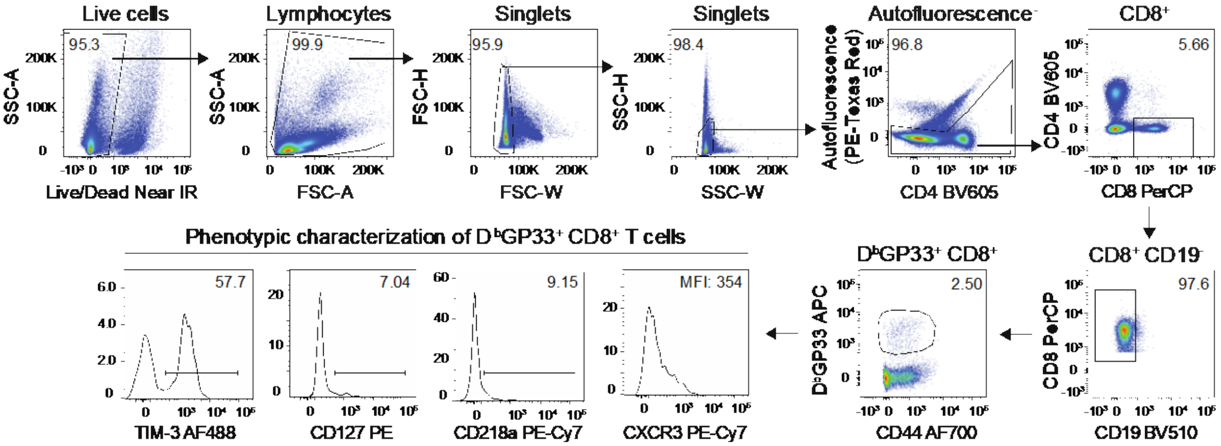

Supplementary Figure 7. Gating strategy for LCMV-specific CD8$^+$ T cells in Extended Data Fig. 1b-d and Extended Data Fig. 5f. Cells isolated from various tissues in chronically LCMV-infected mice were gated on live cells, lymphocytes, singlets, autofluorescence$^-$ cells, CD8$^+$ T cells, CD19$^-$ CD8$^+$ T cells, and LCMV-specific D^{b}GP33$^+$ CD8$^+$ T cells were analyzed**.** AF, Alexa Fluor.

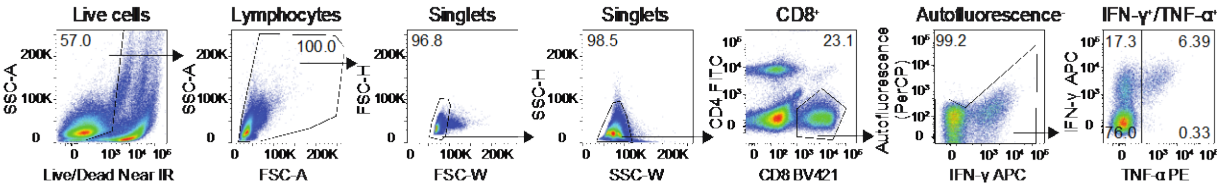

Supplementary Figure 8. Gating strategy for cytokine producing CD8$^+$ T cells in Extended Data Fig. 1e, f and Extended Data Fig. 5e. Splenocytes isolated from chronically LCMV-infected mice were gated on live cells, lymphocytes, singlets, CD8$^+$ T cells, autofluorescence$^-$ cells, and cytokine producing LCMV-specific CD8$^+$ T cells were analyzed**.**

T cell analysis on D^{b}GP33$^+$ CD8$^+$ T cells, Foxp3$^+$ CD4$^+$ T cells, Foxp3$^-$ CD4$^+$ T cells, and naive CD8$^+$ T cells

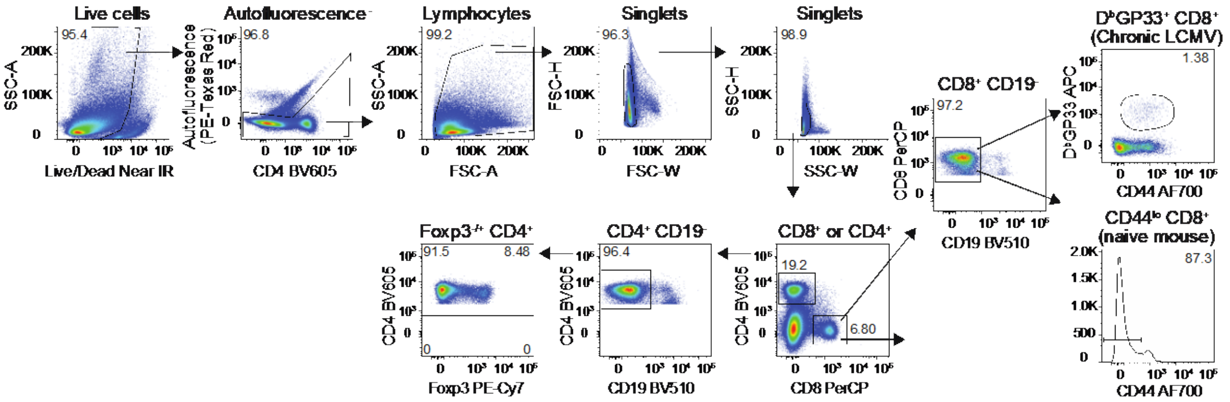

Supplementary Figure 9. Gating strategy for comparing PD-1 expression on D^{b}GP33$^+$ CD8$^+$ T cells, Foxp3$^+$ CD4$^+$ T cells, Foxp3$^-$ CD4$^+$ T cells, and naive CD8$^+$ T cells in Extended Data Fig. 5a. Splenocytes isolated from chronically LCMV-infected mice or uninfected C57BL/6J mice were gated on live cells, autofluorescence$^-$ cells, lymphocytes, and CD8$^+$ and CD4$^+$ T cells were identified. For CD8$^+$ T cells, D^{b}GP33$^+$ CD8$^+$ T cells in chronically LCMV-infected mice and CD44^{lo} CD8$^+$ T cells in uninfected C57BL/6J mice were included in the analysis. For CD4$^+$ T cells, Foxp3$^+$ CD4$^+$ and Foxp3$^-$ CD4$^+$ T cells were analyzed. AF, Alexa Fluor.

T cell analysis on donor CD45.2$^+$ CD8$^+$ T cells in adoptive transfer experiments

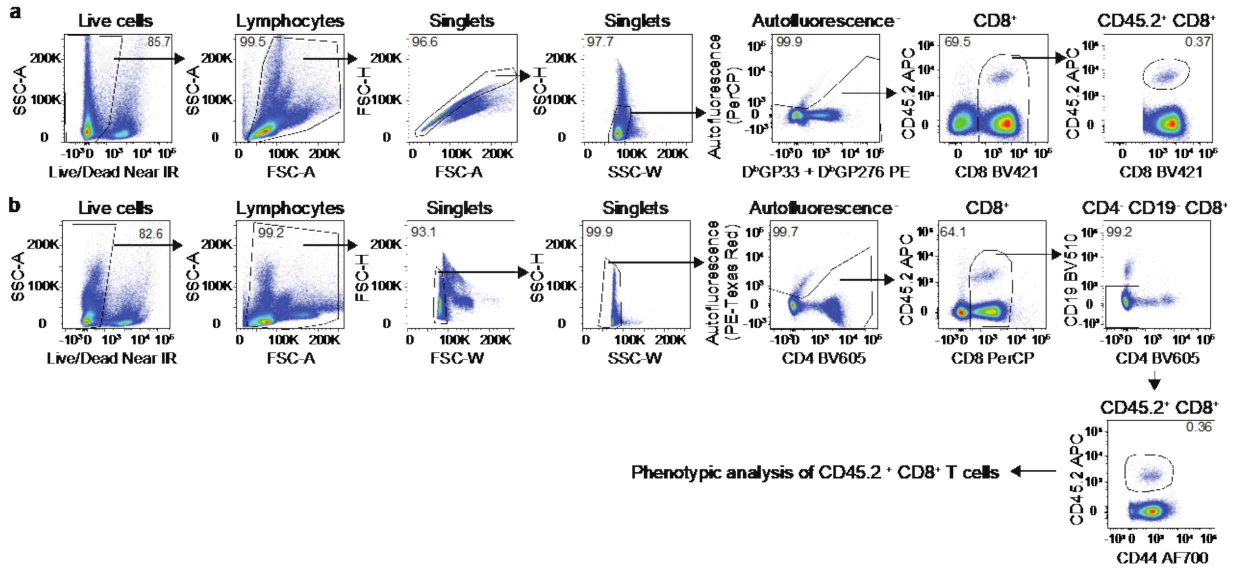

Supplementary Figure 10. Gating strategy for donor CD45.2$^+$ CD8$^+$ T cells in Fig. 2g, h and Extended Data Fig. 6f, g. Cells isolated from various tissues in chronically LCMV-infected recipient mice (CD45.1$^+$) were gated on live cells, lymphocytes, singlets, autofluorescence$^-$ cells, CD8$^+$ T cells, and donor CD45.2$^+$ CD8$^+$ T cells were analyzed for their frequency (**a**) and phenotypic marker expression (**b**). AF, Alexa Fluor.

T cell analysis on STAT5 phosphorylation and cis-trans assay

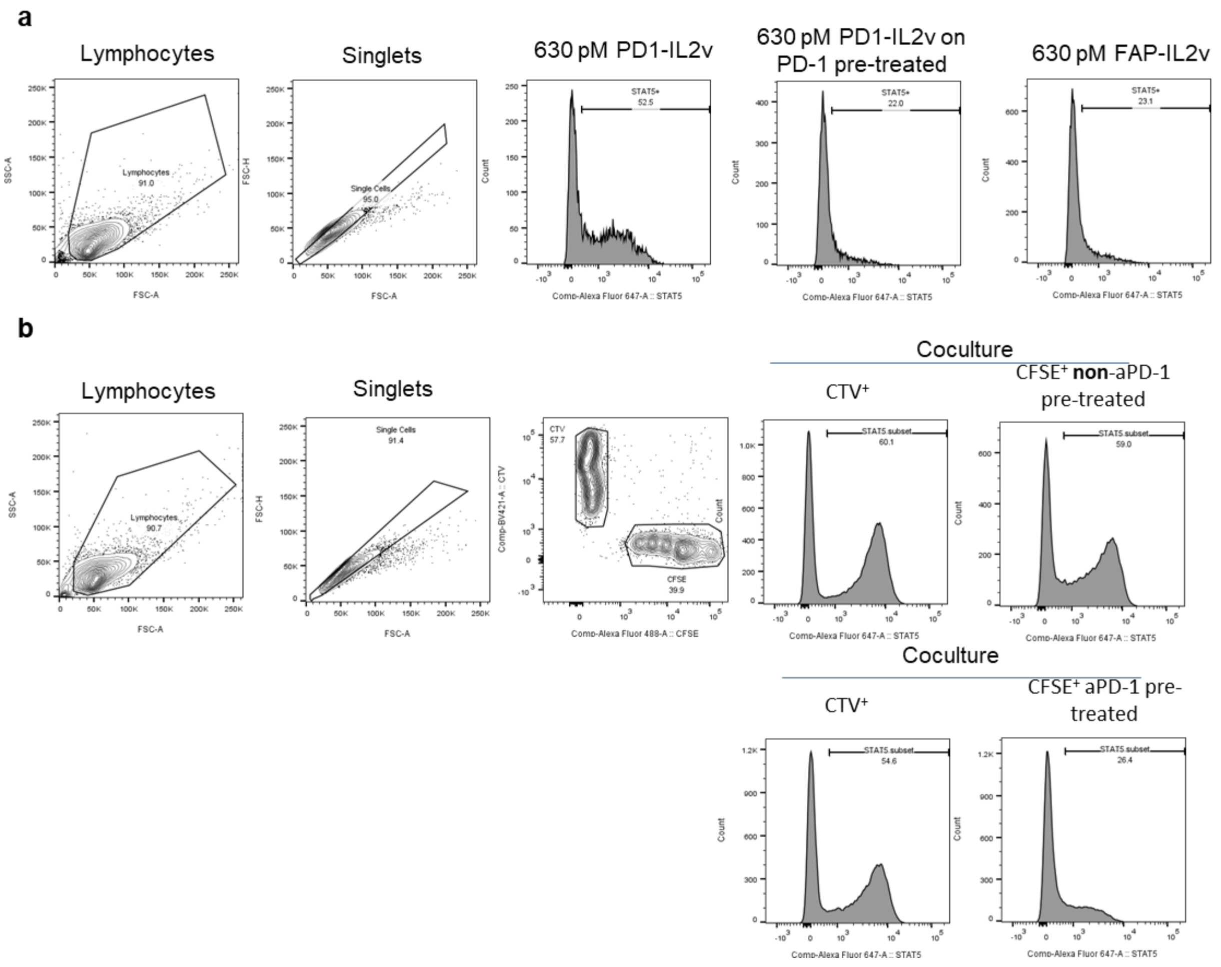

Supplementary Figure 11. Gating strategy for Fig. 1a and b. Sorted 3 days activated PD-1^{+} CD4 T cells from healthy donors were gated on lymphocytes, singlets, positivity for CFSE or CTV and measured for the frequency of STAT-5P^{+} (**a**) and (**b**).

T cell analysis on binding competition on Tregs and Tconv

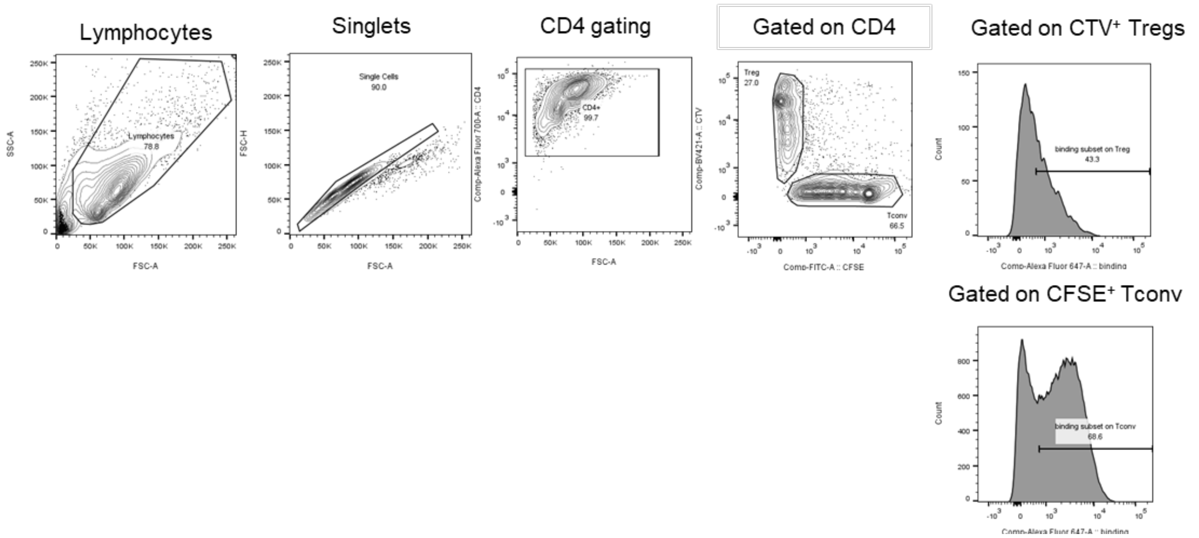

Supplementary Figure 12. Gating strategy for Fig. 1c. Sorted 3 days activated and cocultured Tregs and Tconv from healthy donors were gated on lymphocytes, singlets, $CD4^+$, $CFSE^+$ or CTV^+ and measured for the frequency of $PGLALA^+$**.**

T cell analysis on PD-1 and IL-2Rβ frequencies and quantification

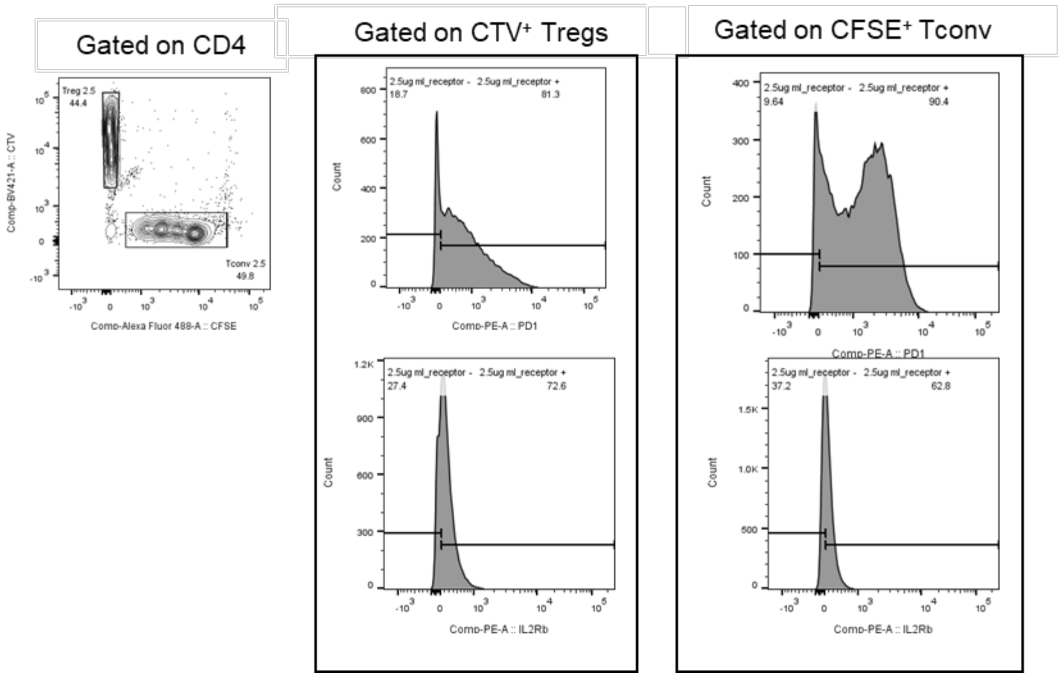

Supplementary Figure 13. Gating strategy for Fig. 1d. Sorted 3 days activated and cocultured Tregs and Tconv from healthy donors were gated on lymphocytes, singlets, $CD4^+$, $CFSE^+$ or CTV^+ and measured for the frequency and density of PD-1^+ and IL-2Rβ^+**.**

T cell analysis on Treg suppression

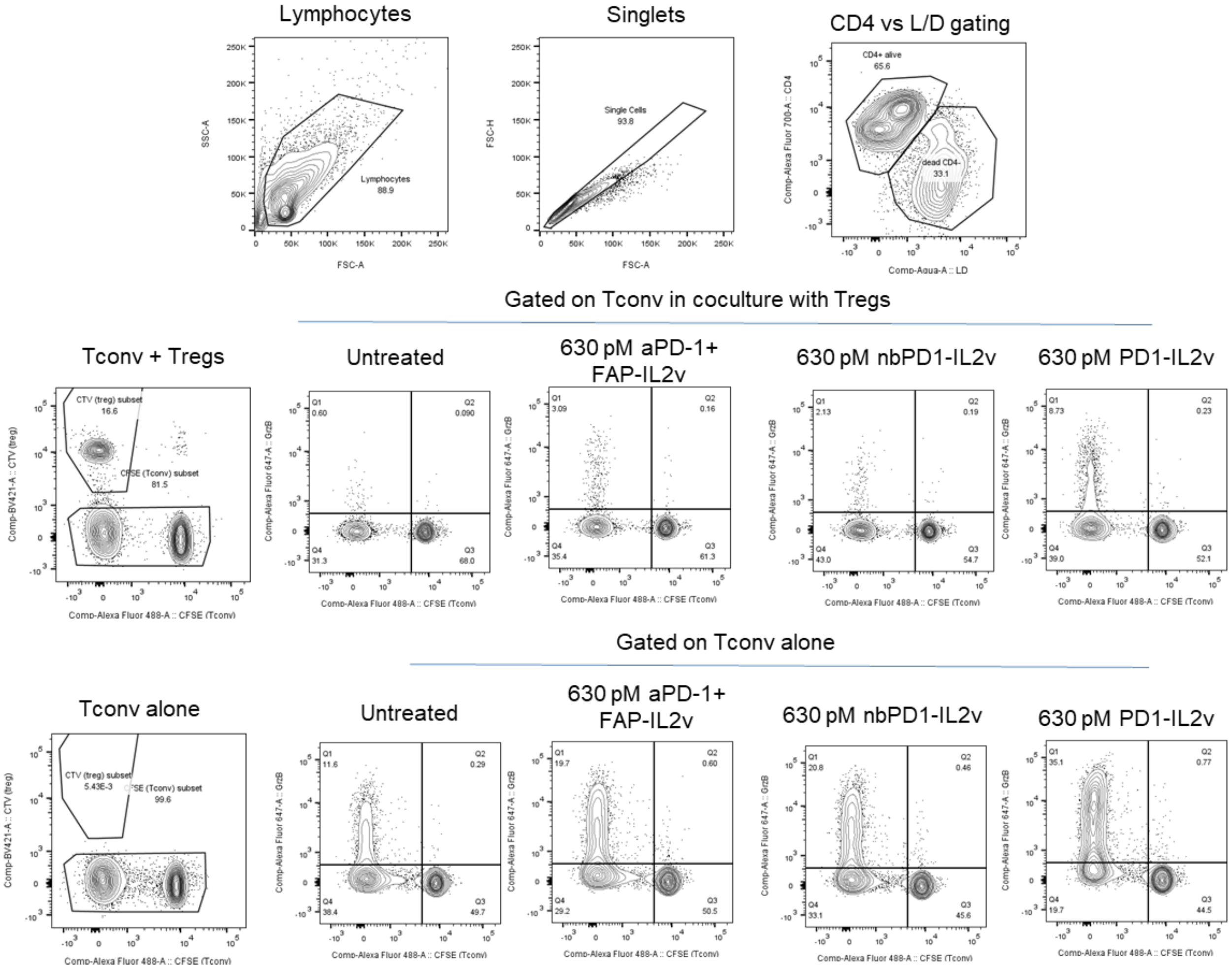

Supplementary Figure 14. Gating strategy for Fig. 1e. Sorted Tregs and Tconv from healthy donors after 5 days coculture were gated on lymphocytes, singlets, live, CD4^{+}, CFSE^{+} and measured for the frequency of granzyme B^{+}**.**

T cell analysis on GM-CSF, Granzyme B and IFN-γ measurement

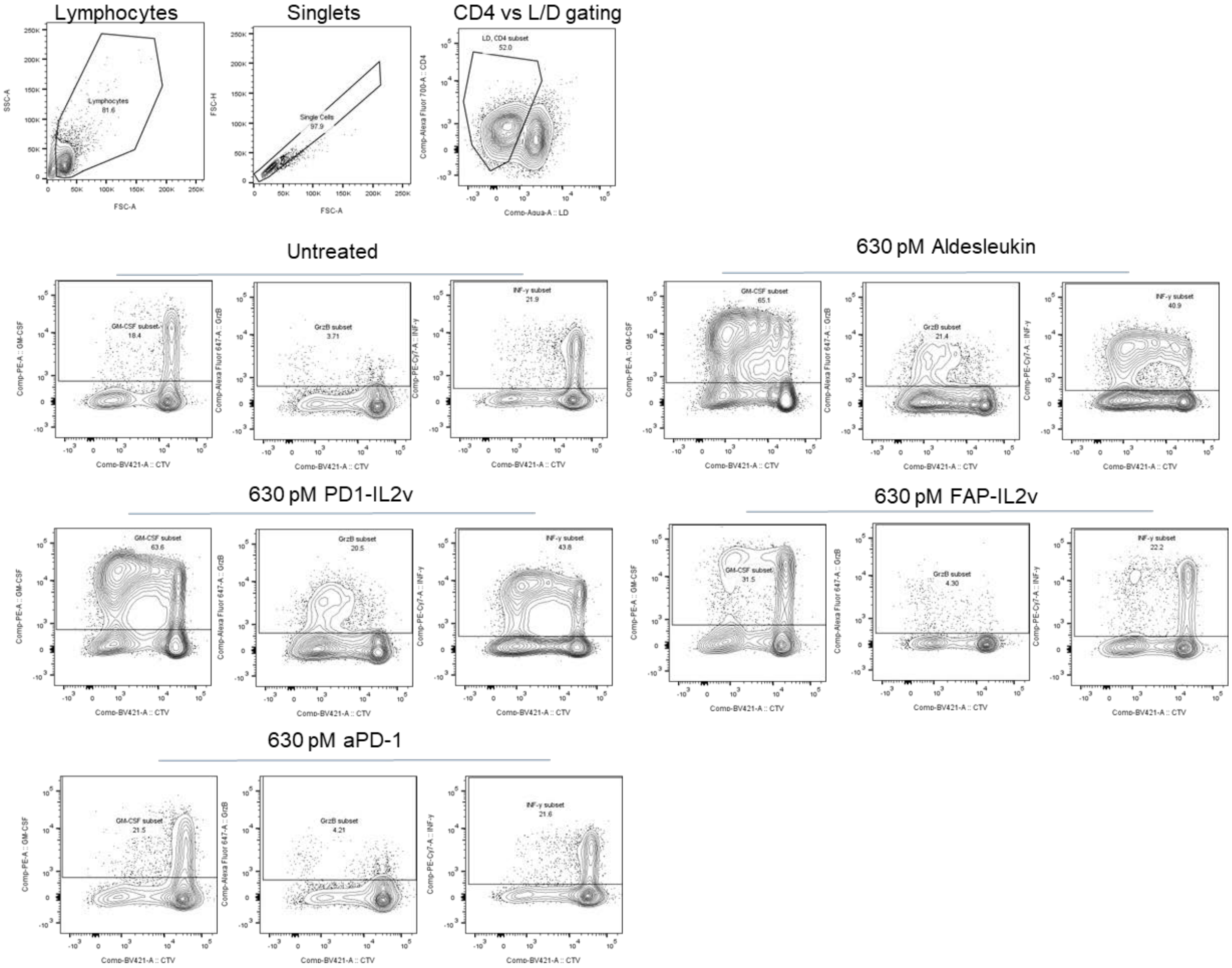

Supplementary Figure 15. Gating strategy for Fig. 1f and EDF4a. Sorted CD4 T cells from healthy donors after 5 days culture were gated on lymphocytes, singlets, live, $CD4^{+}$, $CFSE^{+}$ and measured for the frequency of GM-CSF^{+}, granzyme B^{+} and IFN-γ^{+}.

T cell analysis on CD8:CD4 T cell ratio and differentiation state in tumor and blood

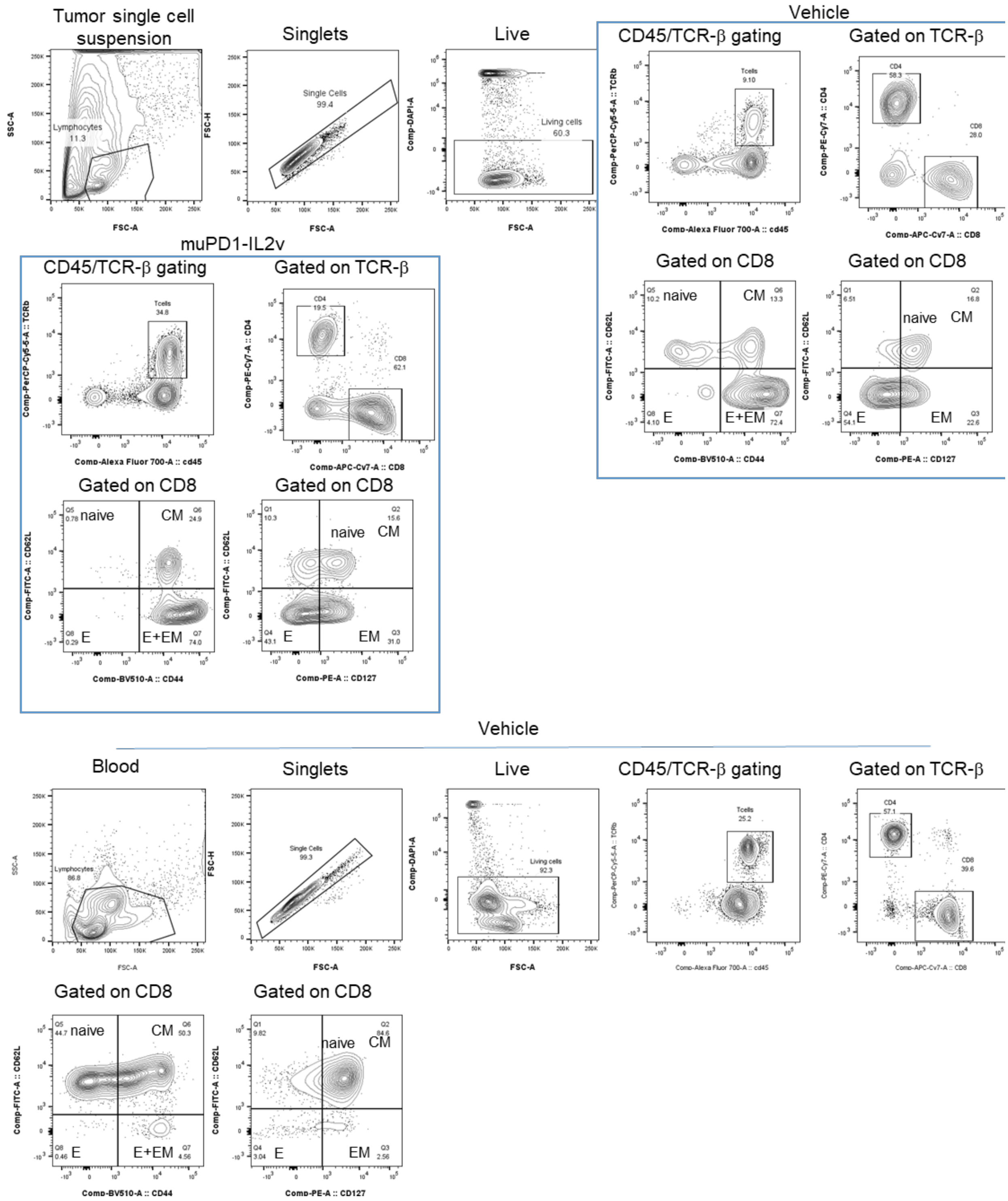

Supplementary Figure 16. Gating strategy for Fig. 3e and f. Single cell suspension of tumor and blood from syngeneic C57BL/6J mice with Panc02 tumors were gated on lymphocytes, singlets, live^{+}, CD45^{+}, TCR-β^{+}, CD8^{+} and CD4^{+}, and measured for the frequency of naïve, central memory, effector memory and effector.

T cell analysis on PD-1 and IL-2Rβ frequencies and quantification in tumor and blood

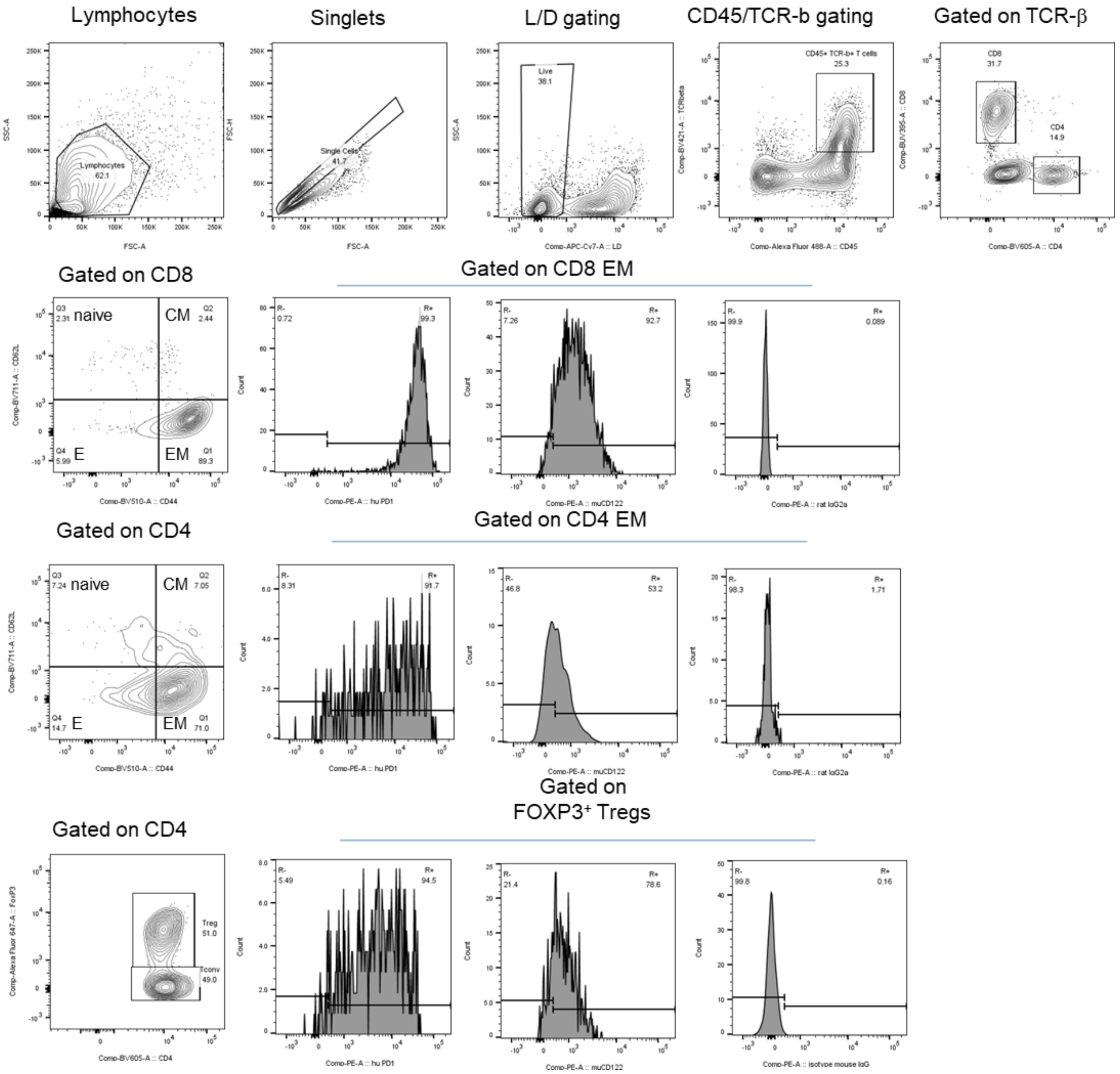

Supplementary Figure 17. Gating strategy for Fig. 3g and EDF7e and g. Single cell suspension of tumor and blood from huPD-1 transgenic mice with Panc02 tumors were gated on lymphocytes, singlets, live$^+$, CD45$^+$, TCR-β$^+$, either CD8$^+$ or CD4$^+$, and measured for the frequency and density of PD-1$^+$ and IL-2Rβ$^+$ on naïve, central memory, effector memory and effector.

T cell analysis on frequency of PD-1$^+$TCF-1$^+$ stem-like and PD-1$^+$TCF-1$^-$ CD8 TILs expressing Granzyme B and/or TIM-3

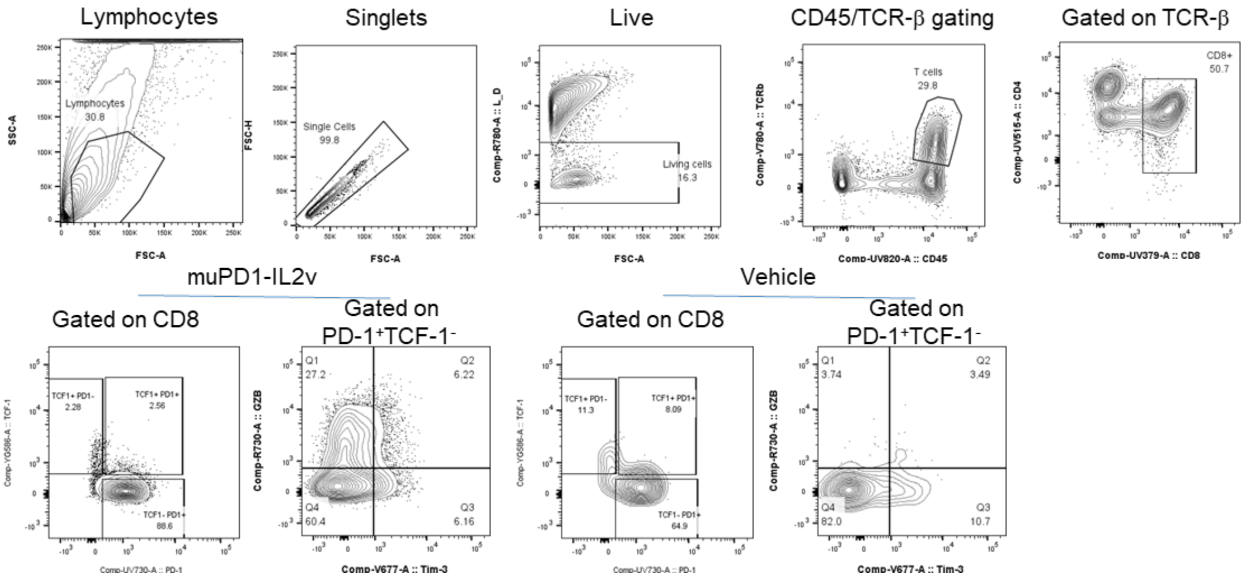

Supplementary Figure 18. Gating strategy for Fig. 4a-f. Single cell suspension of tumor from syngeneic C57BL/6J mice with Panc02 tumors were gated on lymphocytes, singlets, live$^+$, CD45$^+$, TCR-β$^+$, CD8$^+$ and measured for the frequency of PD-1 and TCF-1 expression and of Granzyme B and TIM-3

Sorting of CD8 TILs

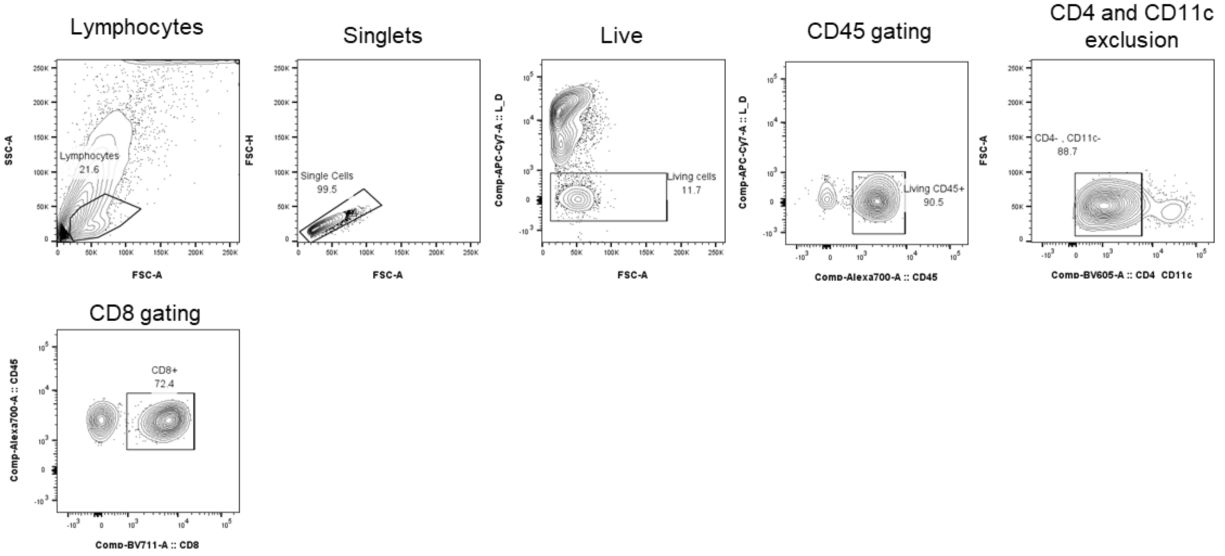

Supplementary Figure 19. Gating strategy for Fig. 4g-k. Single cell suspension of tumor from syngeneic C57BL/6J mice with Panc02 tumors were gated on lymphocytes, singlets, live^{+}, CD45^{+}, TCR-β^{+}, CD8^{+}, CD4^{-} and CD11c^{-}.

T cell analysis on frequency of OVA-specific PD-1⁺TCF-1⁺ stem-like and PD-1⁺TCF-1⁻ CD8 TILs expressing Granzyme B and/or TIM-3

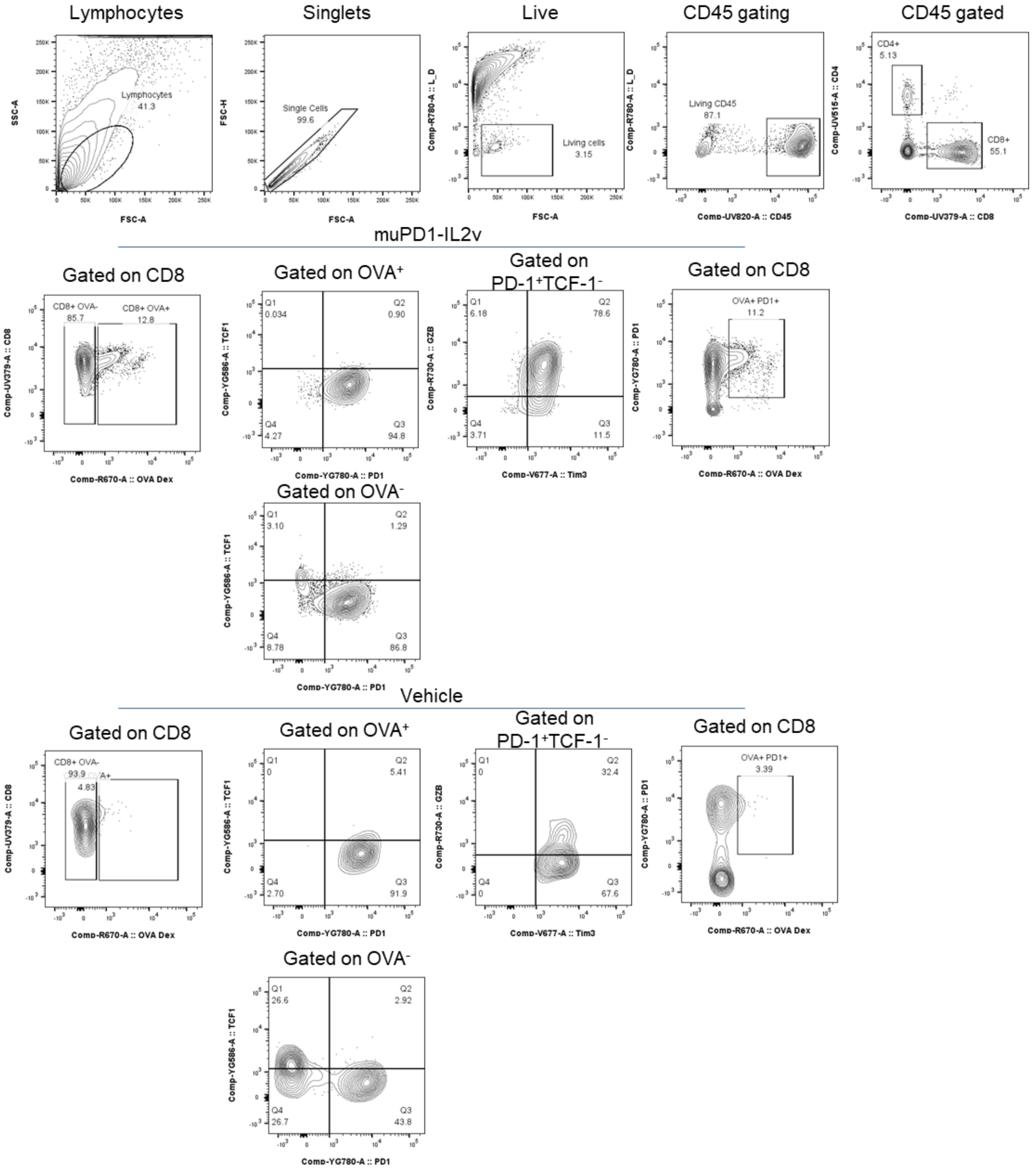

Supplementary Figure 20. Gating strategy for Fig. 5b-g. Single cell suspension of tumor from syngeneic C57BL/6J mice with Panc02 tumors were gated on lymphocytes, singlets, live⁺, CD45⁺, TCR-β⁺, CD8⁺, OVA⁺ and OVA⁻, and measured for the frequency of PD-1 and TCF-1 expression and of Granzyme B and TIM-3.

T cell analysis on restimulated CD8 TILs with OVA peptide (SIINFEKL)

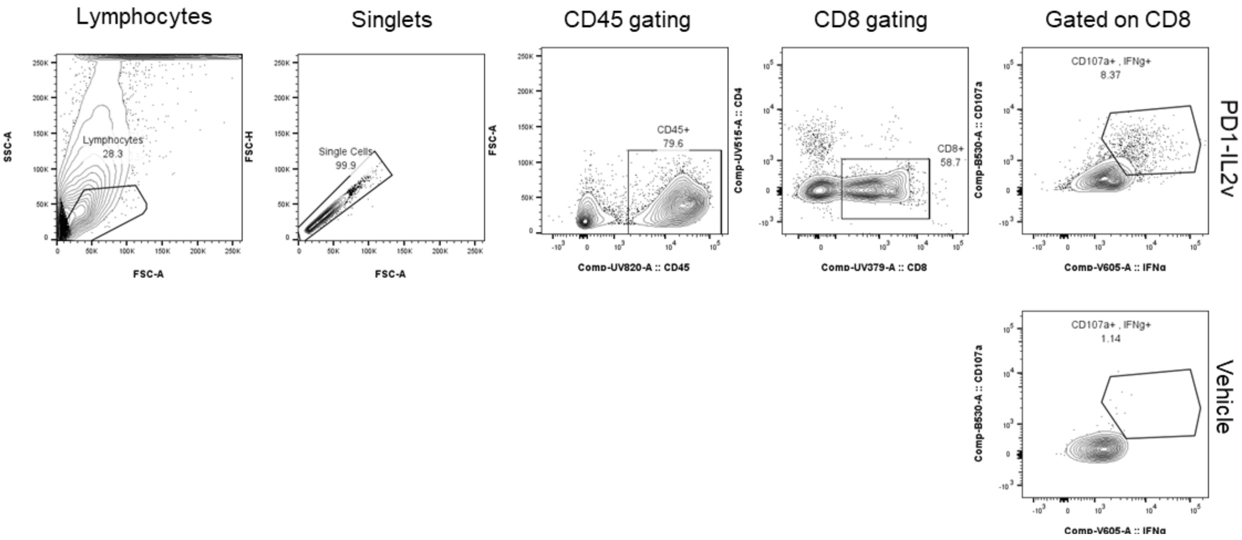

Supplementary Figure 21. Gating strategy for Fig. 5h. Single cell suspension of tumor from syngeneic C57BL/6J mice with Panc02 tumors were gated on lymphocytes, singlets, $CD45^+$, $CD8^+$, and measured for the frequency of $CD107a^+$ IFN-γ^+.

T cell analysis on STAT-5P cis-trans assay with FAP-Superkine-like

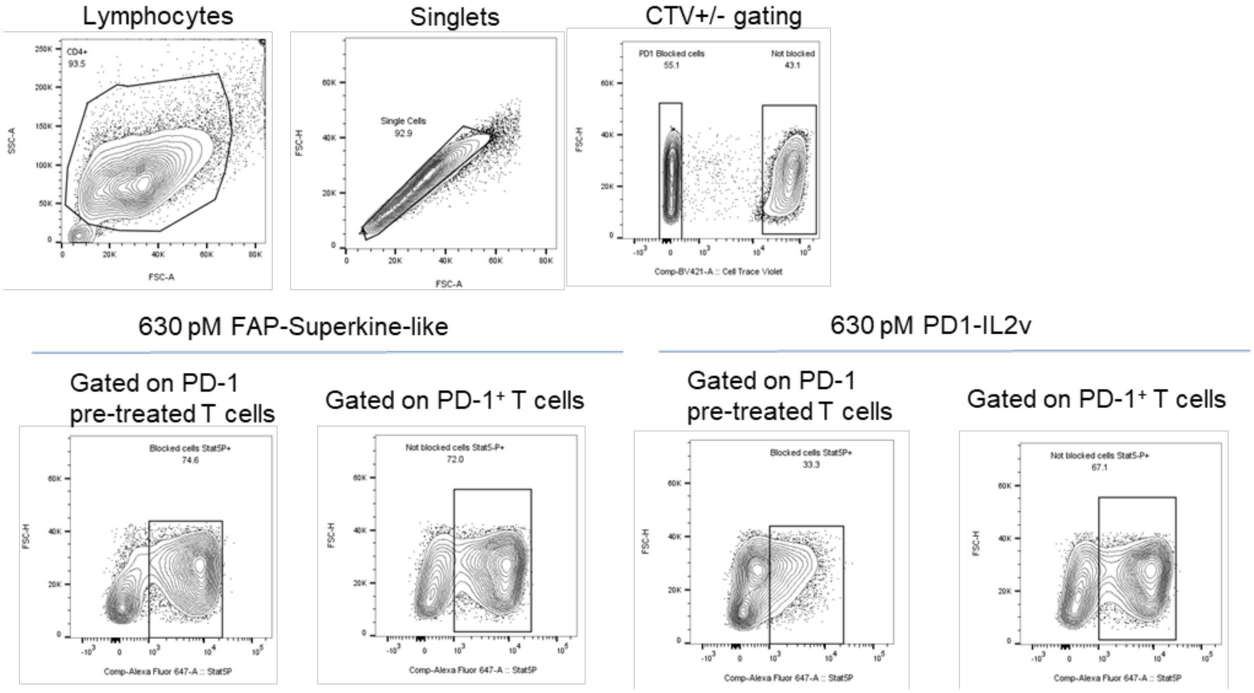

Supplementary Figure 22. Gating strategy for EDF4b. Sorted 3 days activated PD-1$^+$ CD4 T cells from healthy donors were gated on lymphocytes, singlets, positivity for CTV and measured for the frequency of STAT-5P$^+$ on either PD-1 pre-treated T cells (PD-1 deficient) or PD-1$^+$ T cells**.**

Lymphocyte analysis on preferential binding

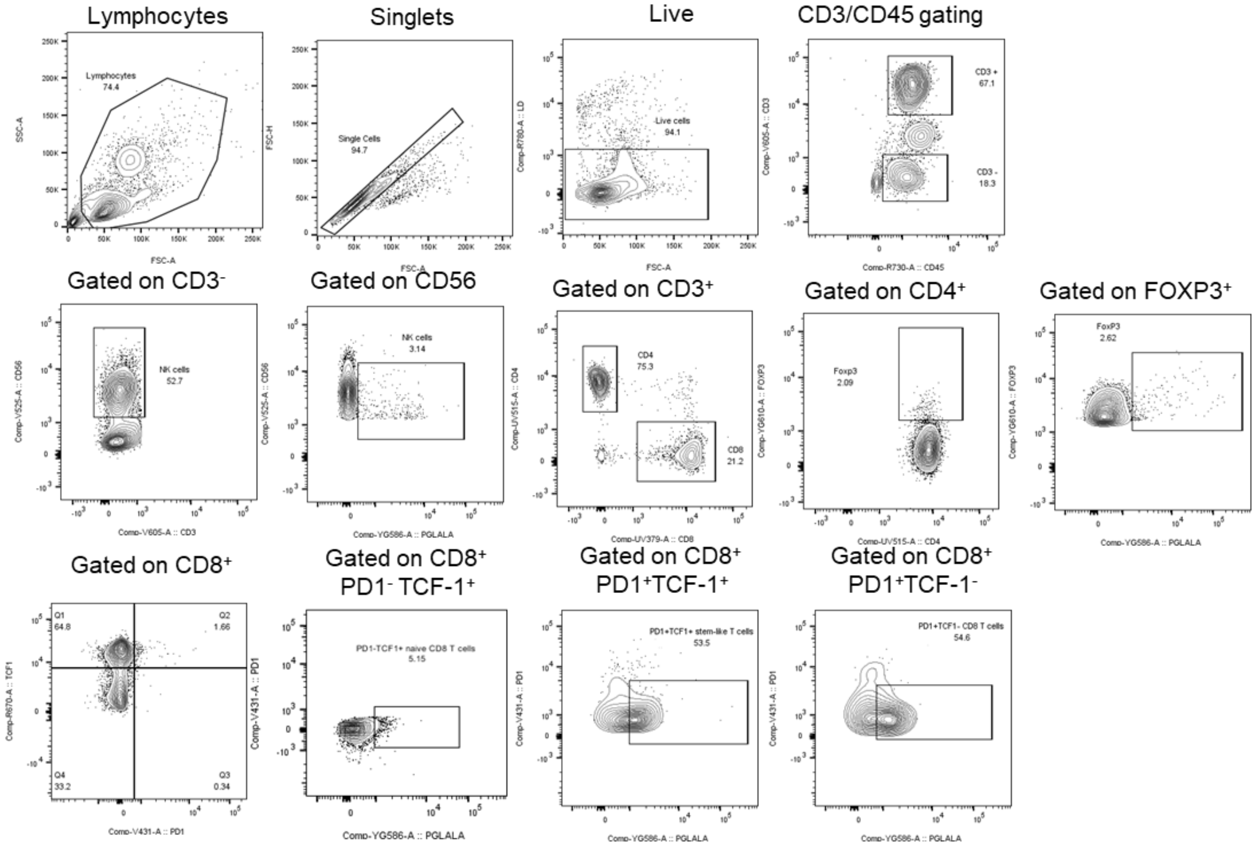

Supplementary Figure 23. Gating strategy for EDF4c. Freshly isolated PBMCs from healthy donors were gated on lymphocytes, singlets, live and either CD3$^-$ CD56$^+$**,** CD4$^+$ FOXP3$^+$, or CD8$^+$ and measured for the co-expression of PD-1 and TCF-1.

T cell analysis on cytokine secretion by CD8 TILs

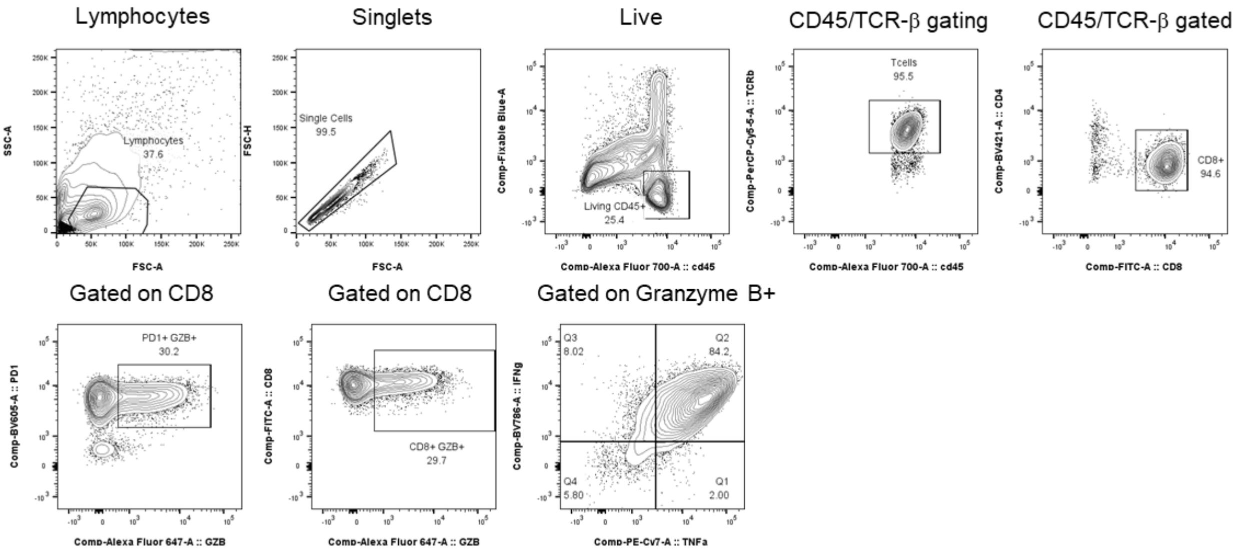

Supplementary Figure 24. Gating strategy for EDF7a-b. Single cell suspension of tumor from syngeneic C57BL/6J mice with Panc02 tumors were gated on lymphocytes, singlets, live, $CD45^+$, TCR-β^+, $CD8^+$ and measured for the frequency of PD-1^+ Granzyme B^+ and IFN-γ^+ TNF-α^+.

T cell analysis on binding competition with parental anti-PD-1 Alexa Fluor-647

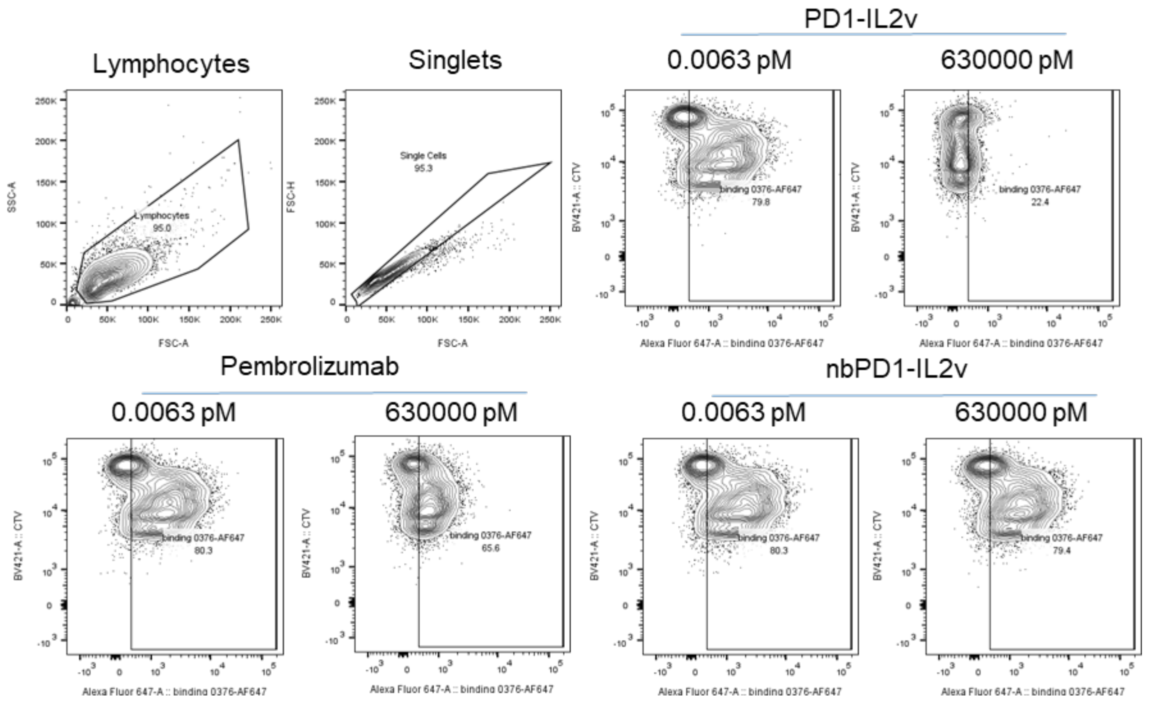

Supplementary Figure 25. Gating strategy for EDF7i. Sorted 3 days activated PD-1^{+} CD4 T cells from healthy donors were gated on lymphocytes, singlets, CTV^{+} and measured binding of parental anti-PD-1 Alexa Fluor-647**.**

Supplementary Table 1
List of antibodies used in the presented study

Ex-vivo binding on human PBMCs					
Marker	Fluorophore	Clone	#CAT	Company	Dilution/Final Concentration
Live/Dead	APC-Cy7		65-0865-14	eBioscience	1:500
CD45	AF700	HI30	56-9459-42	eBioscience	1:100
CD3	BV605	OKT3	317322	Biolegend	1:100
CD4	BUV496	OKT4	612936	BD Biosciences	1:100
CD8	BUV395	RPA-T8	563795	BD Biosciences	1:100
CD366 (TIM-3)	BV711	F38-2E2	345024	Biolegend	1:20
LAG3 (CD223)	PerCP-Cy5.5	3DS223H	46-2239-42	eBioscience	1:20
TIGIT	BV786	741182	747838	BD Biosciences	1:20
CD218a (IL-18Ra)	FITC	H44	313810	Biolegend	1:100
CD56	BV5480	NCAM16.2	566124	BD Biosciences	1:20
TCR g/d	PE-Cy7	B1	331222	Biolegend	1:50
PG-LALA	PE	NA		Roche	1:650 (2.5ug/ml)
TCF-1 /TCF-7	AF647	C63D9	6709S	Cell Signaling Technologie	1:100
PD-1 IC		D4W2J	86163S	Cell Signaling Technologie	1:100
Goat anti-Rabbit secondary ab	BV421	Polyclonal	565014	BD	1:100
FOXP3	PE-CF594	206D	320126	Biolegend	1:50
In-vitro experiment on human CD4 T cells (incl. T con and Tregs)					
Marker	Fluorophore	Clone	#CAT	Company	Dilution/Final Concentration
Stat5 (pY694)	AF647	47/Stat5 (pY694)	562076	BD	1:20
PD-1	PE	EH12.2H7	329906	Biolegend	2.5ug/ml
IL2Rb	PE	TU27	339006	Biolegend	2.5ug/ml
isotype	PE	MOPC-21	400112	Biolegend	2.5ug/ml
Parental PD-1	AF647	0376	NA	Roche	1ug/ml
PD1-IL2v	AF647	0376-IL2v fused	NA	Roche	1ug/ml
CD4	AF700	RPA-T4	56-0049-42	eBioscience	1:50
live/dead	Aqua Dead Cell Stain	-	L34966	Invitrogen	1:1000
Granzyme B	AF647	GB11	561999	BD Biosciences	1:100
GM-CSF	PE	BVD2-21C11	502306	Biolegend	1:100
IFNγ	PE-Cy7	4S.B3	25-7319-82	eBioscience	1:200
Ex-vivo receptor quantification on mouse TILs and T cells from blood					

Marker	Fluorophore	Clone	#CAT	Company	Dilution/Final Concentration
PD-1	PE	29F.1A12	135206	Biolegend	2.5ug/ml
PD-1	PE	EH12.2H7	329906	Biolegend	2.5ug/ml
IL2Rb	PE	5H4	105906	Biolegend	2.5ug/ml
TCRb	BV421	H57-597	109230	Biolegend	1:200
CD8	BV395	53-6.7	565968	BD Biosciences	1:200
CD4	AF488	GK1.5	100406	Biolegend	1:100
CD62L	BV711	MEL-14	104445	Biolegend	1:200
CD44	BV480	IM7	566116	BD Biosciences	1:200
FOXP3	AF647	150D	320014	Biolegend	1:100
Antibodies used for the LCMV-chronic infection					
Marker	Fluorophore	Clone	#CAT	Company	Dilution
CD4	BUV563	RM4-5	741217	BD Biosciences	1:500
CD4	FITC	RM4-5	553046	BD Biosciences	1:500
CD4	V500	RM4-5	560782	Biolegend	1:500
CD4	BV605	RM4-5	100548	Biolegend	1:500
CD4	BV711	RM4-5	100557	Biolegend	1:500
CD4	PE-Cy7	RM4-5	25-0042-82	Thermo Fischer Scientific	1:500
CD4	APC-eFluor 780	RM4-5	47-0042-82	Thermo Fischer Scientific	1:500
CD8a	BUV496	53-6.7	563786	BD Biosciences	1:100
CD8a	BV421	53-6.7	100753	Biolegend	1:150
CD8a	BV605	53-6.7	100744	Biolegend	1:100
CD8a	PerCP	53-6.7	553036	BD Biosciences	1:100
CD19	BUV563	1D3	749028	BD Biosciences	1:150
CD19	BV510	1D3	115546	Biolegend	1:150
CD19	BV605	1D3	115540	Biolegend	1:150
CD19	PE-Cy7	1D3	25-0193-82	Thermo Fischer Scientific	1:150
CD19	APC-eFluor 780	1D3	47-0193-82	Thermo Fischer Scientific	1:150
CD44	BUV805	IM7	741921	BD Biosciences	1:500
CD44	FITC	IM7	561859	BD Biosciences	1:500
CD44	AF700	IM7	56-0441-82	Thermo Fischer Scientific	1:100
CD45.2	APC	104	109814	Biolegend	1:100
CD127	BUV737	SB/199	612841	BD Biosciences	1:100
CD127	PE	A7R34	12-1271-83	Thermo Fischer Scientific	1:100
CD218a	PE	P3TUNYA	12-5183-82	Thermo Fischer Scientific	1:100
CD218a	PE-Cy7	P3TUNYA	25-5183-82	Thermo Fischer Scientific	1:100

CXCR3	PE-Cy7	CXCR3-173	25-1831-82	Thermo Fischer Scientific	1:100
CXCR5	BV421	L138D7	145512	Biolegend	1:50
CX3CR1	BV785	SA011F11	149031	Biolegend	1:500
Foxp3	PE-Cy7	FJK-16s	25-5773-82	Thermo Fischer Scientific	1:250
IL-2	PE	JES6-5H4	554428	BD Biosciences	1:100
IFN-γ	BV421	XMG1.2	505830	Biolegend	1:100
IFN-γ	APC	XMG1.2	554413	BD Biosciences	1:100
PD-1	PE	RMP1-30	109104	Biolegend	1:100
PD-1	APC	RMP1-30	109112	Biolegend	1:100
TCF-1	PE	S33-966	564217	BD Biosciences	1:100
Tim-3	BUV395	5D12	747620	BD Biosciences	1:100
Tim-3	AF488	215008	FAB1529G	R&D systems	1:20
TNF-a	BV421	MP6-XT22	506328	Biolegend	1:100
TNF-a	PE	MP6-XT22	554419	BD Biosciences	1:100
Name		Clone	#Catalogue		
anti-mouse PD-L1 with DAPG mutation		NA	NA	Roche	200 µg/mouse/injection
mouse IgG1 isotype control		MOPC-21	BE0083	BioXcell	200 µg/mouse/injection
anti-mouse CD4		GK1.5	BE0003-1	BioXcell	300 µg/mouse/injection
Antibodies used for TILs characterization in mouse tumor-model					
Marker	Fluorochrome	clone	Cat number	Provider	Dilution
Fixable Viability Dye	eFluor™ 455UV		65-0868-14		1:500
CD45	AF700	30-F11	103128	Biolegend	1:300
TCRb	PercP-Cy5.5	H57-597	109228	Biolegend	1:200
CD8	APC-Cy7	53-6.7	100714	Biolegend	1:200
CD4	PE-Cy7	GK1.5	100422	Biolegend	1:200
CD62L	FITC	MEL-14	104406	Biolegend	1:200
CD127	PE	A7R34	135010	Biolegend	1:100
TCRb	PerCP-Cy5.5	H57-597	109228	Biolegend	1:200
CD4	BV421	GK1.5	100438	Biolegend	1:200
Granzyme B	AF647	GB11	515406	Biolegend	1:100
IFN-g	BV786	XMG1.2	505838	Biolegend	1:100
TNFa	PE-Cy7	MP6-XT22	506306	Biolegend	1:100
FoxP3	BV421	MF-14	126419	Biolegend	1:100
CD39	AF647	Duha59	143808	Biolegend	1:200

Granzyme B	AF700	QA16A02	372222	Biolegend	1:100
ki67	PE-Cy7	16A8	652426	Biolegend	1:300
PD1	PE-Cy7	RMP1-30	109110	Biolegend	1:200
CD25	BV711	RMT3-23	102049	Biolegend	1:200
TIGIT	PE-Dazzle594	1G9	142110	Biolegend	1:100
IFN-g	BV605	XMG1.2	505840	Biolegend	1:100
TNFa	BV421	MP6-XT22	506328	Biolegend	1:100
CD107a	AF488	1D4B	121608	Biolegend	1:100
CD44	BV510	IM7	563114	BD Biosciences	1:200
CD45	BUV805	30-F11	BDB748370	BD Biosciences	1:100
TCRb	BV786	H57-597	742484	BD Biosciences	1:100
CD4	BUV496	RM4-5	612952	BD Biosciences	1:100
CD8	BUV395	53-6.7	563786	BD Biosciences	1:100
PD-1	BUV737	RMP1-30	749306	BD Biosciences	1:100
CD25	PE-CF594	PC61	562694	BD Biosciences	1:100
Tim3	BV650	5D12	747623	BD Biosciences	1:100
TCF1	PE	S33-966	564217	BD Biosciences	1:100
LAG3	BV650	C9B7W	740560	BD Biosciences	1:100
SLAMF6	BV510	13G3	745073	BD Biosciences	1:50
CD218a	FITC	REA947	130-115-703	Miltenyi	1:50
OVA-dextramer H-2 Kb ((SIINFEKL)	APC		JD2163	Immudex	1:100
Rat anti-mouse CD8α InVivoPlus		2.43	BP0061	BioXcell	100 µg/mouse/injection
Feature barcoding					
Marker	Oligo Tag	Clone	Source	Concentration (1 µg/ml)	
CD28	ATTAAGAGCGTGTTG	37.51	TotalSeq-C, BioLegend	1	
CD44	TGGCTTCAGGTCCTA	IM7	TotalSeq-C, BioLegend	1	
CD62L (L-selectin)	TGGGCCTAAGTCATC	MEL-14	TotalSeq-C, BioLegend	1	
CD39	GCGTATTTAACCCGT	Duha59	TotalSeq-C, BioLegend	1	
CD279 (PD-1)	GAAAGTCAAAGCACT	RMP1-30	TotalSeq-C, BioLegend	1	
CD366 (Tim-3)	ATTGGCACTCAGATG	RMT3-23	TotalSeq-C, BioLegend	1	

CD223 (LAG-3)	ATTCCGTCCCTAAGG	C9B7W	TotalSeq-C, BioLegend	1	
CD183 (CXCR3)	GTTCACGCCGTGTTA	CXCR3-173	TotalSeq-C, BioLegend	1	
CD185 (CXCR5)	ACGTAGTCACCTAGT	L138D7	TotalSeq-C, BioLegend	1	
CD127 (IL-7Rα)	GTGTGAGGCACTCTT	A7R34	TotalSeq-C, BioLegend	1	
TIGIT (Vstm3)	GAAAGTCGCCAACAG	1G9	TotalSeq-C, BioLegend	1	
CD25	ACCATGAGACACAGT	PC61	TotalSeq-C, BioLegend	1	
Ly108 (SLAM-F6)	CGATTCTTTGCGAGT	330-AJ	TotalSeq-C, BioLegend	1	
CD137 (4-1BB)	TCCCTGTATAGATGA	17B5	TotalSeq-C, BioLegend	1	
IL-21R	GATTCCGACAGTAGA	4A9	TotalSeq-C, BioLegend	1	
Histology					
Marker	Fluorochrome	clone	Cat number	Provider	Dilution
CD3		SP7	RMAB005	Diagnostic Biosystems	1:100
CD8		4SM15	14-0808-82	eBioscience	1:300
PD-1		Polyclonal	AF1021	R&D Systems	1:250
Granzyme B		Polyclonal	Ab4059	Abcam	1:250
Confocal Imaging					
Marker	Fluorochrome	clone	Cat number	Provider	Dilution
PD-1 IC		D4W2J	86163S	Cell Signaling Technologie	1:100
Goat anti-rabbit IgG (H+L), F(ab')2 Fragment	AF488	Polyclonal	4412S	Cell Signaling Technologie	1:1000
Parental PD-1	Pure	0376	NA	Roche	10ug/ml
PD1-IL2v	AF647	0376-IL2v fused	NA	Roche	1ug/ml
FAP-IL2v	AF647	4B9-IL2v fused	NA	Roche	1ug/ml
Cell sorting					
Marker	Fluorochrome	clone	Cat number	Provider	Dilution
CD45	AF700	30-F11	103128	Biolegend	1:100

CD8	BV711	53-6.7	100748	Biolegend	1:100
CD4	BV605 (Bin channel)	GK1.5	100451	Biolegend	1:100
CD11c	BV605 (Bin channel)	N418	117334	Biolegend	1:100
Live/Dead	APC-Cy7		65-0865-14	eBiosciences	1:500

Supplementary Notes

Acknowledgements

For generating RNA-seq data from chronic infection experiments, we thank Emory University School of Medicine Flow Cytometry Core (K. Fife and R. Karaffa), Yerkes NPRC Genomics Core (K. Pellegrini and S. Bosinger, NIH P51OD011132 and NIH S10 OD026799), and Emory Integrated Genomics Core (EIGC) Shared Resource of Winship Cancer Institute of Emory University and NIH/NCI (L. Griffiths, 2P30CA138292-04).
