# Supplementary material for: PD-1-cis IL-2R agonism yields better effectors from stem-like CD8+ T cells
Source: Nature. 2022 Sep 28;610(7930):161–72. doi: 10.1038/s41586-022-05192-0 (PMC9534752; doi:10.1038/s41586-022-05192-0)
Supplement: Supplementary file 2 — Reporting Summary [file 41586_2022_5192_MOESM2_ESM.pdf]

nature portfolio

Corresponding author(s): Christian Klein and Pablo Umana

Last updated by author(s): Jul 20, 2022

Reporting Summary

Nature Portfolio wishes to improve the reproducibility of the work that we publish. This form provides structure for consistency and transparency in reporting. For further information on Nature Portfolio policies, see our Editorial Policies and the Editorial Policy Checklist.

Statistics

For all statistical analyses, confirm that the following items are present in the figure legend, table legend, main text, or Methods section.

n/a	Confirmed	
☐	☒	The exact sample size (*n*) for each experimental group/condition, given as a discrete number and unit of measurement
☐	☒	A statement on whether measurements were taken from distinct samples or whether the same sample was measured repeatedly
☐	☒	The statistical test(s) used AND whether they are one- or two-sided<br>*Only common tests should be described solely by name; describe more complex techniques in the Methods section.*
☒	☐	A description of all covariates tested
☐	☒	A description of any assumptions or corrections, such as tests of normality and adjustment for multiple comparisons
☐	☒	A full description of the statistical parameters including central tendency (e.g. means) or other basic estimates (e.g. regression coefficient) AND variation (e.g. standard deviation) or associated estimates of uncertainty (e.g. confidence intervals)
☐	☒	For null hypothesis testing, the test statistic (e.g. *F*, *t*, *r*) with confidence intervals, effect sizes, degrees of freedom and *P* value noted<br>*Give P values as exact values whenever suitable.*
☒	☐	For Bayesian analysis, information on the choice of priors and Markov chain Monte Carlo settings
☒	☐	For hierarchical and complex designs, identification of the appropriate level for tests and full reporting of outcomes
☒	☐	Estimates of effect sizes (e.g. Cohen's *d*, Pearson's *r*), indicating how they were calculated

Our web collection on statistics for biologists contains articles on many of the points above.

Software and code

Policy information about availability of computer code

Data collection	FACSDiva (BD Biosciences), Olympus VS120-L100 Virtual Slide Microscope scanner, Ilumina NovaSeq 6000, 10X Genomics, confocal microscope (Leica Sp8)
Data analysis	FACS data: FlowJo v. 9.9.6 or 10.8.1(BD Biosciences), GraphPad Prism v.8 or v.9.3.1, Microsoft Excel 14.7.7.<br>RNA-seq analysis:R Studio v.1.3.1093, HISAT2 v.2.1.0, featureCounts v.1.5.2, DESeq2 v.1.24.0, ggplot2 v.3.3.2, ComplexHeatmap R package (v 2.2.0).<br>Single Cell RNA-seq:CellRanger v3.1 or v.6.0.0, Seurat v.3.0, VISION R package v.1.1.0. scanpy 1.6.0 and the besca 2.4 standard workflow<br>TCR analysis was performed in Python with the toolkit scirpy 0.7.1<br>Confocal images: Imaris 9.5.1 (Bitplane), Matlab 2020a (Mathworks)

For manuscripts utilizing custom algorithms or software that are central to the research but not yet described in published literature, software must be made available to editors and reviewers. We strongly encourage code deposition in a community repository (e.g. GitHub). See the Nature Portfolio guidelines for submitting code & software for further information.

Data

Policy information about availability of data

All manuscripts must include a data availability statement. This statement should provide the following information, where applicable:

- Accession codes, unique identifiers, or web links for publicly available datasets
- A description of any restrictions on data availability
- For clinical datasets or third party data, please ensure that the statement adheres to our policy

The scRNA-seq., TCRseq. and CITEseq. data on tumor infiltrating lymphocytes discussed in this publication have been deposited in ArrayExpress with the accession number E-MTAB-11773.
RNA-seq data from chronic infection experiments are available in the NCBI Gene Expression Omnibus (GEO) database under the accession number GSE208556. Reads were mapped to the GRCm38/mm10 genome53 with HISAT2 version 2.1.054. Gene expression was quantifed by featureCounts55 (v.1.5.2). DESeq2 (Love et al., 2014, v.1.24.0) was used to normalized for library size and calculate differential expression across groups.

Human research participants

Policy information about studies involving human research participants and Sex and Gender in Research.

Reporting on sex and gender	Sex: 39 females and 11 males
Population characteristics	Healthy donors, born between 1949-2001
Recruitment	Healthy donors that donate blood on volunteer basis at the blood donation center (Blutspende Zürich, Switzerland, https://www.blutspendezurich.ch/) randomly allocated to Roche
Ethics oversight	Approval of the Cantonal Ethics Committee (Zürich)

Note that full information on the approval of the study protocol must also be provided in the manuscript.

Field-specific reporting

Please select the one below that is the best fit for your research. If you are not sure, read the appropriate sections before making your selection.

☒ Life sciences ☐ Behavioural & social sciences ☐ Ecological, evolutionary & environmental sciences

For a reference copy of the document with all sections, see nature.com/documents/nr-reporting-summary-flat.pdf

Life sciences study design

All studies must disclose on these points even when the disclosure is negative.

Sample size	For the chronic infection experiments: No statistical methods were used to predetermine sample size. Sample sizes were chosen based on previous experiences (West E. E. et al, PD-L1 blockade synergizes with IL-2 therapy in reinvigorating exhausted T cells. J Clin Invest 123, 2604-2615, doi:10.1172/JCI67008 (2013).), balancing statistical robustness and animal welfare.<br>For the cancer model experiments: group size is determine statistically using JMP statistic software program to allow significant difference with minimun amount of mice per group to comply with country animal welfare guidelines.<br>For in-vitro and ex-vivo experiments 3 to 6 different donors from 3 to 6 independent experiments were used based on inter-donor variability, data homogeneity and statistical significance.<br>For imaging studies at least 12 cells were analyzed from the selected optical section in order to have statistical significance.
Data exclusions	For scRNAseq on TILs: two samples were excluded because of low overall quality and very low cell number, respectively, all other samples were included in the analysis.
Replication	All data were reliably reproduced. The number of repeats and sample sizes are provided in each figure legend.
Randomization	For chronic infection model: LCMV chronically infected mice were randomly assigned to experimental groups.<br>For mouse tumor models: Randomization is performed with the use of an automated software in the POMES platform. Group size is determine statistically to allow significant difference with minimun amount of mice per group to comply with country animal welfare guidelines.<br>For in-vitro studies all used donors were either left untreated (negative control) or were exposed in parallel to equimolar concentrations of all the indicated therapies.
Blinding	For the chronic infection model: investigators were not blinded to group allocation during experimental setup, data collection, and analysis. No blinding was performed since we did not have the personnel resources to consistently perform blinding.<br>For the mouse tumor models: investigators were blinded to group allocation during data collection and analysis by means of assigning letters to the treatment groups by people involved in preparing the drug dilutions that need to be injected. Technical involved personnel are

randomly assigned to studies during data collection and unaware of the hyphotesis/expected outcome of treatment groups.
For in-vitro and ex-vivo studies there was no blinding but the data were reproduced by different technical personnel.

Reporting for specific materials, systems and methods

We require information from authors about some types of materials, experimental systems and methods used in many studies. Here, indicate whether each material, system or method listed is relevant to your study. If you are not sure if a list item applies to your research, read the appropriate section before selecting a response.

Materials & experimental systems

n/a	Involved in the study
☐	☒ Antibodies
☐	☒ Eukaryotic cell lines
☒	☐ Palaeontology and archaeology
☐	☒ Animals and other organisms
☒	☐ Clinical data
☒	☐ Dual use research of concern

Methods

n/a	Involved in the study
☒	☐ ChIP-seq
☐	☒ Flow cytometry
☒	☐ MRI-based neuroimaging

Antibodies

Antibodies used

The complete antibody list is provided as supplementary information.
Ex-vivo binding on human PBMCs
Marker Fluorophore Clone #CAT Company Dilution/Final Concentration
Live/Dead APC-Cy7 65-0865-14 eBioscience 1:500
CD45 AF700 HI30 56-9459-42 eBioscience 1:100
CD3 BV605 OKT3 317322 Biolegend 1:100
CD4 BUV496 OKT4 612936 BD Biosciences 1:100
CD8 BUV395 RPA-T8 563795 BD Biosciences 1:100
CD366 (TIM-3) BV711 F38-2E2 345024 Biolegend 1:20
LAG3 (CD223) PerCP-Cy5.5 3DS223H 46-2239-42 eBioscience 1:20
TIGIT BV786 741182 747838 BD Biosciences 1:20
CD218a (IL-18Ra) FITC H44 313810 Biolegend 1:100
CD56 BV5480 NCAM16.2 566124 BD Biosciences 1:20
TCR g/d PE-Cy7 B1 331222 Biolegend 1:50
PG-LALA PE NA Roche 1:650 (2.5ug/ml)
TCF-1 /TCF-7 AF647 C63D9 6709S Cell Signaling Technologie 1:100
PD-1 IC D4W2J 86163S Cell Signaling Technologie 1:100
Goat anti-Rabbit secondary ab BV421 Polyclonal 565014 BD 1:100
FOXP3 PE-CF594 206D 320126 Biolegend 1:50
In-vitro experiment on human CD4 T cells (incl. T con and Tregs)
Marker Fluorophore Clone #CAT Company Dilution/Final Concentration
Stat5 (pY694) AF647 47/Stat5 (pY694) 562076 BD 1:20
PD-1 PE EH12.2H7 329906 Biolegend 2.5ug/ml
IL2Rb PE TU27 339006 Biolegend 2.5ug/ml
isotype PE MOPC-21 400112 Biolegend 2.5ug/ml
Parental PD-1 AF647 0376 NA Roche 1ug/ml
PD1-IL2v AF647 0376-IL2v fused NA Roche 1ug/ml
CD4 AF700 RPA-T4 56-0049-42 eBioscience 1:50
live/dead Aqua Dead Cell Stain - L34966 Invitrogen 1:1000
Granzyme B AF647 GB11 561999 BD Biosciences 1:100
GM-CSF PE BVD2-21C11 502306 Biolegend 1:100
IFNγ PE-Cy7 4S.B3 25-7319-82 eBioscience 1:200

Ex-vivo receptor quantification on mouse TILs and T cells from blood
Marker Fluorophore Clone #CAT Company Dilution/Final Concentration
PD-1 PE 29F.1A12 135206 Biolegend 2.5ug/ml
PD-1 PE EH12.2H7 329906 Biolegend 2.5ug/ml
IL2Rb PE 5H4 105906 Biolegend 2.5ug/ml
TCRb BV421 H57-597 109230 Biolegend 1:200
CD8 BV395 53-6.7 565968 BD Biosciences 1:200
CD4 AF488 GK1.5 100406 Biolegend 1:100
CD62L BV711 MEL-14 104445 Biolegend 1:200
CD44 BV480 IM7 566116 BD Biosciences 1:200
FOXP3 AF647 150D 320014 Biolegend 1:100

Antibodies used for the LCMV-chronic infection
Marker Fluorophore Clone #CAT Company Dilution
CD4 BUV563 RM4-5 741217 BD Biosciences 1:500
CD4 FITC RM4-5 553046 BD Biosciences 1:500

CD4 V500 RM4-5 560782 Biolegend 1:500
CD4 BV605 RM4-5 100548 Biolegend 1:500
CD4 BV711 RM4-5 100557 Biolegend 1:500
CD4 PE-Cy7 RM4-5 25-0042-82 Thermo Fischer Scientific 1:500
CD4 APC-eFluor 780 RM4-5 47-0042-82 Thermo Fischer Scientific 1:500
CD8a BUV496 53-6.7 563786 BD Biosciences 1:100
CD8a BV421 53-6.7 100753 Biolegend 1:150
CD8a BV605 53-6.7 100744 Biolegend 1:100
CD8a PerCP 53-6.7 553036 BD Biosciences 1:100
CD19 BUV563 1D3 749028 BD Biosciences 1:150
CD19 BV510 1D3 115546 Biolegend 1:150
CD19 BV605 1D3 115540 Biolegend 1:150
CD19 PE-Cy7 1D3 25-0193-82 Thermo Fischer Scientific 1:150
CD19 APC-eFluor 780 1D3 47-0193-82 Thermo Fischer Scientific 1:150
CD44 BUV805 IM7 741921 BD Biosciences 1:500
CD44 FITC IM7 561859 BD Biosciences 1:500
CD44 AF700 IM7 56-0441-82 Thermo Fischer Scientific 1:100
CD45.2 APC 104 109814 Biolegend 1:100
CD127 BUV737 SB/199 612841 BD Biosciences 1:100
CD127 PE A7R34 12-1271-83 Thermo Fischer Scientific 1:100
CD218a PE P3TUNYA 12-5183-82 Thermo Fischer Scientific 1:100
CD218a PE-Cy7 P3TUNYA 25-5183-82 Thermo Fischer Scientific 1:100
CXCR3 PE-Cy7 CXCR3-173 25-1831-82 Thermo Fischer Scientific 1:100
CXCR5 BV421 L138D7 145512 Biolegend 1:50
CX3CR1 BV785 SA011F11 149031 Biolegend 1:500
Foxp3 PE-Cy7 FJK-16s 25-5773-82 Thermo Fischer Scientific 1:250
IL-2 PE JES6-5H4 554428 BD Biosciences 1:100
IFN-γ BV421 XMG1.2 505830 Biolegend 1:100
IFN-γ APC XMG1.2 554413 BD Biosciences 1:100
PD-1 PE RMP1-30 109104 Biolegend 1:100
PD-1 APC RMP1-30 109112 Biolegend 1:100
TCF-1 PE S33-966 564217 BD Biosciences 1:100
Tim-3 BUV395 5D12 747620 BD Biosciences 1:100
Tim-3 AF488 215008 FAB1529G R&D systems 1:20
TNF-a BV421 MP6-XT22 506328 Biolegend 1:100
TNF-a PE MP6-XT22 554419 BD Biosciences 1:100

Name Clone #Catalogue
anti-mouse PD-L1 with DAPG mutation NA NA Roche 200 µg/mouse/injection
mouse IgG1 isotype control MOPC-21 BE0083 BioXcell 200 µg/mouse/injection
anti-mouse CD4 GK1.5 BE0003-1 BioXcell 300 µg/mouse/injection

Antibodies used for TILs characterization in mouse tumor-model
Marker Fluorochrome clone Cat number Provider Dilution
Fixable Viability Dye eFluor™ 455UV 65-0868-14 1:500
CD45 AF700 30-F11 103128 Biolegend 1:300
TCRb PercP-Cy5.5 H57-597 109228 Biolegend 1:200
CD8 APC-Cy7 53-6.7 100714 Biolegend 1:200
CD4 PE-Cy7 GK1.5 100422 Biolegend 1:200
CD62L FITC MEL-14 104406 Biolegend 1:200
CD127 PE A7R34 135010 Biolegend 1:100
TCRb PerCP-Cy5.5 H57-597 109228 Biolegend 1:200
CD4 BV421 GK1.5 100438 Biolegend 1:200
Granzyme B AF647 GB11 515406 Biolegend 1:100
IFN-g BV786 XMG1.2 505838 Biolegend 1:100
TNFa PE-Cy7 MP6-XT22 506306 Biolegend 1:100
FoxP3 BV421 MF-14 126419 Biolegend 1:100
CD39 AF647 Duha59 143808 Biolegend 1:200
Granzyme B AF700 QA16A02 372222 Biolegend 1:100
ki67 PE-Cy7 16A8 652426 Biolegend 1:300
PD1 PE-Cy7 RMP1-30 109110 Biolegend 1:200
CD25 BV711 RMT3-23 102049 Biolegend 1:200
TIGIT PE-Dazzle594 1G9 142110 Biolegend 1:100
IFN-g BV605 XMG1.2 505840 Biolegend 1:100
TNFa BV421 MP6-XT22 506328 Biolegend 1:100
CD107a AF488 1D4B 121608 Biolegend 1:100
CD44 BV510 IM7 563114 BD Biosciences 1:200
CD45 BUV805 30-F11 BDB748370 BD Biosciences 1:100
TCRb BV786 H57-597 742484 BD Biosciences 1:100
CD4 BUV496 RM4-5 612952 BD Biosciences 1:100
CD8 BUV395 53-6.7 563786 BD Biosciences 1:100
PD-1 BUV737 RMP1-30 749306 BD Biosciences 1:100
CD25 PE-CF594 PC61 562694 BD Biosciences 1:100
Tim3 BV650 5D12 747623 BD Biosciences 1:100
TCF1 PE S33-966 564217 BD Biosciences 1:100
LAG3 BV650 C9B7W 740560 BD Biosciences 1:100

SLAMF6 BV510 13G3 745073 BD Biosciences 1:50
CD218a FITC REA947 130-115-703 Miltenyi 1:50
OVA-dextramer H-2 Kb ((SIINFEKL) APC JD2163 Immudex 1:100
Rat anti-mouse CD8α InVivoPlus 2.43 BP0061 BioXcell 100 µg/mouse/injection

Feature barcoding
Marker Oligo Tag Clone Source Concentration (1 µg/ml)
CD28 ATTAAGAGCGTGTTG 37.51 TotalSeq-C, BioLegend 1
CD44 TGGCTTCAGGTCCTA IM7 TotalSeq-C, BioLegend 1
CD62L (L-selectin) TGGGCCTAAGTCATC MEL-14 TotalSeq-C, BioLegend 1
CD39 GCGTATTTAACCCGT Duha59 TotalSeq-C, BioLegend 1
CD279 (PD-1) GAAAGTCAAAGCACT RMP1-30 TotalSeq-C, BioLegend 1
CD366 (Tim-3) ATTGGCACTCAGATG RMT3-23 TotalSeq-C, BioLegend 1
CD223 (LAG-3) ATTCCGTCCCTAAGG C9B7W TotalSeq-C, BioLegend 1
CD183 (CXCR3) GTTCACGCCGTGTTA CXCR3-173 TotalSeq-C, BioLegend 1
CD185 (CXCR5) ACGTAGTCACCTAGT L138D7 TotalSeq-C, BioLegend 1
CD127 (IL-7Rα) GTGTGAGGCACTCTT A7R34 TotalSeq-C, BioLegend 1
TIGIT (Vstm3) GAAAGTCGCCAACAG 1G9 TotalSeq-C, BioLegend 1
CD25 ACCATGAGACACAGT PC61 TotalSeq-C, BioLegend 1
Ly108 (SLAM-F6) CGATTCTTTGCGAGT 330-AJ TotalSeq-C, BioLegend 1
CD137 (4-1BB) TCCCTGTATAGATGA 17B5 TotalSeq-C, BioLegend 1
IL-21R GATTCCGACAGTAGA 4A9 TotalSeq-C, BioLegend 1
Histology
Marker Fluorochrome clone Cat number Provider Dilution
CD3 SP7 RMAB005 Diagnostic Biosystems 1:100
CD8 4SM15 14-0808-82 eBioscience 1:300
PD-1 Polyclonal AF1021 R&D Systems 1:250
Granzyme B Polyclonal Ab4059 Abcam 1:250

Confocal Imaging
Marker Fluorochrome clone Cat number Provider Dilution
PD-1 IC D4W2J 86163S Cell Signaling Technologie 1:100
Goat anti-rabbit IgG (H+L), F(ab')2 Fragment AF488 Polyclonal 4412S Cell Signaling Technologie 1:1000
Parental PD-1 Pure 0376 NA Roche 10ug/ml
PD1-IL2v AF647 0376-IL2v fused NA Roche 1ug/ml
FAP-IL2v AF647 4B9-IL2v fused NA Roche 1ug/ml

Cell sorting
Marker Fluorochrome clone Cat number Provider Dilution
CD45 AF700 30-F11 103128 Biolegend 1:100
CD8 BV711 53-6.7 100748 Biolegend 1:100
CD4 BV605 (Bin channel) GK1.5 100451 Biolegend 1:100
CD11c BV605 (Bin channel) N418 117334 Biolegend 1:100
Live/Dead APC-Cy7 65-0865-14 eBiosciences 1:500

Validation

For FACS and CITE-seq the antibodies have been titrated (2 fold serial dilutions of the recommended concentration) on human healthy donor PBMCs or mouse splenocytes for meaningful biological patterns against common cell line markers (CD45, CD3, CD4, CD8, CD56, FOXP-3, gd T cells) and differentiation state (CD62L, CD44 and CD127) by monitoring the frequencies of positive cells (known from previous experiment and the available literature) and the background signal by using also FMO or an isotype control staining; for markers associated with exhaustion or activation (PD-1, TIM-3, LAG-3, TIGIT, CD218a, CD25) cytokine secretion (GM-CSF, Granzyme B, IFN-g and TNF-a) and IL-2R signalling (STAT-5P) manufactures' recommendations have been followed and the staining assessed on either 3 days polyclonally activated human T cells or murine splenocytes.
The selected titration for murine antibodies were then further validated on mouse TILs isolated from tumors of untreated mice.
Detailed information and references for validation and QC are shown in https://www.biolegend.com/en-us/quality/quality-control,https://www.bdbiosciences.com/en-ch/products/reagents/flow-cytometry-reagents/research-reagents/quality-and-reproducibility.

Antibodies for IHC are set up and validated using negative and positive mouse tissue and cell pellets from in-vitro 3 days activated murine T cells, isotype control antibodies and omission of primary antibody during the staining. 3 serial dilutions are tested to address the dynamic range of the assay.

For Imaging staining the intracellular PD-1 staining antibody was used at concentrations suggested by Cell Signaling Technologies (https://www.cellsignal.com/products/primary-antibodies/pd-1-d4w2j-xp-rabbit-mab/86163)

For in-vivo depletion of CD8 T cells with Rat anti-mouse CD8α InVivoPlus clone 2.43 from BioXcell we referred to Lin J-S, Szaba FM, Kummer LW, Chromy BA, and Smiley ST. 2011. J. Immunol. 187: 897-904, Wozniak KL, Young ML, and Wormley FL. 2011. Clin. Vaccine Immunol. 18(5):717-723 and Hufford MM, Kim TS, Sun J, and Braciale TJ. 2011. J. Exp. Med. 208: 167-180.

For in-vitro experiments immunocytokines (PD1-IL2v, FAP-IL2v, nbPD1-IL2v) were used at EC50 concentration (630 pM) obtained from the dose response IL-2R signaling curves on 3 days polyclonally activated CD4 T cells.

Anti-mouse PD-L1 antibody with DAPG mutation was validated by Roche and the previous study (Klein, C. et al. Cergutuzumab amunaleukin (CEA-IL2v), a CEA-targeted IL- 2 variant-based immunocytokine for combination cancer immunotherapy: Overcoming limitations of aldesleukin and conventional IL-2-based immunocytokines. Oncoimmunology 6, e1277306,

doi:10.1080/2162402X.2016.1277306 (2017)).

Eukaryotic cell lines

Policy information about cell lines and Sex and Gender in Research

Cell line source(s)	Vero E6 cells (ATCC)<br>Panc02-H7-Fluc cell line was generated at Roche Glycart.<br>B16-OVA cell line was purchased from ProQinase.<br>SCC173 Sigma-Aldrich MCA205 Mouse Fibrosarcoma Cell Line: MCA-205 was derived from 3-methylcholanthrene-induced fibrosarcoma in C57BL/6 mice. Tumors were maintained in vivo by serial subcutaneous transplantation in syngeneic mice and single-cell suspensions were prepared from solid tumors by enzymatic digestion. From these cells the MCA-205 cell line was established and maintained in vitro.
Authentication	Vero E6 cells were not authenticated.<br>MCA-205, B16-OVA and Panc02-H7-Fluc were authenticated through morphology and PCR assays with species specific primers.
Mycoplasma contamination	MCA-205: Cells are tested negative for infectious diseases by a Mouse Essential CLEAR panel by Charles River Animal Diagnostic Services: cells are negative for mycoplasma contamination.<br>Panc02-H7-Fluc cell line and B16-OVA cell line batches are routinely tested for mycoplasma and are negative.
Commonly misidentified lines<br>(See ICLAC register)	No commonly misidentified cell lines were used in this study

Animals and other research organisms

Policy information about studies involving animals; ARRIVE guidelines recommended for reporting animal research, and Sex and Gender in Research

Laboratory animals	Six- to 8-week-old female C57BL/6J and CD45.1 congenic mice were purchased from the Jackson Laboratory for chronic infection model and Charles Rivers, Lyon, France for the mouse tumor models except the RIP-Tag mouse tumor model performed on males aged from 21 to 31 weeks.<br>For chronic infection model:<br>All animal experiments were performed in accordance with National Institutes of Health and the Emory University Institutional Animal Care and Use Committee guidelines.<br><br>The following housing conditions for the mice are used.<br>For the chronic infection experiments:<br>-Light Cycle is 7:00 am ON, 7:00 pm OFF<br>-Temperature is between 68-74 degrees Fahrenheit<br>-Humidity is between 30-70 g/m3<br>For the mouse tumor model:<br>Mice were maintained under specific-pathogen-free conditions with daily cycles of 12 hours light/darkness according to guidelines (temperature of 22°C, dark/light cycle of 12h, and humidity of 50%, GV-SOLAS; FELASA) and food and water were provided ad libitum. Continuous health monitoring was carried out and the experimental study protocol was reviewed and approved by the Veterinary Department of Canton Zurich.
Wild animals	No wild animals were used
Reporting on sex	Female mice were used in the chronic infection and tumor models
Field-collected samples	No field collection was performed
Ethics oversight	For the chronic infection: All animal experiments were performed in accordance with National Institutes of Health and the Emory University Institutional Animal Care and Use Committee guidelines.<br>For mouse tumor models: experimental study protocol was reviewed and approved by the Veterinary Department of Canton Zurich.<br>For the RIP-Tag mouse tumor model Animal experiments were conducted according to protocols approved by the Veterinary Authorities of the Canton of Vaud and the Swiss Law.

Note that full information on the approval of the study protocol must also be provided in the manuscript.

Flow Cytometry

Plots

Confirm that:

☒ The axis labels state the marker and fluorochrome used (e.g. CD4-FITC).

☒ The axis scales are clearly visible. Include numbers along axes only for bottom left plot of group (a 'group' is an analysis of identical markers).

☒ All plots are contour plots with outliers or pseudocolor plots.

☒ A numerical value for number of cells or percentage (with statistics) is provided.

Methodology

Sample preparation

Human PBMC isolation
Blood samples from healthy volunteers were obtained via the blood donation center (Zürich, Switzerland) with approval of the Cantonal Ethics Committee (Zürich). PBMC were isolated from the blood of different healthy donors using density gradient centrifugation with Histopaque-1077 (Sigma). All cells were cultured in RPMI 1640 (Gibco) suplemented with 10% heat-inactivated FBS (Gibco), GlutaMAX (Gibco), and 1% penicillin-streptomycin 100× (Gibco).

Human and murine CD4 T cell isolation and in vitro activation
Human CD4T cells were sorted by using a CD4-positive selection Miltenyi beads system following manufacturer instructions. Thereafter the cells were labelled with CFSE (5 µM, 5 min at RT, eBioscience) or CTV (5 µM, 5 min at room temperature, Thermo Scientific) to measure cell proliferation.
CD4 T cells were seeded into an αCD3 pre-coated plate (1 µg/ml, clone OKT3, BioLegend overnight, 4°C) with addition of soluble αCD28 (1 µg/ml, clone CD28.2, BioLegend). The cells were cultured for 3 days to induce activation and upregulation of the PD-1 receptor on the surface of the CD4 T cells.
Spleens of C57BL/6 mice were homogenized to a single cells suspension by mashing the spleen through a 100 uM cells strainer and the erythrocytes were lysed with ACK (ammonium-chloride-potassium) lysis buffer for 5 min at 4°C. CD4 T cells were sorted with a CD4-negative selection Miltenyi beads system following manufacturer instructions. CD4 T cells were seeded into an αCD3/ αCD28 pre-coated plate (5 µg/ml, clone 145-2C11, BioLegend and 5 µg/ml, clone 37.51 BioLegend) and activated for 3 days.

Binding competition on Treg and Tconv and Treg suppression assay
CD4+ CD25+ CD127dim regulatory T cells (Tregs) were isolated from human peripheral blood with the two-step regulatory T cell Isolation Kit (Miltenyi). In parallel the CD4+ CD25- conventional T cells (Tconv) were isolated by collecting the negative fraction of a CD25-positive selection (Miltenyi) followed by CD4+ enrichment (Miltenyi). Tconv were labelled with CFSE and the Tregs were labelled with Cell Trace Violet to track the proliferation of both populations.
For the PD-1 and IL2Rb receptor quantification and the PD1-IL2v binding competition, Tregs and Tconv were cocultured at a 1:1 ratio into a αCD3 pre-coated plate (1 µg/ml, clone OKT3, BioLegend) with soluble αCD28 (1 µg/ml, clone CD28.2, BioLegend).
In the Treg suppression assay, the rescue of Tconv granzyme B production upon PD1-IL2v treatment was measured upon co-culturing Tconv together with Treg at 2:1 ratio for 5 days, in presence or absence of treatment. Irradiated (40 Gy) feeders from an unrelated donor where used to elicit an allospecific stimulation.

GM-CSF, granzyme-B and IFN-g secretion by CD4 T cells
Sorted and CTV labelled human polyclonal CD4 T cells were activated with soluble αCD3 (1 µg/ml) in presence of irradiated (40 Gy) feeder cells from the same donor at 1:1 ratio and increasing concentrations of treatment antibodies or aldesleukin (Proleukin, Novartis). After 5 days, GM-CSF secretion was measured with ELISA (BioLegend) following manufacturer instructions. For intracellular FACS staining, the accumulation of cytokines in the Golgi complex was induced by re-stimulating the cells with ionomycin (500 ng/ml) and PMA (50 ng/ml) together with protein transport inhibitors (GolgiPlug and GolgiStop, BD) for 5 hours prior to the FACS staining.

Binding competition
3 days activated CD4 T cells were exposed to increasing equimolar concentrations of either PD1-IL2v, pembrolizumab or non-blocking PD1-IL2v for 30 min at 4°C. After a washing step, the cells were incubated for additional 30 min at 4°C with saturating concentrations of an Alexa Fluor-647 directly conjugated parental PD-1 antibody used to generate PD1-IL2v. The cells were fixed with Cell fix (BD) after an additional washing.

Flow cytometry staining for cytokine detection and receptor quantification
The cells were stained in PBS with surface antibodies for 30 min at 4°C and for being live/dead (either Aqua Dead Cell Stain, Invitrogen, during the last 10 min of incubation, or Fixable Viability Dye eFluor 780, eBioscience, for 30 min, 4°C). For intracellular staining, cells were permeabilized with FACS permeabilization buffer (fixation/permeabilization, BD Biosciences or Foxp3 Transcription Factor Fixation kit, eBioscience) and then incubated with antibodies specific for cytokines for 60 min at 4°C. The following antibodies mixes were used:
1) human: PD1 (clone EH12.2H7 BioLegend, IL2Rb (clone TU27, BioLegend), isotype control (clone MOPC-21, BioLegend), CD4 (clone RPA-T4, eBioscience), GM-CSF (clone BVD2-21C11, BioLegend), GrzB (GB11, BD Biosciences), IFNg (clone 4S.B3, eBioscience)
2) mouse: PD1 (clone 29F.1A12 BioLegend), IL2R (clone 5H4, BioLegend), isotype control (clone RTK2758, BioLegend), TCR-β (clone H57-597, Biolegend), CD3 (clone 145-2C11, BioLegend), CD8 (clone 53-6.7, BD), CD4 (clone GK1.5, BioLegend), CD45 (clone 30-F11, BioLegend), CD62L (clone Mel-14, BioLegend), CD44 (clone IM7, BD), FoxP3 (clone 150D, BioLegend).
The number of PD-1 and IL-2Rβ receptors were quantified on the cell surface of PBMCs and TILs of huPD1 transgenic mice bearing Panc02-H7-Fluc tumors and on human activated Tregs and Tconv with the PE Phycoerythrin Fluorescence

Quantitation Kit (BD) following manufacturer's instructions. 2.5 µg/ml of PE-labeled monoclonal antibodies were used to quantify the receptor of interest on gated populations of interest. The cells and the PE Quantibrite beads were fixed following the same protocol and fluorescence data acquired while using the same settings. The number of receptors was quantified following the kit instructions.

Ex-vivo binding of PD1-IL2v, FAP-IL2v and FAP-Superkine-analogue was performed by incubating 630 pM of the constructs for 30 min on healthy donors PBMCs. After a washing step the cells were incubated for an additional 30 min at 4°C with a PE-labelled antibody recognizing the PGLALA mutation in the Fc-portion of the primary antibodies together with a panel of antibodies to characterize the phenotype of the immune-cytokine targeted cells: CD3 (clone OKT3), CD4 (clone OKT4), CD8 (clone RPA-T8), TIM-3 (clone F38-2E2), CD218a (clone H44), CD56 (clone NCAM16.2), TCF-1 (C63D9) FOXP3 (clone 206D) and PD-1 (clone D4W2J).

Lymphocyte isolation
1. For chronic infection experiments
Lymphocytes were isolated from the blood, spleen and lung as described previously52. Briefly, spleens were dissociated by passing them through a 70 µm cell strainer (Corning). Lungs were treated with 1.3 mM EDTA in HBSS for 30 min at 37°C, shaking at 200 rpm, followed by treatment with 150 U/ml collagenase (Thermo Fisher Scientific) in RPMI 1640 medium containing 5 % FBS, 1 mM MgCl2, and 1mM CaCl2 for 60 min at 37°C shaking at 200 rpm. Collagenase treated lung tissues were homogenized and filtered through a 70 µm cell strainer. Lymphocytes from lungs were purified by a 44–67% Percoll gradient (800 g at 20°C for 20 min).
2. For cancer model experiments
tumor tissue and blood were isolated in the animal facility. The tumor tissue was transferred into PBS and was disrupted using manual scissors and the Miltenyi Gentle MACS machine. Subsequently, it was digested in an enzyme mix containing RPMI with 10 mg/ml DNAse (Sigma Aldrich) and 0.25 mg/ml Liberase (Sigma Aldrich). Upon 30 min of digestion at 37°C, the tissue mix was filtered through a 70 µm filter and resuspended to a single-cell suspension with an appropriate dilution for subsequent fluorescently labeled antibody staining. The blood was transferred in heparin tubes and was lysed with erythrocyte lysis buffer. Upon red blood lysis, cells were resuspended to a single-cell suspension with an appropriate dilution for subsequent fluorescently labeled antibody staining. Lymphocytes were mechanically isolated from draining lymph nodes with a pestel, filtered through a 70 µm filter and resuspended to a single-cell suspension with an appropriate dilution for subsequent fluorescently labeled antibody staining.

For detection of cytokines, tumor cell suspensions were restimulated with 6.25 ng/ml of PMA (Sigma Aldrich) and 1.87 µg/ml of ionomycin (Sigma Aldrich) for 5h at 37°C. Upon 1 hour of restimulation, Golgiplug (BD) and Golgistop (BD) were added in the cell suspensions. For antigen restimulation, tumor cell suspensions were restimulated with 0.1 ug/ml of gp100 or SIINFEKL peptide, for 5 hours at 37°C. Anti-CD107a antibody was added together with the peptides for 5h at 37°C. As before, upon 1 hour of restimulation, Golgiplug (BD) and Golgistop (BD) were added in the cell suspensions.
Discrimination of living cells versus dead cells was performed using DAPI (Sigma Aldrich) for the non-fixed samples and Fixable Viability Dye eFluor™ 780 (eBioscience) for the fixed ones. Samples were acquired with a BD LSRIIFortessa and a BD FACS Symphony A5. Data obtained were analyzed by using FlowJo (v10.8.1, BD Biosciences).

Cell sorting
1. For chronic infection experiments
Cell sorting was performed by a FACS Aria II (BD Biosciences). For adoptive transfer experiments, two PD-1 expressing CD8+ T cell-subsets (PD-1+CXCR5+Tim-3- and PD-1+CXCR5-Tim-3+) were sorted from pooled spleens (n=40-60) of chronically LCMV infected mice. For RNA-seq analysis of LCMV-specific CD8+ T cells after muPD-L1, muPD1-IL2v, and muPD-L1 + PD1-IL2v therapy, chronically LCMV infected mice (> day 40 post-infection n=1-18) were untreated or treated for 2 weeks, and DbGP33+ CD8+ T cells were sorted from pooled spleens for obtaining at least 2x104 cells. Naïve (CD44lo) CD8+ T cells were sorted from pooled spleens of uninfected mice (n=2-3). All samples had purities of greater than 95%.
2. For cancer model experiments
Single cell tumor suspensions were kept on ice during the staining and sorting procedure. Cell suspensions from 3-5 tumors of the same treatment group were pooled and incubated with Fc receptor block (BD, #101320) for 10 minutes, before staining with the following antibodies: Alexa Fluor 700 anti-CD45 (30-F11), BV711 anti-CD8 (53-6.7), BV605 anti-CD4 (GK1.5), BV605 anti-CD11c (N418). Discrimination of living cells versus dead cells was performed using DAPI (Sigma Aldrich) for the non-fixed samples and cells incubated for 20 minutes. Cells were washed twice, filtered through a 40um cell strainer and sorted on FACS AriaIII to enrich the viable, single, CD45+ CD8+ CD11c- CD4- population.

Detailed isolation and sample handling are described in the Method section.

Instrument

Cell sorting was performed by FACS Aria II or Aria III (BD Biosciences).
FACS data was collected on BD Canto II, LSR II Fortessa, FACS Symphony A3 or A5.

Software

FACSDiva v8.0.1 or 9.1, FlowJo v. 9.9.6 or 10.8.1 (BD Biosciences)

Cell population abundance

The purities of the sorted cells were more than 95%.

Gating strategy

Gate boundaries were set accordingly to control samples (FMO- fluorescence minus one or isotype controls) or based on density distribution. Gating examples are provided as supplemetary information.

☒ Tick this box to confirm that a figure exemplifying the gating strategy is provided in the Supplementary Information.
